# Activation of AMPK as a therapeutic strategy for FBXL4-related mitochondrial DNA depletion syndrome

**DOI:** 10.1038/s44321-026-00471-z

**Published:** 2026-06-17

**Authors:** Aniketh Bishnu, Juan Zapata-Muñoz, Robert W Taylor, Kei Sakamoto, Ian G Ganley

**Affiliations:** 1 MRC Protein Phosphorylation and Ubiquitylation Unit, School of Life Sciences, University of Dundee, Dundee, DD1 5EH Scotland UK; 2 Mitochondrial Research Group, Translational and Clinical Research Institute, Faculty of Medical Sciences, Newcastle University, Newcastle upon Tyne, UK; 3 NHS Highly Specialised Service for Rare Mitochondrial Disorders, Newcastle upon Tyne Hospitals NHS Foundation Trust, Newcastle upon Tyne, NE1 4LP UK; 4 Novo Nordisk Foundation Center for Basic Metabolic Research, University of Copenhagen, Copenhagen, 2200 Denmark

**Keywords:** Genetics, Gene Therapy & Genetic Disease, Organelles

## Abstract

Distinct mitophagy pathways can eliminate not only damaged mitochondria but also healthy ones. In Mitochondrial DNA Depletion Syndrome 13 (MTDPS13), dysregulated BNIP3/NIX-driven mitophagy of functional mitochondria is thought to be the key pathological driver. Patient mutations in the E3 ubiquitin ligase FBXL4 impair the proteasomal degradation of the mitophagy receptors BNIP3 and NIX, causing their accumulation and excessive mitophagy. As a result, mitochondrial content and oxidative phosphorylation decline sharply across multiple tissues, leading to early mortality, with no effective treatments currently existing. Here, we build on our work showing that AMPK can inhibit mitophagy via sequestration of the ULK1 autophagy-initiating kinase ULK1 and demonstrate that it is also critically relevant for mitophagy induced by FBXL4 disruption. Using FBXL4-deficient cells, as well as fibroblasts derived from MTDPS13 patients and a chemically-induced mouse model, we show that small molecule AMPK activation inhibits BNIP3/NIX-mediated mitophagy and recovers functional mitochondrial content. This work therefore validates AMPK as a realistic target in treating MTDPS13.

## Synopsis

Pharmacological activation of AMPK suppresses pathological BNIP3/NIX-mediated mitophagy, restores mitochondrial function, and represents a promising therapeutic strategy for mitochondrial DNA depletion syndrome 13 (MTDPS13).

- AMPK activation inhibits excessive BNIP3/NIX-mediated mitophagy and restores mitochondrial content and respiration in FBXL4-deficient cells.
- AMPK activation promotes mitochondrial biogenesis, further contributing to the recovery of mitochondrial homeostasis.
- In a chemically induced mouse model of MTDPS13, pharmacological AMPK activation suppresses mitophagy in both liver and brain tissue.

## Abstract (web summary)

Pharmacological activation of AMPK suppresses pathological BNIP3/NIX-mediated mitophagy, restores mitochondrial function, and represents a promising therapeutic strategy for mitochondrial DNA depletion syndrome 13 (MTDPS13).

## Graphical abstract

**Figure:**
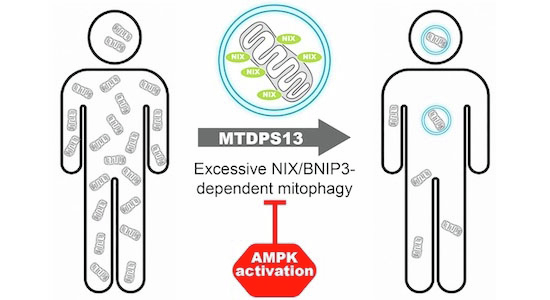

**Figure:**
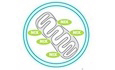

The paper explained **Problem**

Mitochondrial DNA depletion syndrome (MTDPS13) is a rare genetic disease caused by mutations in the *FBXL4* gene. This results in upregulated NIX/BNIP3-mediated degradation of functional mitochondria via a process called mitophagy, which is considered the central driver of disease pathogenesis. The resulting mitochondrial loss results in profound oxidative phosphorylation and cellular energy deficiency, ultimately leading to multi-organ failure and early mortality. Currently, no effective therapeutic options are available for individuals affected by MTDPS13.

**Results**

We investigated the possibility of targeting the key energy-sensing kinase AMPK to inhibit NIX/BNIP3-dependent mitophagy in the context of MTDPS13. Using genetically engineered and MTDPS13 patient-derived cell line models, we demonstrate that pharmacological activation of AMPK can inhibit FBXL4-associated mitophagy and restore mitochondrial content. AMPK activation also restored mitochondrial respiration levels to that of healthy wild-type cells. In a chemically induced mouse model of MTDPS13, we show that pharmacological AMPK activation can inhibit mitophagy in the liver and the brain. Furthermore, we demonstrate that PXL-770, an AMPK activator with favorable Phase 2 clinical trials for safety and tolerability, significantly inhibited FBLX4-associated mitophagy and restored mitochondrial content to similar levels of wild-type cells.

**Impact**

Our study provides preclinical evidence for targeting AMPK as the first promising therapeutic strategy for MTDPS13. This study established the proof-of-principle of pharmacologically targeting AMPK and provides a strong rationale for the clinical evaluation of existing AMPK activators in MTDPS13, as well as for the development of new AMPK modulators.

## Introduction

Mitophagy refers to several pathways that eliminate mitochondria via the autophagic-lysosomal system (Ganley and Simonsen, 2022; Montava-Garriga and Ganley, 2020). Traditionally, it is viewed as a quality control mechanism that eliminates damaged mitochondria to help maintain cellular homeostasis. Impaired mitophagy results in the accumulation of dysfunctional mitochondria, a hallmark of several neurodegenerative diseases and other pathologies (Martinez-Vicente, 2017; Stanojlovic et al, 2022). The best-characterized pathway in this context is the Parkinson’s disease–associated PINK1-Parkin cascade, which selectively targets damaged mitochondria for degradation (Goodall et al, 2022; Narendra and Youle, 2024). However, mitophagy is likely not restricted to damaged/dysfunctional organelles. Activation of the NIX/BNIP3 pathway facilitates mitochondrial turnover in the apparent absence of overt mitochondrial dysfunction, for example, during processes such as erythrocyte maturation as well as differentiation of various cell types ranging from neurons to cardiomyocytes (Esteban-Martinez et al, 2017; Ordureau et al, 2021; Sandoval et al, 2008; Zhao et al, 2020). Notably, hyperactivation of NIX/BNIP3-driven mitophagy results in pronounced mitochondrial depletion, as observed in FBXL4-related mitochondrial DNA depletion syndrome (MTDPS13) (Cao et al, 2023; Elcocks et al, 2023; Nguyen-Dien et al, 2023). This fatal level of organelle loss implies that functional mitochondria are being turned over. In line with this, our recent work has shown that mitochondria targeted by NIX-mediated mitophagy maintain functional capacity compared to those degraded by the PINK1-Parkin pathway (Longo et al, 2024).

MTDPS13 (OMIM 615471) is a rare human mitochondrial disorder caused by bi-allelic pathogenic variants mutations in the *FBXL4* gene (Bonnen et al, 2013; Gai et al, 2013). FBXL4 (F-box/LRR-repeat protein 4) is a mitochondrial outer membrane protein belonging to the F-box protein family and is a component of the SCF (SKP1-CUL1-F-box protein) subfamily of Cullin-RING E3 ubiquitin ligases (CRLs). Within this complex, Cullin 1 provides the structural scaffold, RBX1/2 recruits the E2 ubiquitin-conjugating enzyme, SKP1 functions as the adaptor, and FBXL4 serves as the substrate recognition subunit (Skaar et al, 2013). FBXL4 was recently found to interact with partially redundant mitophagy receptors NIX and BNIP3, facilitating their ubiquitination and proteasomal degradation (Elcocks et al, 2023; Nguyen-Dien et al, 2023; Park et al, 2023). This action limits the abundance of NIX and BNIP3, thereby restraining basal mitophagy. Consistent with this, loss of the phosphatase PPTC7 (Protein Phosphatase Targeting COQ7), which acts as an essential adaptor for SCF-FBXL4-NIX/BNIP3 assembly, phenocopies FBXL4 knockout cells (Nguyen-Dien et al, 2024; Niemi et al, 2023; Sun et al, 2024). FBXL4-deficient or mutant cells exhibit elevated NIX and BNIP3 levels, which drive excessive mitophagy and mitochondrial depletion. The resulting loss of mitochondrial mass leads to severe oxidative phosphorylation (OXPHOS) deficiency and broad metabolic disruption (Antoun et al, 2016). In vivo, *FBXL4* knockout mice demonstrate increased mitophagic flux, reduced mitochondrial content, and perinatal lethality (Alsina et al, 2020). Likewise, patients with deleterious, segregating FBXL4 variants typically present with early-onset disease characterized by profound neurodevelopmental delay, encephalomyopathy, hypotonia, and greatly reduced life expectancy (Ballout et al, 2019; El-Hattab et al, 2017; Huemer et al, 2015). At present, there are no effective treatments available for FBXL4-associated MTDPS13, although modulation of NIX-dependent mitophagy represents a potential therapeutic avenue.

We recently identified AMPK activation as an inhibitor of NIX-mediated mitophagy (Longo et al, 2024). AMPK is a key regulator of cellular energy homeostasis and a known modulator of autophagy (Steinberg and Hardie, 2023). It functions as a heterotrimeric kinase complex consisting of a catalytic α subunit (two isoforms), a regulatory β subunit (two isoforms), and a γ subunit (three isoforms). While AMPK activation has classically been associated with the stimulation of autophagy, recent findings indicate that AMPK can also inhibit macroautophagy under certain conditions (Kazyken et al, 2024; Nwadike et al, 2018; Park et al, 2023). Our previous studies revealed that AMPK phosphorylates and inhibits ULK1, the autophagy-initiating kinase, promoting its sequestration by 14-3-3 proteins and thereby blocking initiation of NIX/BNIP3-dependent mitophagy—in both cell culture models and in mouse tissues. Importantly, AMPK continues to promote clearance of damaged mitochondria through PINK1-Parkin mitophagy, independently of ULK1 (Longo et al, 2024).

Of relevance, AMPK activation can also positively regulate mitochondrial biogenesis, in large part via upregulation of the transcriptional co-activator, peroxisome proliferator-activated receptor gamma co-activator 1α (PGC1α)—a master regulator of mitochondrial biogenesis (Handschin and Spiegelman, 2006). AMPK achieves this via multiple mechanisms, including phosphorylation of Folliculin-Interacting Protein 1, which results in nuclear translocation of transcription factor-EB (TFEB) and/or transcription factor binding to IGHM enhancer 3 (TFE3), which in turn leads to increased PGC1α levels and mitochondrial biogenesis (Malik et al, 2023). Therefore, given AMPK’s dual role in suppressing NIX/BNIP3-driven mitophagy and enhancing mitochondrial biogenesis, we hypothesized that pharmacological activation of AMPK could counteract the pathological overactivation of NIX-mediated mitophagy observed in FBXL4-related MTDPS13. Several direct AMPK activators are under clinical evaluation for conditions including cardiometabolic diseases, metabolic dysfunction–associated steatotic liver disease and steatohepatitis, and polycystic kidney disease (Cusi et al, 2021; Dagorn et al, 2023; Holm et al, 2025; Steinberg and Carling, 2019). However, whether these compounds can mitigate the excessive mitophagy caused by FBXL4 deficiency has not yet been determined. Accordingly, we aimed to assess whether AMPK activators can restore mitochondrial mass and function by inhibiting NIX/BNIP3-mediated mitophagy and/or enhancing mitochondrial biogenesis in the context of FBXL4-related MTDPS13.

## Results

### Pharmacological activation of AMPK blocks mitophagy induced by FBXL4 inhibition

**Figure 1:**
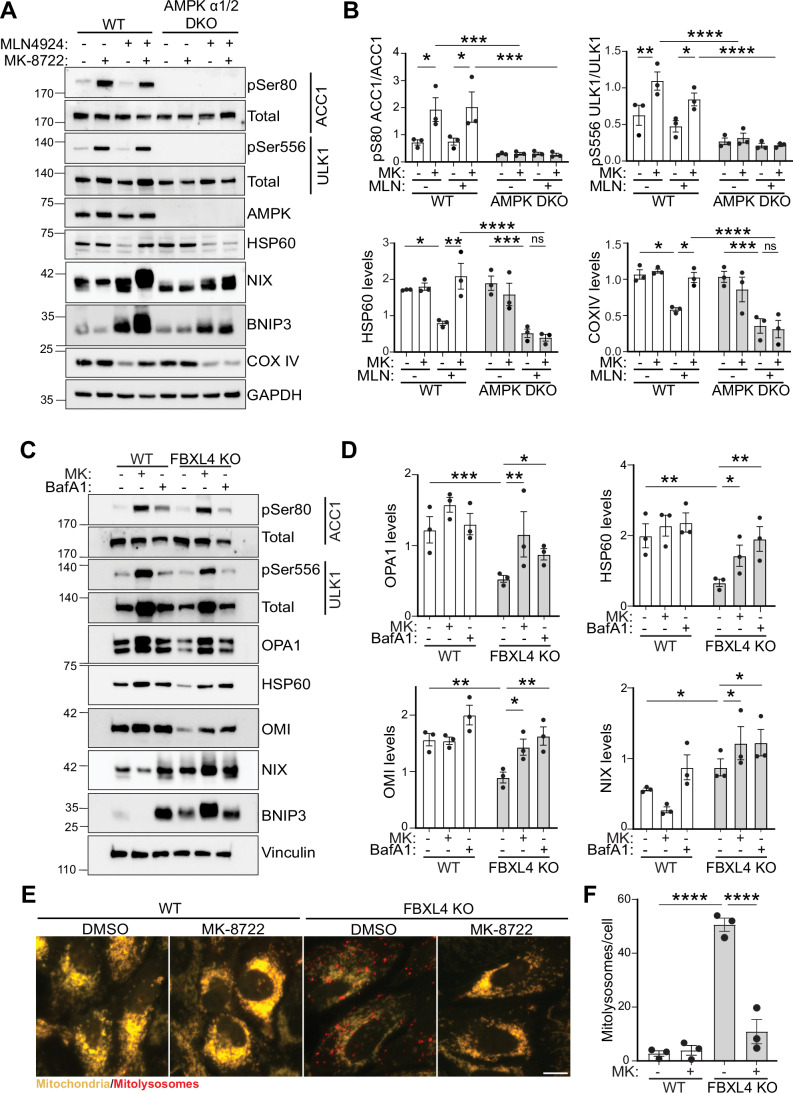
AMPK activation inhibits FBXL4-induced mitophagy. (**A**) Representative immunoblots of indicated proteins from ARPE-19 wildtype (WT) or AMPKα double knockout (AMPK DKO) treated with MLN4924 (5 μM) and/or MK-8722 (10 μM) for 24 h. (**B**) Quantification of the immunoblotting data in panel (**A**) with protein levels normalized to loading controls. Data represents mean ± SEM (*n* = 3), significance calculated using ordinary two-way ANOVA and Sidak’s multiple comparisons test. (**C**) Representative immunoblots of indicated proteins in ARPE-19 wildtype (WT) and FBXL4 knockout (FBXL4 KO) treated with MK-8722 (10 μM) for 48 h and/or Bafilomycin A1(BafA1, 50 nM) for the final 24 h. (**D**) Quantification of the immunoblotting data in panel C. Data represents mean ± SEM (*n* = 3), significance calculated using ordinary two-way ANOVA and Tukey’s multiple comparisons test. (**E**) Representative wide-field images of WT and FBXL4 KO ARPE-19 *mito*-QC cells, treated with MK-8722 (10 μM) for 48 h. Red puncta represent mitolysosomes while the mitochondrial network appears yellow. Scale bar: 20 μm. (**F**) Quantification of the number of mitolysosomes (red puncta) per cell. Data represents mean ± SEM (*n* = 3), significance calculated using ordinary two-way ANOVA and uncorrected Fisher’s LSD test. For all the experiments, statistical significance is depicted as *p < 0.05, **p < 0.01, ***p < 0.001, ****p < 0.0001, and ns nonsignificant. Source data are available online for this figure.

**Figure EV1:**
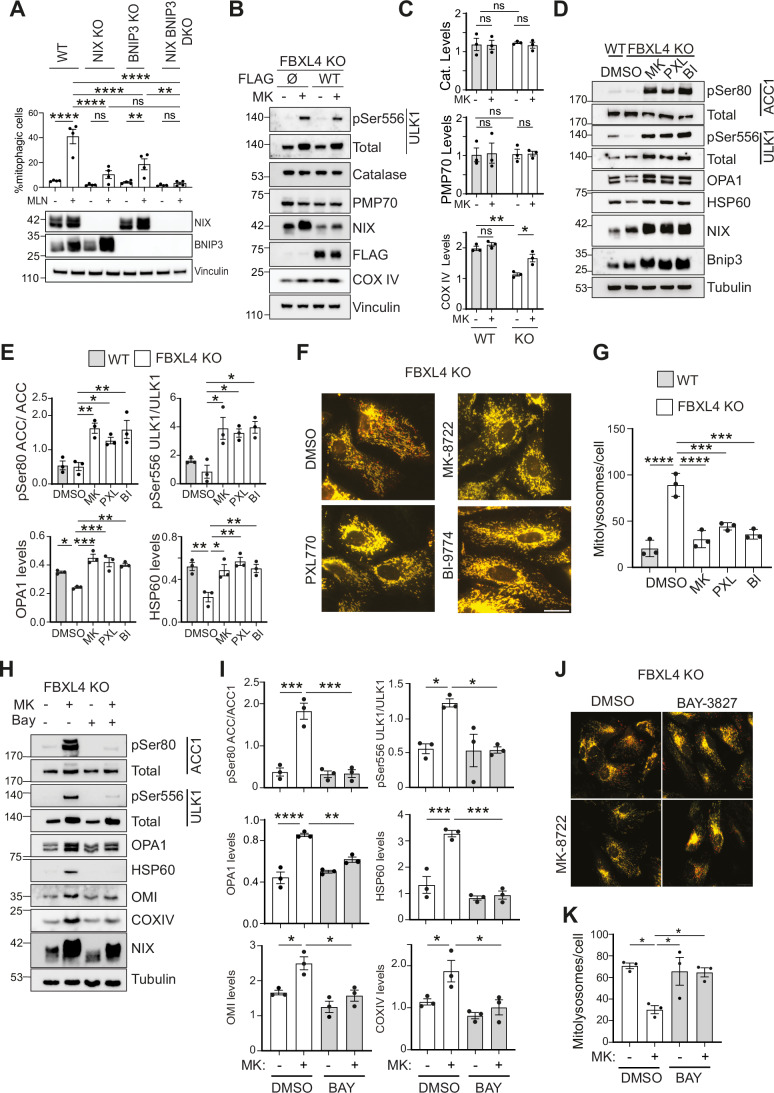
AMPK activators inhibit FBXL4-induced mitophagy. (**A**) Flow cytometry analysis of WT, NIX knockout (NIX KO), BNIP3 knockout (BNIP3 KO) and NIX and BNIP3 double knockout (NIX BNIP3 DKO) treated with 5 μM of MLN4924 for 24 h. The percentage of ARPE-19 cells having a high mCherry/GFP ratio was quantified for each condition. Data represents mean ± SEM (*n* = 4). Significance calculated using ordinary two-way ANOVA and Sidak’s multiple comparisons test. Representative blot of NIX and BNIP3 in knockout cells is shown below. (**B**) Representative immunoblot of indicated protein in FBXL4 knockout (FBXL4 KO) and WT rescue cells treated with/without MK-8722 (MK, 10 μM). (**C**) Quantification of the immunoblot data in panel (**B**) of protein levels normalized to loading controls. Data represents mean ± SEM (*n* = 3), significance calculated using two-way ANOVA and Uncorrected Fisher’s LSD. (**D**) Representative immunoblot of indicated proteins from ARPE-19 wildtype (WT) and FBXL4 knockout (FBXL4 KO) treated with/without MK-8722 (MK, 10 μM), PXl-770 (PXL, 50 μM), and BI-9774 (BI, 10 μM) for 48 h. (**E**) Quantification of the immunoblot data in panel D with protein levels normalized to loading controls. Data represents mean ± SEM (*n* = 3), significance calculated using ordinary one-way ANOVA and Tukey’s multiple comparisons test. (**F**) Representative wide-field images of FBXL4 KO ARPE-19 *mito*-QC cells, treated as in D. Red puncta represent mitolysosomes while mitochondrial network appears yellow. Scale bar: 20 μm. (**G**) Mitolysosomes quantitation per cell. Data represents mean ± SEM (*n* = 3), significance calculated using ordinary one-way ANOVA and Tukey’s multiple comparison test. (**H**) Representative immunoblots of indicated proteins from FBXL4 knockout (FBXL4 KO) cells treated with MK-8722 (MK, 10 μM) and BAY-3827 (5 μM) for 48 h. (**I**) Quantification of the immunoblot data in panel (**H**) with protein levels normalized to loading controls. Data represents mean ± SEM (*n* = 3), significance calculated using ordinary one-way ANOVA and Tukey’s multiple comparisons test. (**J**) Representative wide-field images of WT and FBXL4 KO ARPE-19 mito-QC cells, treated with MK-8722 (MK, 10 μM) and BAY-3827 (5 μM) for 48 h. Red puncta represent mitolysosomes while mitochondrial network appears yellow. Scale bar: 20 μm. (**K**) Quantification of the number of mitolysosomes per cell. Data represents mean ± SEM (*n* = 3), significance calculated using ordinary one-way ANOVA and Tukey’s multiple comparison test. For all the experiments, statistical significance is depicted as *p < 0.05, **p < 0.01, ***p < 0.001, and ****p < 0.0001.

The levels of NIX and BNIP3 govern the degree of basal mitophagy in cells and are regulated at both the transcriptional level, for example by HIF1α (Allen et al, 2013; Sowter et al, 2001; Zhao et al, 2020), and post translational level, via FBXL4-mediated ubiquitylation and proteasomal degradation (Cao et al, 2023; Elcocks et al, 2023; Nguyen-Dien et al, 2023). As we have previously shown that pharmacological AMPK activation can block NIX/BNIP3 mitophagy induced by HIF1-mediated transcription (Longo et al, 2024), we asked whether AMPK activation could also block NIX/BNIP3-mediated mitophagy caused by inhibition of FBXL4. We have previously demonstrated ARPE-19 cells as a tractable model for evaluating NIX-mediated mitophagy (Montava-Garriga et al, 2020), and so we treated these with MLN4924, an inhibitor of Cullin Neddylation and hence FBXL4-CRL function (Elcocks et al, 2023; Soucy et al, 2009). Although MLN4924 is a global SCF E3 ligase inhibitor, and will also block the Von Hippel-Lindau (VHL)-CRL that downregulates HIF1-mediated mitophagy (Ganley and Simonsen, 2022), it was shown in cells that MLN4924 mediated increase in NIX was specifically via FBXL4 inhibition (Elcocks et al, 2023). Upon treatment, and as expected, we observed increased levels of NIX and BNIP3 along with a concomitant reduction in the mitochondrial proteins HSP60 and COXIV, in both wild-type (WT) and AMPKα1/α2 double knockout (DKO) cells (Fig. 1A,B). This indicates SCF inhibition induces NIX/BNIP3-dependent mitophagy in these cell lines. Treatment of cells with MK-8722, a potent and selective pan AMPK activator (Myers et al, 2017; Negoita et al, 2026), significantly increased AMPK substrate phosphorylation (ACC1 Ser80 and ULK1 Ser556) in WT but not in DKO cells and had little effect on mitochondrial levels when used alone. In contrast, when used in combination with MLN4924, MK-8722 completely blocked the MLN4924-induced reduction in mitochondrial proteins. This only happened in AMPK WT, but not DKO, cells, indicating AMPK activation by MK-8722 blocks NIX/BNIP3-mediated mitophagy in the context of pharmacological SCF (and hence FBXL4) inhibition. Next, we wanted to evaluate the relative contribution of NIX and BNIP3 in regulating MLN4924-induced mitophagy in ARPE-19 cells. We generated single and double knockout (DKO) NIX and BNIP3 cells using CRISPR-Cas9 gene editing. As before, MLN4924 significantly increased mitophagy in the WT cells. A smaller but significant increase in mitophagy was also observed in BNIP3 KO cells, in contrast to NIX KO, while mitophagy was completely abrogated in the DKO cells (Fig. EV1A). While this suggests there is a small degree of redundancy between the two receptors, NIX appears to be the predominant mitophagy regulator in ARPE-19 cells.

To specifically evaluate the effect of AMPK activation of FBXL4-induced mitophagy, we generated FBXL4 knockout (KO) ARPE-19 cells. There are currently no validated and commercially available antibodies to detect endogenous FBXL4, yet genomic sequencing revealed the selected clone had a homozygous frameshift mutation leading to the generation of a premature stop at the 183^rd^ codon (See Methods). FBXL4 KO cells displayed increased NIX and BNIP3 levels, along with significant loss of mitochondrial proteins when compared to WT cells (Fig. 1C,D). This loss of mitochondrial protein was rescued in the presence of Bafilomycin A1 (BafA1), a lysosomal inhibitor, indicating increased mitophagic flux in the FBXL4 KO cells. Consistent with the MLN4924 experiments, treatment of FBXL4 KO cells with MK-8722 restored mitochondrial proteins to a level comparable to WT cells, showing AMPK activation blocked FBXL4 KO-induced mitophagy. Importantly, MK-8722 treatment in FBXL4 KO cells did not decrease NIX levels but rather increased them (comparable to BafA1 treatment), indicating that AMPK activation blocks mitophagy independently of FBXL4-mediated regulation of NIX.

Previously, we have demonstrated that NIX is also a pexophagy receptor (Wilhelm et al, 2022) and shown, as with NIX-mediated mitophagy, that AMPK blocks NIX-mediated pexophagy (Longo et al, 2024). Given this, FBXL4 could also impact pexophagy; however, previous work found no evidence for this (Chen et al, 2023). In line with this, we did not observe any significant difference in the levels of the peroxisomal proteins, PMP70 and catalase, between WT and FBXL4 KO cells, in contrast to the significant reduction in mitochondrial COXIV protein (Fig. EV1B,C). This suggests loss of FBXL4 does not alter pexophagy, at least in ARPE-19 cells, and peroxisomal NIX levels may be regulated by a different mechanism.

To further support the negative effect of pharmacological AMPK activation on mitophagy in FBXL4 KO cells, we also tested two other structurally distinct allosteric AMPK activators: PXL-770 (Gluais-Dagorn et al, 2022) and BI-9774 (BI, 2017) (Fig. EV1D,E). Both compounds activated AMPK, as signified by increased substrate phosphorylation (ACC1 Ser80 and ULK1 Ser556). As with MK-8722, they also significantly increased the level of mitochondrial proteins in FBXL4 KO cells, indicating AMPK-mediated inhibition of mitophagy.

As an alternate way to measure mitophagy, we stably expressed the *mito-*QC reporter in WT and FBXL4 KO ARPE-19 cells. The reporter works on the principle of lysosomal quenching of GFP fluorescence from a tandem mCherry-GFP tag localized to the outer mitochondrial membrane (Allen et al, 2013). In the cytoplasm, mitochondria fluoresce both red and green, thus appearing yellow/green under the microscope. Upon mitophagy induction, the mitochondria are delivered to the lysosome, resulting in quenching of GFP signal and appearance of red-only punctate structures—indicating mitolysosomes. A significantly higher number of mitolysosomes were observed in FBXL4 KO cells compared to WT cells, with fewer mitolysosomes observed following MK-8722 treatment (Fig. 1E,F). The alternate AMPK activators, PXL-770 and BI-9774, also decreased mitolysosome numbers in FBXL4 KO cells (Fig. EV1F,G). Therefore, by two independent measures, we demonstrate that loss of FBXL4 enhances mitophagy, and this can be blocked by AMPK activation.

To confirm that the effects of MK-8722 are AMPK-dependent, we utilized BAY-3827, a potent AMPK-selective inhibitor (Fraguas Bringas et al, 2025; Lemos et al, 2021), in FBXL4 KO cells (Fig. EV1H,I). BAY-3827 alone, or in combination with MK-8722, reduced phospho-ACC1 and phospho-ULK1 levels, confirming AMPK inhibition. The combination treatment also abolished the MK-8722-induced increase in mitochondrial proteins. In line with this, BAY-3827 prevented MK-8722’s ability to block mitolysosome formation (Fig. EV1J,K). This indicates MK-8722 inhibits mitophagy and restores mitochondrial protein content in FBXL4 KO cells via AMPK activation.

### AMPK activation restores mitochondrial content and respiration deficiency

**Figure 2:**
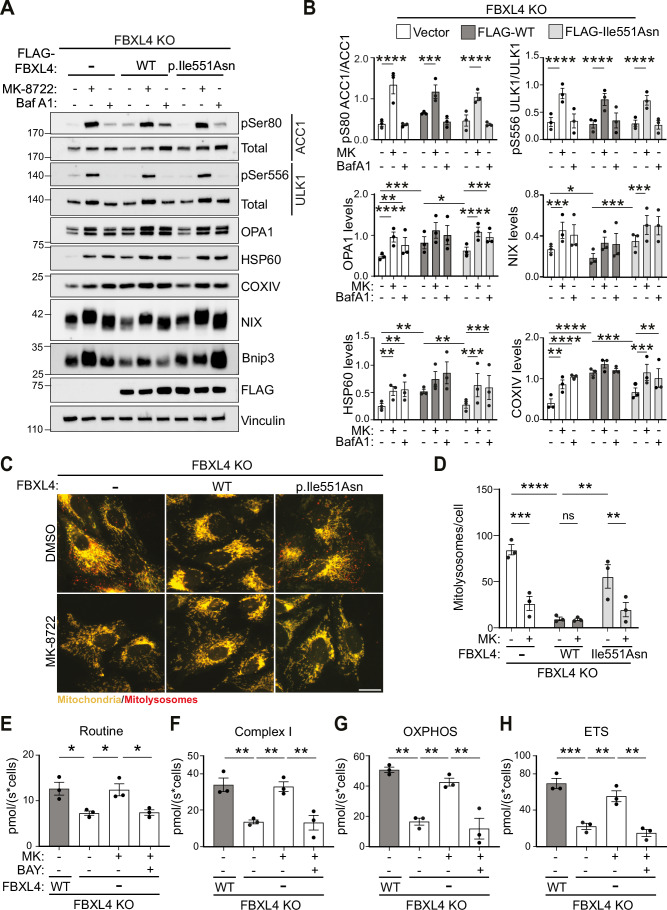
AMPK activation restores mitochondrial content and respiration deficiency. (**A**) Representative immunoblots of indicated proteins from FBXL4 knockout (FBXL4 KO) cells stably expressing either FLAG-empty vector (−), FLAG-FBXL4 wildtype (WT), or FLAG-FBXL4 mutant (p.Ile551Asn) treated with MK-8722 (10 μM) for 48 h or Bafilomycin A1(BafA1, 50 nM) for the final 24 h. (**B**) Quantification of the immunoblot data in panel A with protein levels normalized to loading controls. Data represents mean ± SEM (*n* = 3), significance calculated using two-way ANOVA and Tukey’s multiple comparisons test. (**C**) Representative wide-field images of FBXL4 KO *mito*-QC cells stably expressing either empty vector (−), FLAG-FBXL4 WT or FLAG-FBXL4 p.Ile551Asn treated with MK-8722 (10 μM) for 48 h. Red puncta represent mitolysosomes while mitochondrial network appears yellow. Scale bar: 20 μm. (**D**) Quantification of the number of mitolysosomes per cell. Data represents mean ± SEM (*n* = 3), significance calculated using two-way ANOVA and Tukey’s multiple comparison test. (**E**,** F**) Graph representing routine (**E**), Complex I (**F**), OXPHOS (**G**), and ETS (**H**) respiration measured from FBXL4 KO cells either expressing FLAG-empty vector or FLAG-FBXL4 WT treated with MK-8722 (10 μM) or BAY-3827 (5 μM) for 48 h by high-resolution respirometry. Oxygen flux is normalized to the cell number. Data represents mean ± SEM (*n* = 3), significance calculated using ordinary one-way ANOVA and Tukey’s multiple comparison test. For all the experiments, statistical significance is depicted as *p < 0.05, **p < 0.01, ***p < 0.001, ****p < 0.0001, and ns nonsignificant. Source data are available online for this figure.

Individuals with segregating, damaging FBXL4 variants harbor a significant reduction in mitochondrial content and associated mitochondrial respiratory deficiencies. If the mitochondrial respiration deficit is due to enhanced mitophagy, then blocking mitophagy should restore levels. To evaluate this, we first expressed either the FLAG-tagged WT or p.Ile551Asn mutant, a deleterious variant of FBXL4 that harbors a critical mutation in the C-terminal leucine-rich repeat region that impairs SCF-FBXL4 assembly (Cao et al, 2023; El-Hattab et al, 2017) in FBXL4 KO ARPE-19 cells and assessed the basal and MK-8722-induced level of mitochondrial proteins (Fig. 2A,B). Rescue of cells with WT-FBXL4 significantly reduced NIX levels in comparison to the FBXL4 KO (expressing empty FLAG vector) or the p.Ile551Asn mutant. A similar trend in BNIP3 levels was also observed, indicating FBXL4-mediated proteasomal regulation of NIX and BNIP3. Importantly, re-expression of WT, but not p.Ile551Asn mutant FBXL4, resulted in a significantly higher level of mitochondrial proteins OPA1, HSP60, and COXIV, confirming that loss of FBXL4 function is responsible for the observed increase in mitophagy. Consistent with previous results, treatment of both FBXL4 KO and p.Ile551Asn mutant cells with MK-8722 restored the level of evaluated mitochondrial proteins to basal WT levels. This level was comparable to BafA1 treatment, indicating AMPK activation inhibits mitophagic degradation of mitochondrial proteins. To further confirm the mitophagy levels, we stably expressed *mito*-QC in these cells (Fig. 2C,D). As with the immunoblot data, we observed a significant loss of mitolysosomes in FBXL4 KO *mito*-QC cells rescued with WT-FBXL4 compared to cells expressing empty vector or mutant FBXL4. Again, MK-8722 treatment significantly reduced mitolysosome number in both FBXL4 KO and p.Ile551Asn mutant cells. Therefore, MK-8722 treatment blocks NIX/BNIP3-mediated mitophagy and restores the levels of evaluated mitochondrial proteins in FBXL4 KO and I551N mutant cells.

**Figure EV2:**
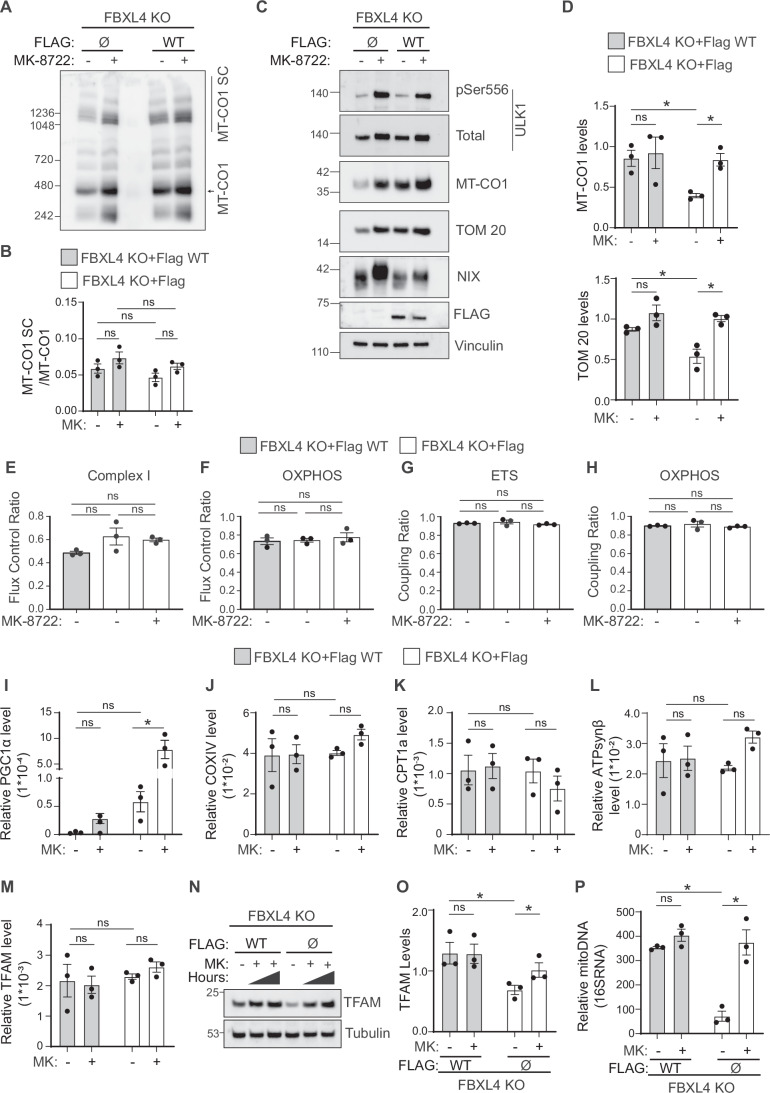
Effect of AMPK activation on mitochondrial respiration biogenesis. (**A**) Crude mitochondrial fraction from FBXL4 KO and WT rescue cells with/without 10 μM MK-8722 for 48 h was resolved in a Blue Native PAGE gel and immunoblotted with MT-CO1 antibody. (**B**) Quantification of the ratio of mitochondrial complex/supercomplex (upper bands) to MT-CO1 complex (lower band with arrowhead). Data represents mean ± SEM (*n* = 3), significance calculated using two-way ANOVA and uncorrected Fisher’s LSD. (**C**) Representative immunoblot of indicated protein from the whole cell lysate of FBXL4 KO and WT rescue cell with/without MK-8722 (10 μM) for 48 h. (**D**) Quantification of the immunoblot data in panel C with protein levels normalized to loading controls. Data represents mean ± SEM (*n* = 3), significance calculated using two-way ANOVA and Uncorrected Fisher’s LSD. (**E**,** F**) Graph representing Flux control ratio (FCR) of Complex I (**E**) and OXPHOS (**F**) states measured from FBXL4 KO cells either expressing FLAG-empty vector or FLAG-FBXL4 WT treated with MK-8722 (10 μM) for 48 h by high-resolution respirometry. FCR denotes the ratio of oxygen flux of Complex I and OXPHOS normalized to respective ETS states. Data represents mean ± SEM (*n* = 3), significance calculated using ordinary one-way ANOVA and Tukey’s multiple comparison test. (**G**,** H**) Graph representing coupling ratio (CR) of ETS (**G**) and OXPHOS (**H**) states measured from FBXL4 knockout cells either expressing FLAG-empty or FLAG-FBXL4 WT treated with MK-8722 (10 μM) for 48 h by high-resolution respirometry. CR denotes the ratio of net oxygen flux from ETS and OXPHOS normalized to respective ETS and OXPHOS states. Data represents mean ± SEM (*n* = 3), significance calculated using ordinary one-way ANOVA and Tukey’s multiple comparison test. (**I**–**M**) Relative expression of indicated genes relative to housekeeping *ACTIN* in FBXL4 KO and WT rescue cells with/without MK-8722(10 μM) for 48 h. Data represents mean ± SEM (*n* = 3), significance calculated using two-way ANOVA and Uncorrected Fisher’s LSD. (**N**) Representative immunoblot of TFAM and Tubulin of FBXL4 KO and WT rescue cell with/without MK-8722 (10 μM) for 48 and 96 h. (**O**) Quantification of the immunoblot data in panel (**N**) with protein levels normalized to loading controls. Data represents mean ± SEM (*n* = 3), significance calculated using two-way ANOVA and Uncorrected Fisher’s LSD. (**P**) Relative expression of mtDNA relative to housekeeping *ACTIN* in FBXL4 KO and WT rescue cells with/without MK-8722(10 μM) for 48 h. Data represents mean ± SEM (*n* = 3), significance calculated using two-way ANOVA and uncorrected Fisher’s LSD. For all the experiments, statistical significance is depicted as *p < 0.05, **p < 0.01, ***p < 0.001, ****p < 0.0001, and ns nonsignificant.

Next, we asked whether the mitochondria restored upon AMPK-mediated mitophagy inhibition are functional. We performed high-resolution respirometry in FBXL4 KO cells expressing either empty vector or WT-FBXL4 and measured respiration following stimulation with various mitochondrial complex substrates and specific inhibitors (Fig. 2E–H). Cells expressing the WT-FBXL4 had a significantly higher oxygen consumption rate compared to FBXL4 KO cells under all conditions tested: base rate (routine state, Fig. 2E), following addition of complex I substrate (CI, pyruvate-malate; ADP; glutamate, Fig. 2F), complex II substrate (OXPHOS and succinate, Fig. 2G) and maximal, uncoupled rate (ETS and CCCP, Fig. 2H) as explained in detail in the Methods. These data demonstrate a significant loss of mitochondrial respiration in FBXL4 KO cells. As AMPK activation can inhibit mitophagy and restore mitochondrial levels in FBXL4 KO cells, we reasoned that this would also restore mitochondrial respiration. Indeed, this proved to be the case as MK-8722 increased oxygen consumption across all conditions to a level comparable with WT cells (Fig. 2E–H). This restoration was dependent upon enhanced AMPK activity, as it was abolished by co-treatment with BAY-3827. This suggests that in FBXL4 KO cells, AMPK returns respiration levels to those of WT cells via blocking mitophagy and maintaining mitochondrial content. However, it is possible that other effects of AMPK activation may be mediating the restoration of mitochondrial respiration. For example, OPA1 levels increase in FBXL4 KO cells following AMPK activation, and OPA1 is a pro-survival protein that plays key roles in mitochondrial fusion, cristae structure, cell death and supercomplex assembly (Cipolat et al, 2004; Cogliati et al, 2013; Frezza et al, 2006; Varanita et al, 2015). To investigate this in more depth, we first evaluated whether AMPK activation alters assembly of the respiratory supercomplexes, which in turn can alter mitochondrial respiration. We subjected isolated mitochondria, from FBXL4 KO and WT rescue cells treated with MK-8722, to Blue Native PAGE (BN-PAGE). Mitochondrial complexes/supercomplexes were then detected by immunoblotting for MT-CO1 (Complex IV-subunit 1) (Fig. EV2A,B). We did not observe any significant changes in the ratio of lower molecular weight MT-CO1 complex bands to higher molecular weight mitochondrial supercomplex bands, indicating no significant alteration in supercomplex assembly. However, we did observe that FBXL4 KO cells appeared to have reduced MT-CO1 levels, which increased with MK-8722 treatment. This is consistent with the overall reduction of mitochondrial proteins observed in FBXL4 KO cells, which is restored upon AMPK activation. To confirm this, we evaluated the level of MT-CO1 and TOM20 (a mitochondrial outer membrane protein outside the respiratory complex) by conventional immunoblotting (Fig. EV2C,D). Indeed, we observed a significant and similar reduction of both MT-CO1 and TOM20 levels in FBXL4 KO cells, compared to WT rescue, and levels were restored upon MK-8722 treatment. Next, we measured the flux control ratio and coupling ratio, which is a valid method to normalize respiration to mitochondrial density and can be correlated with mitochondrial complex activity (Fig. EV2E–H). Overall, there were no significant differences between FBXL4 KO cells and those expressing WT-FBXL4, indicating that neither FBXL4 KO nor AMPK activation alters the intrinsic mitochondrial oxygen consumption rate under the conditions of the experiment. Taken together, these data suggest that the main consequence of FBXL4 loss is a reduction in mitochondrial quantity rather than quality.

As discussed, AMPK activation leads to mitochondrial biogenesis via upregulation of the transcriptional co-activator PGC1α. By interacting with other transcription factors, such as. ERRα and NRF1/2, PGC1α induces transcription of multiple nuclear encoded mitochondrial genes, including *TFAM*, which leads to mitochondrial DNA transcription (Huss et al, 2004; Schreiber et al, 2004; Wu et al, 1999). As pharmacological AMPK activation was shown to promote mitochondrial biogenesis in primary hepatocytes and mouse models (Fraguas Bringas et al, 2025; Narkar et al, 2008), we investigated this in FBXL4 KO cells. MK-8722 significantly increased *PGC1α* transcripts (~450-fold) in FBXL4 KO cells. An increasing trend (~17-fold) was also observed in the wild-type cells (Fig. EV2I). In contrast, MK-8722 treatment did not significantly alter the transcript levels of mitochondrial genes (*COXIV, CPT1a, ATPB*, and *TFAM*; <1.5-fold change) in either genotype (Fig. EV2J–M). Notably, a significant reduction in TFAM protein level was observed in FBXL4 KO cells, which, as with other proteins tested, was rescued in the presence of MK-8722 (Fig. EV2N,O). Given the lack of TFAM transcript changes, this indicates that AMPK activation rescues TFAM protein abundance by inhibiting mitophagy (Fig. EV2N,O). Since mitochondrial DNA (mtDNA) content can be correlated with mitochondrial mass in cells, we performed qPCR to evaluate the mtDNA content in FBXL4 KO and WT cells post MK-8722 treatment. FBXL4 KO cells showed a significant reduction in mtDNA compared to WT cells. This is in line with a previous report suggesting a reduction in mtDNA in FBXL4 mutant fibroblast (Bonnen et al, 2013; Emperador et al, 2019). MK-8722 treatment significantly increased mtDNA in the FBXL4 KO cells to levels similar to WT cells (Fig. EV2P). Together, the recovery of mitochondrial proteins across multiple compartments and the restoration of mtDNA levels indicate that AMPK activation increases mitochondrial content in FBXL4 KO cells. Given that only *PGC1α* transcripts were significantly upregulated, while other mitochondrial genes remained unchanged, this effect is likely driven, at least within the 48 h experimentally tested here, by reduced mitophagic degradation rather than increased biogenesis. Nevertheless, AMPK activation may stimulate mitochondrial biogenesis at later time points, as *PGC1α* induction represents an early step in the transcriptional cascade leading to mitochondrial expansion. Consistent with this, increases in *PGC1α* transcript levels precede elevations in mitochondrial proteins and mtDNA during exercise-induced mitochondrial biogenesis in human skeletal muscle (Perry et al, 2010; Pilegaard et al, 2003).

### AMPK activation restores mitophagy levels in patient-derived FBXL4 mutant fibroblasts

**Figure 3:**
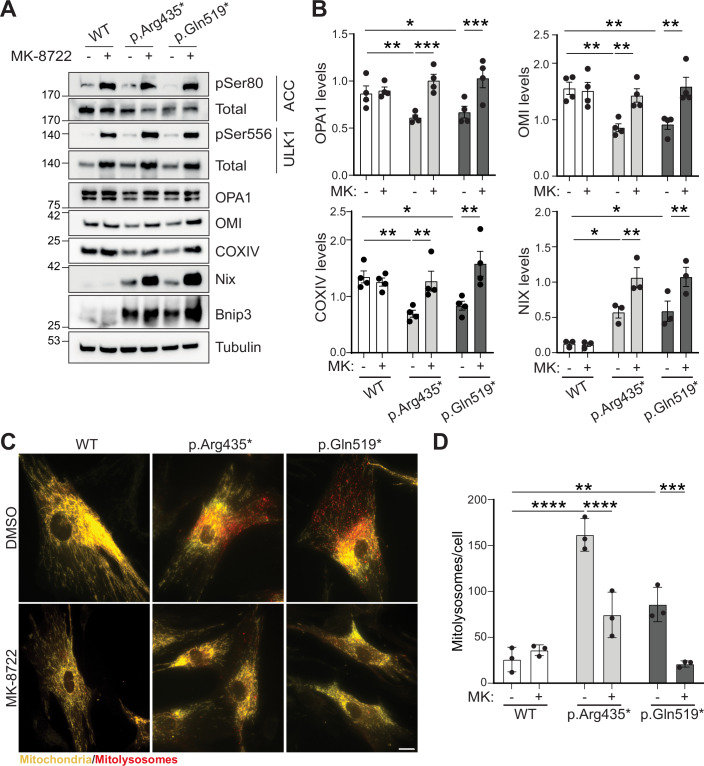
AMPK activation inhibits mitophagy in primary FBXL4 mutant patient fibroblasts. (**A**) Representative immunoblot of indicated proteins in normal (WT) and two FBXL4 mutant, p.Arg435* or p.Gln519*, human MTDPS13 patient fibroblast lines treated with MK-8722 (10 μM) for 48 h (**B**) Quantification of the immunoblot data in panel A with protein levels normalized to loading controls. Data represents mean ± SEM (*n* = 4), significance calculated using ordinary two-way ANOVA and Tukey’s multiple comparisons test. (**C**) Representative wide-field images of normal WT and p.Arg435* and p.Gln519* FBXL4 mutant human fibroblast lines stably expressing *mito*-QC sensor treated with MK-8722 (10 μM) for 48 h. Red puncta represent mitolysosomes while the mitochondrial network appears yellow. Scale bar: 20 μm. (**D**) Quantification of the number of mitolysosomes (red puncta) per cell. Data represents mean ± SEM (*n* = 3), significance calculated using ordinary two-way ANOVA and Sidak’s multiple comparison test. For all the experiments, statistical significance is depicted as *p < 0.05, **p < 0.01, ***p < 0.001, and ****p < 0.0001. Source data are available online for this figure.

To demonstrate disease relevance, we next studied the effect of MK-8722 in patient-derived primary fibroblast cell lines isolated from MTDPS13 patients, utilizing cells obtained from patients harboring homozygous, loss-of-function, truncating *FBXL4* variants. Cells with either the p.Arg435* or p.Gln519* (GenBank RefSeq NM_001278716.2) variants were reported to have significantly diminished levels of mitochondrial DNA and proteins (Bonnen et al, 2013). In line with these reports, we observed significantly reduced levels of mitochondrial proteins OPA1, OMI, and COXIV, along with a significant increase in NIX and BNIP3 levels, in both the mutant lines compared to WT-FBXL4-derived fibroblasts (Fig. 3A,B). Importantly, MK-8722 treatment restored the level of mitochondrial proteins to that of WT fibroblasts. Next, to more directly evaluate mitophagy, we stably expressed the *mito*-QC reporter in these cells (Fig. 3C,D). A significant increase in mitolysosomes was observed in the FBXL4 mutant fibroblast lines compared to WT cells. Consistent with the immunoblot data, MK-8722 treatment significantly reduced the mitolysosomes numbers back to the WT cell baseline. The data therefore shows that AMPK activation can reduce the level of NIX/BNIP3-mediated mitophagy and restore mitochondrial levels in FBXL4 mutant MTDPS13 patient-derived fibroblasts.

### AMPK activation reverses mitophagy defects in a chemical-based mouse model of MTDPS13

**Figure EV3:**
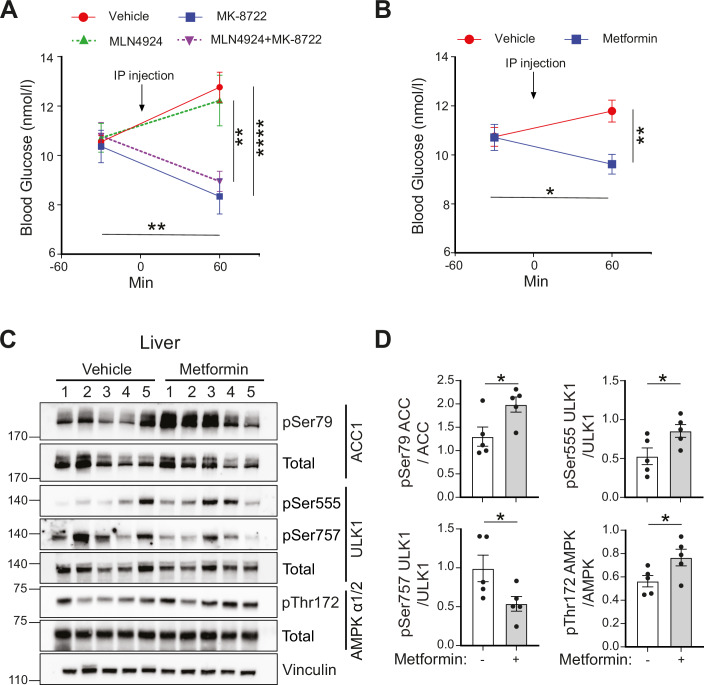
Effect of MK-8722 and Metformin on blood glucose levels and AMPK signaling. (**A**) Graph representing the blood glucose level of *mito*-QC mice monitored 30 min pre- and 60 min post day 1 interperitoneal injection of vehicle, MK-8722 (30 mg/kg), MLN4924 (50 mg/kg) by blood micro-sampling. Data represents mean ± SEM (*n* = 8 mice), significance calculated using two-way ANOVA and Tukey’s multiple comparisons test. (**B**) Graph representing blood glucose level of *mito*-QC mice monitored 30 min pre- and 60 min post day 1 interperitoneal injection of vehicle or Metformin (150 mg/kg) by blood micro-sampling. Data represents mean ± SEM (*n* = 10 mice), significance calculated using two-way ANOVA and uncorrected Fisher’s LSD test. (**C**) Representative immunoblots of indicated proteins from liver lysates from *mito*-QC mice injected with vehicle or metformin (150 mg/kg) for 5 days. (**D**) Quantification of the immunoblot data in panel (**C**). Data represents mean ± SEM (*n* = 5), significance calculated using an unpaired *t*-test. For all the experiments, statistical significance is depicted as *p < 0.05, **p < 0.01, ***p < 0.001, and ****p < 0.0001.

While we have shown that AMPK activation can rescue FBXL4 mitophagy defects in cell culture models, these systems do not represent true physiological conditions, necessitating further experimental evidence to justify AMPK activation as a potential therapeutic approach to treat MTDPS13. To address this, we used transgenic *mito*-QC mice, which constitutively expresses the *mito*-QC reporter enabling visualization of mitochondrial architecture and mitophagy within various tissues (McWilliams et al, 2016). Using this model, we have previously shown that MK-8722 administration reduced basal mitophagy in multiple tissues (Longo et al, 2024), suggesting this represents a viable option for treatment. Unfortunately, constitutive KO of FBXL4 in mice results in perinatal lethality, with most animals dying within 3 days of birth (Alsina et al, 2020; Cao et al, 2023). Given that conditional FBXL4 KO mice are not yet available, we opted for a pharmacological approach to mimic the disease by inhibiting FBXL4 with MLN4924 (though in this instance, we cannot rule out that inhibition of other CRLs, such as VHL, may contribute to any observed phenotypes). Previously, an MLN4924 dose of 60 mg/kg in the mouse had been reported to reduce Cullin Neddylation in a human tumor xenograft model (Soucy et al, 2009). Thus, we administered the similar dose to *mito*-QC mice, either alone or in combination with MK-8722, daily for five consecutive days. Animals were sacrificed at the end of the dosing period on day 5, and tissues were harvested and processed for biochemical and mitophagy assays. AMPK activation results in a sustained lowering of blood glucose levels (Myers et al, 2017; Rhein et al, 2021), and to confirm this occurred with MK-8722 administration, we measured blood glucose levels pre and post each MK-8722 treatment (Fig. EV3A).

**Figure 4:**
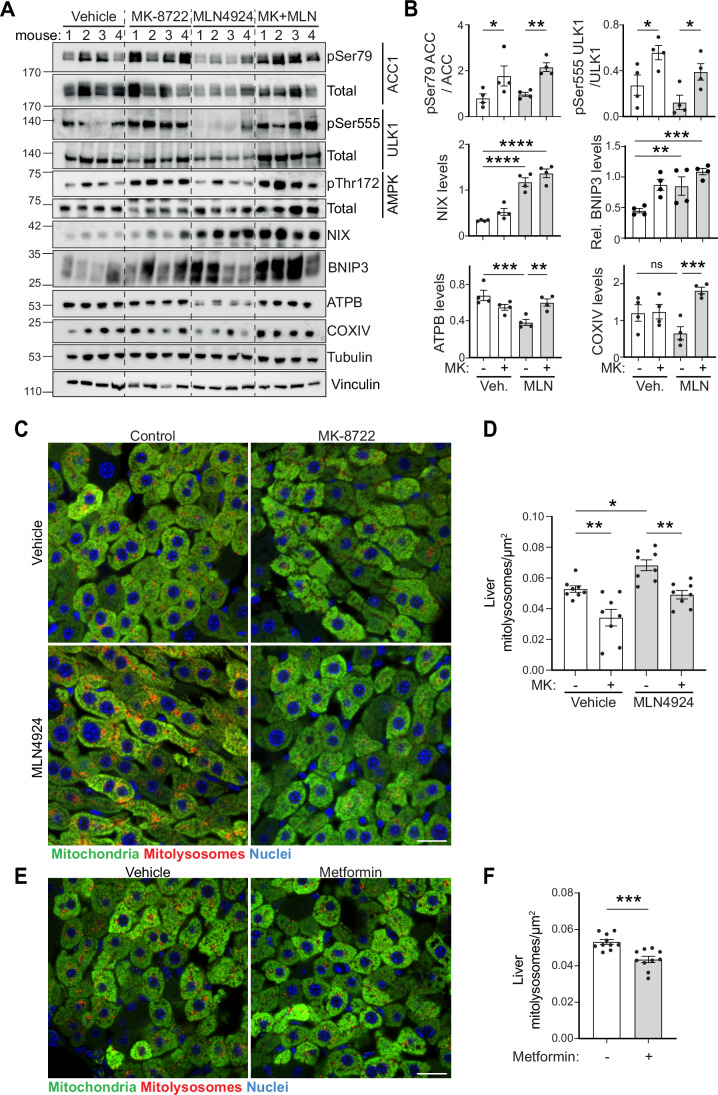
AMPK activation inhibits basal and MLN4924-induced mitophagy in the liver. (**A**) Representative immunoblots of indicated proteins from liver lysates from *mito*-QC mice dosed with vehicle, MK-8722 (30 mg/kg) and MLN4924 (MLN, 50 mg/kg) for 5 days. Each lane represents a different mouse. (**B**) Quantification of the immunoblot data in panel (**A**) with protein levels normalized to loading controls. Data represents mean ± SEM (*n* = 4), significance calculated using two-way Anova with uncorrected Fisher’s LSD. (**C**) Representative confocal micrographs of the liver sections from *mito*-QC mice dosed with vehicle, MK-8722 (30 mg/kg) and MLN4924 (MLN, 50 mg/kg) for 5 days. Red puncta indicate mitolysosomes, whereas the mitochondrial network is depicted in green. Scale bar: 20 μm. (**D**) Quantification of the number of mitolysosomes normalized to tissue area. Data represents mean ± SEM (*n* = 8 mice), significance calculated using ordinary one-way ANOVA and Sidak’s multiple comparisons test. (**E**) Representative confocal micrographs of liver sections from *mito*-QC mice dosed with vehicle or metformin (150 mg/kg) for 5 days. Red puncta indicate mitolysosomes, whereas the mitochondrial network is depicted in green. Scale bar: 20 μm. (**F**) Quantification of the number of mitolysosomes normalized to tissue area. Data represents mean ± SEM (*n* = 10), significance calculated using an unpaired *t*-test. For all the experiments, statistical significance is depicted as *p < 0.05, **p < 0.01, ***p < 0.001, ****p < 0.0001. Source data are available online for this figure.

As AMPK is a crucial regulator of hepatic metabolic processes and 33% of the reported FBXL4-related MTDPS13 cases show hepatopathy (Almannai et al, 2017), we focused on the analysis of liver tissue. Immunoblotting of liver lysates confirmed that MK-8722 administration, either alone or in combination with MLN4924, significantly increased AMPK activity as monitored by phosphorylation of AMPKα at Thr172, ACC1 at Ser79, and ULK1 at Ser555 (Fig. 4A,B), which is consistent with the glucose-lowering effect of MK-8722 (Fig. EV3A). To evaluate whether MLN4924 administration recapitulates FBXL4-mediated NIX and BNIP3 regulation, we monitored the levels of NIX, BNIP3, and the mitochondrial proteins ATPB and COXIV. A significant increase in the levels of both NIX and BNIP3 was observed in the MLN4924-treated group compared to the vehicle. Further, MLN4924 alone significantly lowered ATPB levels (with a similar trend for COXIV protein levels), indicating activation of NIX/BNIP3-mediated mitophagy. Co-administration of MK-8722 with MLN4924 significantly impaired the MLN4924-induced reduction in mitochondrial proteins, indicating mitophagy inhibition. To confirm that MLN4924 is indeed inducing mitophagy and that it is impacted by AMPK activation, we analysed the *mito*-QC reporter in liver sections (Fig. 4C,D). Firstly, MLN4924 treatment alone significantly increased mitolysosome numbers in the liver, confirming that this compound induces mitophagy. In line with our previous work (Longo et al, 2024), MK-8722 treatment alone reduced liver mitolysosome numbers, demonstrating that AMPK activation reduces overall mitophagy in the liver. Importantly, and consistent with the immunoblot data, MK-8722 administration prevented MLN4924-induced mitolysosome formation in mouse liver.

Given the effect on liver mitophagy, as well as the therapeutic potential, we also analysed the effects of metformin, the first-line treatment of type 2 diabetes, on liver mitophagy. Since repurposing drugs is both time and cost-effective, and metformin is one of the most prescribed drugs that indirectly activates AMPK (Rena et al, 2013), we reasoned that this analysis could provide valuable insights. *Mito*-QC mice were treated daily with metformin for 5 days at a dose of 150 mg/kg (Fullerton et al, 2013), and tissues were then harvested and processed for biochemical and mitophagy assays. Consistent with AMPK activation, metformin administration significantly lowered blood glucose levels (Fig. EV3B) and increased substrate phosphorylation (ACC1 at Ser79 and ULK1 at Ser555), as measured by immunoblotting of liver lysates (Fig. EV3C,D). To monitor mitophagy, we quantified *mito*-QC in liver sections (Fig. 4E,F). Metformin treatment led to a significant reduction in the number of mitolysosomes, indicating that metformin-mediated AMPK activation also reduces basal mitophagy in the liver. However, the extent of this reduction was less pronounced than that observed with MK-8722, likely reflecting metformin´s lower potency and indirect mechanism of AMPK activation. Taken together, our data show that multiple AMPK activators can modulate liver mitophagy, acting through the BNIP3/NIX-mediated pathway in a negative manner. Considering that metformin primarily targets the liver and exhibits a weaker inhibitory effect on mitophagy compared to MK-8722, we focused on assessing the effects of MK-8722 in other tissues.

**Figure EV4:**
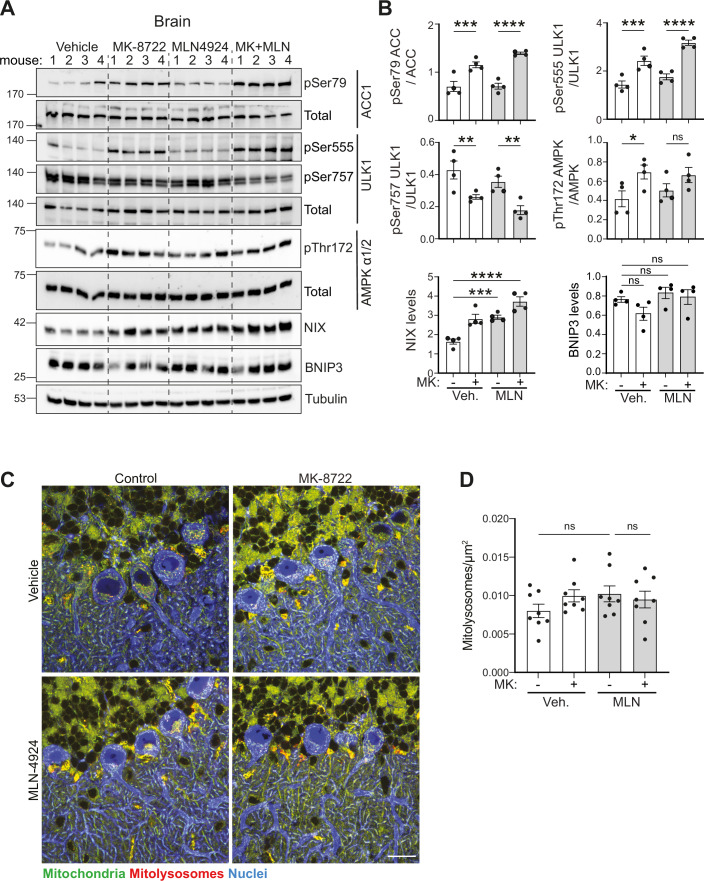
Effect of MK-8722 on AMPK signaling and mitophagy in the brain. (**A**) Representative immunoblots of indicated proteins from whole brain lysates from *mito*-QC mice dosed with vehicle, MK-8722 (30 mg/kg) and MLN4924 (MLN, 50 mg/kg) for 5 days. Each lane represents an individual mouse. (**B**) Quantification of the immunoblot data in panel (**A**) with protein levels normalized to loading controls. Data represents mean ± SEM (*n* = 4 mice), significance calculated using ordinary two-way ANOVA with Uncorrected Fisher’s LSD test. (**C**) Representative confocal micrographs of Calbindin immunolabelled Purkinje neurons (Blue) in the Cerebellum from the brain sections of *mito*-QC mice dosed with vehicle, MK-8722 (30 mg/kg) and MLN4924 (MLN, 50 mg/kg) for 5 days. Red puncta indicate mitolysosomes, whereas the mitochondrial network is depicted in green. Scale bar: 20 μm. (**D**) Quantification of the number of mitolysosomes normalized to calbindin-labeled Purkinje neuron area. Data represents mean ± SEM (*n* = 8), significance calculated using ordinary one-way ANOVA and Sidak’s multiple comparisons test. For all the experiments, statistical significance is depicted as *p < 0.05, **p < 0.01, ***p < 0.001, ****p < 0.0001, and ns nonsignificant.

**Figure 5:**
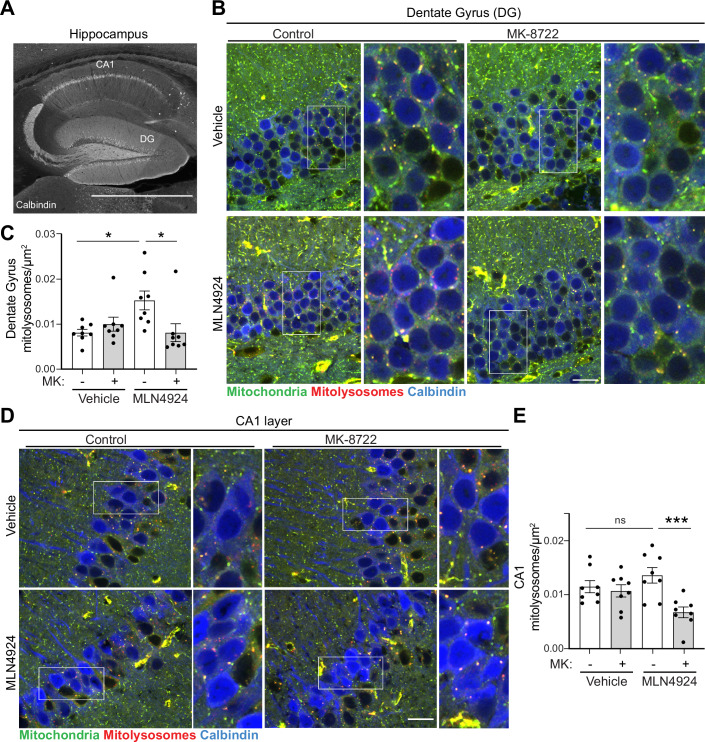
AMPK activation inhibits MLN4924-induced mitophagy in the hippocampus. (**A**) Representative tile scan confocal micrograph of the mouse hippocampus depicting calbindin-positive (Gray) neurons in the molecular layer of Dentate Gyrus (DG) and the Cornu Ammonis (CA1) regions. Scale bar, 5 mm. (**B**) Representative confocal micrograph of Calbindin immunolabelled neurons (blue) in the molecular layer of dentate gyrus (DG) and (**D**) cornu ammonis (CA) layer from the brain sections of *mito*-QC mice dosed with vehicle, MK-8722 (30 mg/kg) and MLN4924 (MLN, 50 mg/kg) for 5 days. Enlarged images of the boxed region are shown on the right of the respective images. Red puncta indicate mitolysosomes, whereas the mitochondrial network is depicted in green. Scale bar: 20 μm. (**C**) Quantification of the number of mitolysosomes normalized to the area of the calbindin-labeled DG neurons. Data represents mean ± SEM (*n* = 8), significance calculated using ordinary one-way ANOVA and Sidak’s multiple comparisons test. (**D**, **E**) as for panels (**B**, **C**) but monitoring calbindin-labeled neurons in the CA1 region. For all the experiments, statistical significance is depicted as *p < 0.05, ***p < 0.001, and ns nonsignificant. Source data are available online for this figure.

Patients with segregating, pathogenic FBXL4 variants often show severe neurological abnormalities, implying that the homeostasis of NIX/BNIP3-mediated mitophagy in the brain is critical (Almannai et al, 2017). We therefore investigated this further in the brains of animals treated with MLN4924 with/without MK-8722. A significant increase in NIX levels was observed in total brain tissue lysate from the animals receiving MLN4924, suggesting inhibition of FBXL4 (Fig. EV4A,B). However, there was no significant change in BNIP3 levels, which could suggest NIX is more susceptible to the effects of MLN4924 in the brain. Animals receiving MK-8722, either alone or in combination with MLN4924, showed the expected increase in phosphorylation of AMPK substrates (ACC1 Ser79 and ULK1 Ser555) as well as the activating AMPK Thr172 phosphorylation, indicating activation of the AMPK signaling pathway in the brain. We also observed increased NIX levels in the MK-8722 group, which increased even further in combination with MLN4924, indicating AMPK activation may modulate brain mitophagy. To evaluate this, we first focused on the hippocampus, the brain center responsible for memory consolidation, spatial navigation, and decision-making (Attaallah et al, 2024; Bird and Burgess, 2008; Eichenbaum, 2017). We analysed mitophagy in calbindin-positive neurons of the molecular layer of Dentate Gyrus (DG) and Cornu Ammonis (CA1) region (Fig. 5A). MLN4924 significantly increased the number of mitolysosomes in the neurons of the DG compared to the vehicle group, indicating activation of NIX-mediated mitophagy. MK-8722 alone did not alter the number of mitolysosomes (Fig. 5B,C). However, the combination of MK-8722 with MLN4924 significantly reduced the number of mitolysosomes to a level comparable to the vehicle group. An increasing trend in the mitolysosome number following MLN4924 treatment was also observed in the neurons of the CA1 layer, which again was significantly reduced to basal levels in animals co-treated with MK-8722 (Fig. 5D,E). Similarly, MK-8722 alone did not alter mitophagy in the neurons of the CA1 layer. This indicates inhibition of the SCF family of proteins, including FBXL4, increases NIX levels and mitophagy in hippocampal neurons, and AMPK activation prevents this. We note, unlike liver, that MK-8722 alone did not alter basal mitophagy in the hippocampal neurons. This could relate to the contribution of different mitophagy pathways within these neurons and the fact that the *mito*-QC assay cannot distinguish between them. We have previously shown that AMPK activation can have contrasting roles in regulating different mitophagy pathways, for example, it activates the PINK1/Parkin pathway (Longo et al, 2024), which may be more active in these neurons. Potentially related to this, not all neuronal populations displayed a mitophagy change upon MLN4924 and/or MK-8722 administration. For example, we did not observe any significant changes in the number of mitolysosomes in the Purkinje neurons of the Cerebellum (Fig. EV4C,D). This, however, could also relate to the relative neuron-specific activation of AMPK, and further work will be needed to determine this. Importantly, though, we show that FBXL4-dysregulated mitophagy is occurring within neuronal populations within the brain, and this can be rescued by administration of an AMPK activator. Given that the brain is a major organ affected in MTDPS13, we believe small-molecule AMPK activation is a promising therapeutic approach to treat this disorder.

## Discussion

FBXL4-related MTDPS13 is a rare and often fatal mitochondrial disorder for which no therapeutic interventions currently exist. To date, more than 100 individuals carrying pathogenic *FBXL4* variants have been reported worldwide, supporting detailed clinical phenotyping (Gao et al, 2024). Affected individuals exhibit severe clinical manifestations, including growth retardation, feeding difficulties, hypotonia, and profound developmental delay, with a median age of death of approximately 3 years (El-Hattab et al, 2017). Disease management, sadly, remains purely supportive.

Here, we identify pharmacological AMPK activation as a potential therapeutic strategy for MTDPS13. We demonstrate that AMPK activation suppresses excessive NIX/BNIP3-mediated mitophagy, thereby restoring mitochondrial content and improving mitochondrial respiratory function in FBXL4 KO, as well as in patient-derived fibroblasts harboring FBXL4 loss-of-function truncating variants (Figs. 2, 3 and EV2). We have previously shown that distinct mitophagy pathways selectively target different mitochondrial subpopulations. Specifically, mitochondria eliminated via the NIX-mediated pathway retain higher membrane polarization and respiratory capacity than those degraded by the PINK1/Parkin pathway following mitochondrial damage (Longo et al, 2024). The current findings further support this preferential degradation of functional mitochondria by NIX/BNIP3-mediated mitophagy, as stabilization of NIX/BNIP3 in FBXL4 KO cells triggered excessive mitophagy even in the absence of mitochondrial stressors. If the mitochondria targeted by NIX/BNIP3 were already severely compromised, their preservation would not have enhanced the mitochondrial respiration levels observed in FBXL4 KO cells (Fig. 2E–H). Together, these data underscore the role of NIX/BNIP3-mediated mitophagy in regulating mitochondrial abundance and cellular metabolism. A severe reduction in mitochondrial respiration, caused by uncontrolled NIX/BNIP3-dependent mitophagy, is likely responsible for the energetic and metabolic failure observed in multiple tissues of MTDPS13 patients. Consistent with this model, AMPK activation enhanced mitochondrial respiration in FBXL4 KO cells by restoring mitochondrial content without altering the assembly of mitochondrial supercomplexes and the intrinsic respiratory capacity of individual mitochondria (Fig. EV2A–H). Sustained AMPK activation is also known to stimulate mitochondrial biogenesis through PGC1α (Jäger et al, 2007; Zong et al, 2002). We observed an increase in *PGC1α* transcript levels in FBXL4 KO cells following MK-8722 treatment. Consistent with this, MK-8722 has been shown to specifically activate AMPK and induce *PGC1α* transcription, an effect that is abolished in AMPKα1/2 double knockout cells or upon treatment with the AMPK inhibitor BAY-3827 (Fraguas Bringas et al, 2025). Despite the robust increase in *PGC1α* transcripts, we did not observe a corresponding change in the transcript levels of all the evaluated mitochondrial proteins. We hypothesize that an increase in *PGC1α* reflects the early initiation of mitochondrial biogenesis, which may translate into increased mitochondrial content only after prolonged AMPK activation. This is consistent with studies using AMP-mimetic pro-drug 5-aminoimidazole-4-carboxamide-1-β-D-ribofuranoside (AICAR), where sustained AMPK activation promotes mitochondrial biogenesis in rodent skeletal muscle in vivo (Narkar et al, 2008; Winder et al, 2000). Together, these findings suggest that pharmacological activation of AMPK could offer a dual therapeutic benefit in MTDPS13 by both suppressing excessive mitophagy and promoting mitochondrial biogenesis.

Beyond the context of FBXL4-related mitochondrial disease, our findings refine the prevailing view of AMPK as a universal activator of autophagy and mitophagy. While nutrient deprivation typically activates AMPK to induce autophagic degradation of cellular components for ATP generation, mitochondria themselves are the primary sites of ATP production. Thus, indiscriminate degradation of functional mitochondria would be energetically disadvantageous. We previously demonstrated that AMPK phosphorylates ULK1 at Ser556 and Ser694, facilitating its interaction with 14-3-3 proteins. This interaction sequesters ULK1 and prevents it from phosphorylating downstream targets such as ATG13 and ATG14, thereby inhibiting initiation of NIX-dependent mitophagy (Longo et al, 2024). In agreement with this mechanism, AMPK activation markedly reduced mitolysosome formation in FBXL4 KO/mutant cell lines and patient-derived fibroblasts (Figs. 2C,D and 3C,D), confirming that AMPK blocks the initiation phase of NIX/BNIP3-mediated mitophagy.

Interestingly, PPTC7, a positive regulator of the FBXL4–SCF complex, is upregulated during starvation, serving to prevent mitophagy activation and protect mitochondria under energy stress (Sun et al, 2024). Proteomic analyses of autophagic cargoes likewise reveal that mitochondria are largely excluded from degradation during nutrient deprivation (Hickey et al, 2023). Our findings uncover an additional layer of control by which AMPK prevents the degradation of functional mitochondria via inhibition of NIX/BNIP3-dependent mitophagy. In contrast, AMPK promotes the clearance of damaged mitochondria through the PINK1-Parkin pathway independently of ULK1 (Longo et al, 2024). Together, these observations highlight a dual and context-dependent role for AMPK in mitochondrial quality control—facilitating removal of damaged organelles while preserving healthy ones.

Crucially, we now demonstrate that AMPK activation inhibits excessive mitophagy in an in vivo chemical-based model of MTDPS13 (Figs. 4 and 5). Consistent with the neurological features of FBXL4 deficiency, we observed elevated mitophagy in the hippocampus following MLN4924 administration. Activation of AMPK by MK-8722 suppressed MLN4924-induced mitophagy without affecting basal turnover (Fig. 5B–E). Although the blood–brain barrier permeability of MK-8722 was not reported (Feng et al, 2018; Myers et al, 2017) and remains to be fully established, our findings suggest that systemic AMPK activation is sufficient to modulate neuronal mitophagy.

Drug repurposing offers a rapid and cost-effective route to therapeutic development. Although MK-8722 is a preclinical compound, our findings strongly support the potential of AMPK activators in FBXL4-related MTDPS13. We additionally tested metformin, an orally bioavailable indirect AMPK activator widely prescribed for treatment of type 2 diabetes and found that it reduced basal mitophagy in the liver (Fig. 4E,F). However, metformin’s pharmacokinetic profile may limit its systemic utility: it is primarily absorbed in the liver and intestine via organic cation transporter (OCT) 1 and, to a lesser extent, in the kidney via OCT3 (Rena et al, 2013; Shu et al, 2007). Moreover, high doses can cause lactic acidosis, restricting its application in mitochondrial disorders such as MTDPS13 that frequently associate with a metabolic acidosis (Salpeter et al, 2010).

Of further note, PXL-770, a direct allosteric AMPK activator tested in this study, has advanced to phase IIa clinical trial and demonstrated favorable tolerability in patients with non-alcoholic fatty liver disease (Cusi et al, 2021). PXL-770 has been shown to improve mitochondrial function in models of X-linked adrenoleukodystrophy (ALD) and autosomal dominant polycystic kidney disease (ADPKD), and to restore mitochondrial DNA content in kidneys of PKD1 KO (Dagorn et al, 2023; Monternier et al, 2022). In our hands, PXL-770 activated AMPK to a comparable extent as MK-8722 (Fig. EV1D,E) and was equally effective in suppressing mitophagy and restoring mitochondrial content in FBXL4 KO cells (Fig. EV1F,G). These data identify PXL-770 and other clinically applicable small-molecule AMPK activators, as potential candidates for therapeutic repurposing in FBXL4-related MTDPS13.

In summary, our study highlights the inhibitory role of AMPK in the mitophagic degradation of functional mitochondria and provides a mechanistic rationale for targeting AMPK in FBXL4-related MTDPS13. By suppressing NIX/BNIP3-mediated mitophagy and promoting mitochondrial biogenesis, AMPK activation represents a promising strategy to restore mitochondrial homeostasis in this otherwise untreatable disease. These findings warrant clinical evaluation of existing AMPK activators for safety and efficacy, and further development of next-generation direct AMPK activators as potential therapies for mitochondrial DNA depletion syndromes.

## Methods

**Table:** Reagents and tools table

Reagent/resource	Reference or source	Identifier or catalog number
**Experimental models**		
*mito-*QC mice	McWilliams et al J Cell Biol (2016); (Mice generated by TaconicArtemis GmbH)	N/A
*ARPE-19*	ATCC	Cat#CRL-2302; RRID:CVCL_0145
FBXL4 WT fibroblasts	Bonnen et al, Am J Hum Genet (2013)	N/A
FBXL4 p,Arg435* fibroblasts		
FBXL4 p.Gln519* fibroblasts		
**Recombinant DNA**		
pBABE *mito-*QC retroviral system	MRC PPU Reagents and Services, University of Dundee	Cat#DU40799
pBABE-FBXL4 FLAG3 (I551N) retroviral system	MRC PPU Reagents and Services, University of Dundee	Cat#71999
pBABE-FBXL4 WT FLAG3 retroviral system	MRC PPU Reagents and Services, University of Dundee	Cat#71998
**Antibodies**		
Phospho Ser79 ACC	Cell Signaling Technology	Cat: #3661; RRID:AB_330337
ACC	Cell Signaling Technology	Cat# 3662; RRID:AB_2219400
Phospho Ser555 ULK1	Cell Signaling Technology	Cat# 5869; RRID:AB_10707365
Phospho Ser757 ULK1	Cell Signaling Technology	Cat# 6888; RRID:AB_10829226
ULK1	Cell Signaling Technology	Cat# 8054; RRID:AB_11178668
TOM20	Santa Cruz Biotechnology	Cat# sc-17764 RRID:AB_628381
OMI	MRC PPU Reagents and Services, University of Dundee	Cat# S802C
HSP60	Cell Signaling Technology	Cat# 4870; RRID:AB_2295614
TFAM	Abcam	Cat# ab119684; RRID:AB_10900340
NIX	Cell Signaling Technology	Cat# 12396; RRID:AB_2688036
BNIP3	Cell Signaling Technology	Cat# 44060; RRID:AB_2799259
MT-CO1	Cell Signaling Technology	Cat# 55159 T RRID: AB_3713023
OPA1	BD Biosciences	Cat# 612606; RRID:AB_399888
ATPB	Abcam	Cat# ab14730; RRID:AB_301438
COXIV	Cell Signaling Technology	Cat# 4850; RRID:AB_2085424
Phospho Thr172 AMPK	Cell Signaling Technology	Cat# 2535; RRID:AB_331250
AMPK pan-α mouse	Cell Signaling Technology	Cat# 2793; RRID:AB_915794
Catalase	Millipore	Cat#219010-1 ML RRID:AB_211660
PMP70	Sigma-Aldrich	Cat# P0497, RRID:AB_477306
GAPDH	Proteintech	Cat# 60004-1-Ig; RRID:AB_2107436
Vinculin	Abcam	Cat# ab129002; RRID:AB_11144129
FLAG	Sigma-Aldrich	Cat# F1804, RRID:AB_262044)
Tubulin	Proteintech	Cat# CL647-66031, RRID:AB_2920274
Calbindin	Swant	Cat# cb38a, RRID:AB_3107026
Goat anti-Rabbit IgG (H + L) Secondary Antibody HRP	Thermo Fisher Scientific	Cat# 31460; RRID:AB_228341
Goat anti-Mouse IgG (H + L) Secondary Antibody HRP	Thermo Fisher Scientific	Cat# 31430; RRID:AB_228307
Donkey anti-Sheep IgG (H + L) Secondary Antibody HRP	Thermo Fisher Scientific	Cat# A16041; RRID:AB_2534715
Goat anti-Rabbit IgG (H + L) Secondary Antibody, Pacific Blue	Thermo Fisher Scientific	Cat# P-10994, RRID:AB_2539814
**Oligonucleotides and other sequence-based reagents**		
PCR primers	This Study	Method: Quantitative PCR
FBXL4 KO Guides	This Study	Method: Generation of CRISPR cell lines
NIX KO Guides	This Study	Method: Generation of CRISPR cell lines
BNIP3 KO Guides	This Study	Method: Generation of CRISPR cell lines
Random primers	Promega	Cat#C1181
**Chemicals, enzymes and other reagents**		
DMEM-F12	Gibco	Cat#
DMEM	Gibco	Cat#
EcoRI	Thermo Fisher	Cat#FD0274
Bafilomycin A1	Enzo	Cat#BML-CM110
MK-8722	Selleck Chemicals	Cat #E1031
MLN4924	Merck	Cat#5054770001
MLN4924	MedChemExpress	Cat#HY-70062
Bay-3827	MRC PPU Reagents and Services, University of Dundee	Cat# Bay-3827
Lipofectamine 2000	Thermo Fisher	Cat #11668019
X-treme Gene HD	Roche	Cat ##6366244001
cOmplete™, EDTA-free Protease Inhibitor Cocktail	Roche	Cat #11873580001
MES running buffer	Formedium	Cat#MES-SDS5000
MOPS running buffer	Formedium	Cat#MOPS-SDS1000
Protran® Nitrocellulose membrane	Amersham™	Cat#15259794
Clarity Western ECL Substrate	Bio-Rad	Cat#1705061
1× Native PAGE buffer	Thermo Fisher	Cat#BN2003
Digitonin	Thermo Fisher	#BN2006
Benzonase	Merck	Cat#E1014
G-250	Thermo Fisher	Cat#BN2004
a 3-14% native gel	Thermo Fisher	Cat#BN1001
Immobilon-P Membrane PVDF membrane	Merck	Cat#IPVH00010
PFA	Sigma-Aldrich	Cat#P6148
Prolong Gold Antifade Mountant with DAPI	Invitrogen	Cat# P36935
Prolong Glass Antifade Mountant	Invitrogen	Cat#P36984
OCT	Agar Scientific	Cat#AGR1180
Glass Slides	Leica	Cat#3800202
High-precision cover glasses	Marienfeld	Cat#0107222
Vectashield Antifade Mounting Medium	Vector Laboratories	Cat# H-1000
Hoechst 33342	Thermo Fisher	Cat#H1399
Miro5	Oroboros	Cat#60101-01
Digitonin	Sigma-Aldrich	Cat#D5628
Pyruvate	Sigma-Aldrich	Cat#P2256
Malate	Sigma-Aldrich	Cat#M1000
ADP	Merck	Cat#117105-1GM
Glutamate	Sigma-Aldrich:	Cat#G1626
cytochrome c	Sigma-Aldrich	Cat#C7752
Succinate	Sigma-Aldrich	Cat#S2378
Rotenone	Sigma-Aldrich	Cat#R8875
Antimycin	Sigma-Aldrich:	Cat#A8674
SuperScript III reverse transcriptase	Thermo Fisher	Cat#18080044
PowerUp™ SYBR™ Green Master Mix	Thermo Fisher	Cat#A25742
β-cyclodextrin	Sigma-Aldrich	Cat#H5784
Methylcellulose	Sigma-Aldrich	Cat #M0512
polysorbate 80	Sigma-Aldrich	Cat#P4780-100ML
Sodium lauryl sulfate	Sigma-Aldrich	Cat#75746-250GM
**Software**		
mito-QC counter	ImageJ plugin	https://github.com/graemeball/mQC_counter
Volocity	PerkinElmer	http://cellularimaging.perkinelmer.com/downloads/
FlowJo	BD Biosciences	https://www.flowjo.com/solutions/flowjo
Prism Version 10.1.0.316	GraphPad Software	https://www.graphpad.com/features
**Other**		
Zeiss LSM 880 Confocal Airyscan Microscope	Zeiss	LSM 880
Leica Cryostat	Leica	CM1860UV
QIAprep Spin Miniprep Kit	Qiagen	Cat##27104
genomic DNA isolation kit	Qiagen	Cat#69504
BCA assay kit	Thermo Fisher Scientific	Cat #23227
Cellcrusher Tissue pulverizer	Cellcrusher, Cork, Ireland	NA
Flow cytometer with DIVA software	BD Biosciences	BD LSRFortessa II
High-resolution respirometer	Oroboros instruments, Innsbruck, Austria	Oxygraph-2k

### Antibodies and reagents

Primary antibodies were used at a dilution of 1:1000 for immunoblotting unless mentioned otherwise: Phospho Ser79 ACC (Cell Signaling Technology (CST) #3661), ACC (CST #3662), Phospho Ser556 ULK1 (CST #5869S), Phospho Ser757 ULK1 (CST #6888S), ULK1 (CST #8054S), OMI (MRC PPU Reagents and Services, University of Dundee, S802C), HSP60 (CST #4870), NIX (CST #12396), OPA1 (BD Bioscience #612606), ATPB (Abcam #ab14730), COXIV (Cat# 4850) AMPK pan-α (CST # 2532S, # 2793S), AMPK pan-β (CST # 4150S), Phospho Thr172 AMPKα (CST #2535S), Anti-FLAG (Sigma #F1804) Tubulin (Proteintech #66031-1-Ig, 1:10,000), Vinculin (Abcam #ab129002, 1:10,000), GAPDH (Proteintech #60004-1-Ig, 1:10,000), Calbindin-D28k (Swant, #CB38,1:500).

Secondary antibodies were obtained from Thermo Fisher and used at a dilution of 1:10,000 for immunoblotting: goat anti-Rabbit IgG-HRP (#31460), goat anti-mouse IgG-HRP (#31430), donkey anti-sheep IgG-HRP (#A16041), and Pacific Blue Goat anti-Rabbit IgG (Invitrogen #P10994, 1:200)

Bafilomycin A1 (BML-CM110) was procured from Enzo, MK-8722 (#HY-111363) and PXL-770 (#HY-160004) were brought from MedChemExpress, BI-9774 was kindly provided by Boehringer Ingelheim via its open innovation platform opnMe, available at https://opnme.com, MLN4924 (#5054770001) was purchased from Merck.

### Cell culture, transfection, and infection

ARPE-19 cells and Patient fibroblast lines were cultured in DMEM: F12 media supplemented with 10% fetal bovine serum, 2 mM L-glutamine, 100 U/ml Penicillin, and 0.1 mg/ml Streptomycin. HEK293/FT cells were cultured in DMEM, supplemented with 10% (v/v) FBS, 2 mM L-glutamine, 100 U/ml Penicillin, and 0.1 mg/ml Streptomycin. All cell lines were grown at 37 °C with 5% CO_2_ in a humidified incubator. Fibroblasts from affected individuals and age-matched controls were obtained as previously described by informed consent in accordance with the Declaration of Helsinki protocols and local Institutional Review Boards (Bonnen et al, 2013).

Retroviral transduction was used to generate stable cells expressing the protein of interest. For generating the retrovirus, 70% confluent HEK293FT cells were transfected with 0.5 µg of pBABE vector expressing respective genes, 0.33 µg of Gag-Pol, and 0.17 µg of VSVG using X-treme Gene HD (Roche #6366244001) according to the manufacturer’s protocol. The cell supernatant was harvested after 48 h of transfection and filtered through a 0.45 μm filter to remove floating cells. The filtered supernatant was used to transduce cells at 70% confluency with polybrene (10 μg/ml). After 48 h of transduction, the cells were selected with either puromycin (2 μg/ml) or hygromycin (100 μg/ml) and the stable pool of cells was used for the experiment.

### Generation of CRISPR cell lines

NIX knockout cells were generated using Cas9 D10A nuclease and paired guide RNAs (gRNAs) targeting exon 2 of the *NIX* gene (gRNA sense: GTTGTGGATGGAGGATGAGGA, gRNA anti-sense: GAGACATGGAGAAGATTCTTT) (Wilhelm et al, 2022). BNIP3 knockout cells were generated using a single guide TCTTGTGGTGTCTGCGAGCG cloned into the pX459 Cas9 vector. For FBXL4 knockout, two single CRISPR guides, cloned in the pX459 Cas9 vector, were tested individually. Guide 1: GTTTAGCAGTGCTGTCCTCG and Guide 2: GTTGGTCAGAGAGACCTACGA. Guides were transfected in ARPE-19 cells with 6 µl of GeneJuice (Sigma #70967) per µg of DNA. Post 24 h of transfection, 2 µg/ml of puromycin was added for 24 h for selecting transfected cells. The cells were then allowed to recover. Single-cell clones were generated by serial dilution and screened using immunoblotting for NIX, as no validated FBXL4 antibody is commercially available. Guide 2 successfully led to an increase in NIX levels.

To confirm knockout of FBXL4, genomic DNA was extracted using the Qiagen DNeasy kit following the manufacturer’s instructions. The CRISPR cleavage site was PCR amplified using KOD Hot start PCR (Merck #71086) according to the manufacturer’s protocol with the following primers (FBXL4: Fwd: GTTGGTTTAAGCCATGCACACAGG, Rev: AGCATGTATTGCC CACAGTTAAAGTG; BNIP3: Fwd: GAGGAAGAGTTTGGCTCTGGCAGG, Rev: CGGTGTATCCCTGATGGCAG). The PCR product was then cloned into a holding plasmid using the StrataClone Blunt PCR cloning kit (Agilent #240207) and transformed into the StrataClone *E.* *coli* cells provided in the kit. Plasmids were isolated from multiple bacterial clones using QIAprep Spin Miniprep Kit (Qiagen #27104) following the manufacturer’s protocol. Positive clones containing the PCR product were identified with EcoRI digestion (Thermo Fisher, #FD0274). The positive clones were then analysed with Sanger sequencing. FBXL4 KO harbored a homozygous single-nucleotide deletion causing a frameshift mutation and the introduction of multiple premature stop codons beginning at the 183rd codon. The single BNIP3 KO cells contained a single-nucleotide insertion leading to a frameshift mutation and the generation of a stop codon at the 94th codon. While two large insertions leading to the generation of two stop codons at the 94th and 181st codons of BNIP3 were observed in NIX /BNIP3 DKO cells, which already harbored a premature stop codon at the 51st codon of NIX.

### Plasmids

pBABE mito-QC retroviral system (DU40799) was used as described previously (Allen et al, 2013). pBABED-Puro-FBXL4-FLAG2 (DU71998), pBABED-Puro-FBXL4-I551N-FLAG2 (DU71999) retroviral constructs, as well as mito-QC, can be obtained from MRC PPU reagents and Services.

### Immunoblotting

Cell lysates were prepared using NP-40 lysis buffer (50 mM HEPES, pH 7.4, 150 mM NaCl, 1 mM EDTA, 10% glycerol, 1% NP-40) supplemented with phosphatase inhibitor cocktail (1.15 mM sodium molybdate, 4 mM sodium tartrate dihydrate, 10 mM β-glycerophosphoric acid disodium salt pentahydrate, 1 mM sodium fluoride, 1 mM activated sodium orthovanadate), and with protease inhibitor cocktail (Roche # 11873580001). Protein concentration in the lysates was determined by BCA assay (Thermo Fisher, #23227). Equal amounts of proteins were resolved in 14–6% Bis-Tris gels by gel electrophoresis at 110–150 V for 90 min using 1x Bis-Tris Running Buffer. The resolved proteins were transferred into Amersham™ Protran® nitrocellulose (0.45 µm, #15259794) membrane and blocked with Milk (5% in TBST). Membranes were then incubated overnight at 4 °C with respective primary antibodies. On the following day, membranes were washed three times with TBST and incubated with respective secondary HRP antibodies for 1 h at room temperature. Membranes were then washed, and signals were detected using ECL (Bio-Rad, Clarity Western ECL Substrate, # 1705061) in the Bio-Rad Chemidoc imager as per the manufacturer’s protocol.

For tissue immunoblotting, respective snap frozen tissue samples were individually homogenized using Cellcrusher (Cellcrusher, Cork, Ireland) tissue pulverizer. The homogenized tissues were weighed and incubated in 10X volume of RIPA buffer (50 mM Tris-HCl, pH 8, 150 mM NaCl, 1 mM EDTA, 1% NP-40, 1% Na-deoxycholate, and 0.1% SDS) supplemented with protease inhibitor cocktail and phosphatase inhibitor cocktail for 30 min on ice. At 15-min intervals, tissue was pulverized for better extraction of proteins. Lysates were clarified by centrifugation at 14,000 rpm for 15 min at 4 ˚C. Protein concentration was determined using the BCA assay (Thermo Fisher #23227). An equal amount of protein was resolved in a 6–14% Bis-Tris gel, and immunoblotting was performed as mentioned above.

### Blue native PAGE (BN-PAGE)

BN-PAGE was performed as described previously to detect mitochondrial complexes/supercomplexes by immunoblotting with an MT-CO1 antibody (Raimi et al, 2024). Briefly, FBXL4 KO and WT cells were harvested in PBS post 48 h of treatment. Cells were then disrupted with a 25-gauge needle. Following clarification of the nuclear and cell membrane pellet at 800×*g* for 5 min, the mitochondrial fraction was enriched by 17000×*g* for 20 min at 4 °C. For BN-PAGE analysis, the mitochondria-enriched pellet was processed in 1× Native PAGE buffer (Thermo Fisher #BN2003) supplemented with 1% digitonin (Thermo Fisher #BN2006), 1 U/μl Benzonase (Merck #E1014) and 2 mM MgCl_2_. Mitochondrial lysate was then clarified by centrifugation at 20,000×*g* for 30 min at 4 °C. Protein concentrations were determined by BCA assay (Thermo Fisher #23227). Equal amounts of proteins from each sample were then supplemented with 0.002% G-250 (Thermo Fisher #BN2004) and resolved in a 3-14% native gel (Thermo Fisher #BN1001). Proteins were transferred onto a PVDF membrane. The excess G-250 stain was washed with 100% methanol and subjected to immunoblotting with MT-CO1 as described above.

### Microscopy-based *mito*-QC assay in cell lines

Cells stably expressing the *mito*-QC sensor were generated by retroviral transduction. The stable cells were seeded on a coverslip and incubated overnight in standard culture conditions. The following day, cells were treated with the respective drug as mentioned above for 48 h. At the end of treatment, cells were washed three times with PBS and fixed at room temperature for 10 min in 3.7% paraformaldehyde (PFA) buffered with 200 m HEPES, pH 7.0. The fixed cells were washed and incubated for 15 min at RT with DMEM-HEPES (10 mM, pH 7.0) containing 0.02% NaN3 to quench PFA. At the end of incubation, coverslips containing the cells were washed twice with PBS and mounted using Prolong Glass Antifade Mountant (Invitrogen, # P36984) and imaged on a Nikon Eclipse Ti2 wide-field microscope with a 63X objective. Quantitation of the number of red-only puncta, indicating mitolysosomes, was performed using a semiautomated *mito*-QC counter in ImageJ software as described previously (Montava-Garriga et al, 2020).

### Flow cytometric-based *mito*-QC assay in cell lines

mito-QC cells were trypsinized following treatment and fixed for 15 min in 3.7% PFA prepared in 200 mM HEPES (pH 7.0). After fixation, cells were washed twice with PBS and resuspended in 0.5 ml of Dulbecco’s PBS supplemented with 2% FBS. Data from 10,000 cells were acquired using a BD LSRFortessa II flow cytometer with DIVA software (BD Biosciences). EGFP was excited with a 488 nm laser and detected using a 530/30 emission filter, whereas mCherry was excited with a 561 nm laser and detected with a 610/20 emission filter. Live cells were gated based on forward and side scatter profiles, and singlets were selected. Mitophagy was quantified as the EGFP/mCherry fluorescence ratio. Gates were initially defined using untreated (DMSO) cells by selecting the top 5% of cells with high mitophagy, and these parameters were subsequently applied to all other conditions. Data were analysed using FlowJo software version 10.7.1 (BD Biosciences).

### High-resolution respirometry

FBXL4 KO cells stably expressing FLAG-empty vector or FLAG-FBXL4 were treated with either MK-8722 alone or in combination with BAY-3827 for 48 h. Cells were then trypsinized and counted. About 1 × 10^6^ cells were resuspended in 100 μl of Miro5 (Oroboros, #60101-01) mitochondrial respiration buffer. Samples were then loaded into the thermostated oxygraphic chamber at 37 °C with continuous stirring (Oxygraph-2k, Oroboros instruments, Innsbruck, Austria) to measure mitochondrial respiration. Cells were then permeabilized with digitonin (10 μg/10^6^ cells), and mitochondrial respiration for different respiration states was measured using the Substrate-Uncoupler-Inhibitor titration protocol number 8 (SUIT-008) as per the Oroboros protocol. Briefly, routine or leak respiration (PMLN) was first measured in the absence of any mitochondrial substrates and ATP. Mitochondrial respiration from Complex I (PGMp) was then measured by sequential injection of pyruvate (5 mM), malate (2 mM), ADP (2.5 mM), and glutamate (10 mM). Mitochondrial membrane integrity (PMcp) was tested by adding cytochrome c (10 μM) between the ADP and Glutamate injection steps. Any samples showing more than 10% increase in oxygen flux post cytochrome c addition was discarded as they indicate mitochondrial membrane damage. Combined contribution of Complex I and II (PGMSp, OXPHOS) on mitochondrial respiration was then measured with the injection of Succinate (10 mM). Uncoupled respiration (PGMSE, ETS) was next measured with CCCP titration. Residual mitochondrial respiration (ROX) was then measured by sequential addition of Rotenone (0.5 μM) and antimycin (2.5 μM). The ROX oxygen flux value was subtracted from each of the above mitochondrial respiration states to quantify mitochondrial respiration of each state. Results are expressed in pmol/(s*cells).

### Quantitative PCR

Quantitative PCR was performed to evaluate the level of mitochondrial biogenesis and mtDNA in FBXL4 KO and WT cells post MK-8722 treatment.

To determine the expression levels of genes associated with mitochondrial biogenesis, RNA was isolated from cells following MK-8722/DMSO treatment using the RNA isolation kit (Qiagen #74104) following the manufacturer’s instructions. About 1 μg of RNA was then converted to CDNA using Random primers (Promega #C1181) using the SuperScript III reverse transcriptase (Thermo Fisher #18080044) following the manufacturer’s instructions. About 10 ng of CDNA was used for quantitative PCR using PowerUp™ SYBR™ Green Master Mix (Thermo Fisher #A25742) in Bio-Rad ThermoCycler following standard qPCR cycle at melting temperature of 60 °C. The expression (ΔCt) level of each gene was determined using gene-specific primers (as listed below) compared to ACTIN expression.

For quantitation of mtDNA, genomic DNA was isolated from cells following MK-8722/DMSO treatment using a genomic DNA isolation kit (Qiagen #69504) following the manufacturer’s instructions. About 10 ng of genomic DNA was then directly used for quantitative PCR using PowerUp™ SYBR™ Green Master Mix (Thermo Fisher #A25742) in Bio-Rad ThermoCycler following standard qPCR cycle at melting temperature of 60 °C. The level (ΔCt) of mtDNA was determined using gene-specific primers against 16S rRNA (listed below) and compared to the ACTIN level in genomic DNA.

*PGC1α* Fwd: AGCCTCTTTGCCCAGATCTT, Rev: GGCAATCCGTCTTCATCCAC,

NT- PGC1α Fwd: TCACACCAAACCCACAGAGA, Rev: CTGGAAGATATGGCACAT,

COX4i1 Fwd: CAAGCGAGCAATTTCCACCT, Rev: GGTCACGCCGATCCATATAAG

TFAM Fwd: GATGCTTATAGGGCGGAGTGG, Rev: GCTGAACGAGGTCTTTTTGGT

CPT1A Fwd: TCCAGTTGGCTTATCGTGGTG, Rev: TCCAGAGTCCGATTGATTTTTGC 16S rRNA Fwd: GCCTTCCCCCGTAAATGATA, Rev: TTATGCGATTACCGGGCTCT, Actin: Fwd: ATCCGCCGCCCGTCCACA, Rev: ACCATCACGCCCTGGTGCCT,

### Animal studies

All animal breeding and experimental procedures were approved by the University of Dundee Ethical Review Committee, the Named Veterinary Surgeon, and the Compliance Officer. All work was conducted under a UK Home Office project license in accordance with the Animals (Scientific Procedures) Act 1986.

Two independent animal studies were performed. For both, mice were bred in-house and housed in sex-matched groups of two to five animals under a 12:12 h light–dark cycle at an environmental temperature of 21 ± 1 °C with 55–65% relative humidity. Food and water were provided *ad libitum*. Approximately 4-month-old male and female *mito*-QC mice on a C57BL/6J background were used for all experiments (McWilliams et al, 2016).

#### Study 1: SCF inhibition and AMPK activation

This study investigated the effects of SCF inhibition (MLN4924), AMPK activation (MK-8722), and combined treatment on mitophagy across multiple tissues. A total of 32 animals (*n* = 8 per group) were used, based on power calculations from our published in vivo data (Longo et al, 2024). Animals of both sexes were randomly assigned to groups and received once-daily intraperitoneal injections for 5 consecutive days. The vehicle for MLN4924 consisted of 5% DMSO and 20% (w/v) β-cyclodextrin (Sigma #H5784) in saline, and it was administered at a dose of 50 mg/kg (MedChem Express #HY-70062), adapted from Soucy et al (2009). MK-8722 was injected at a dose of 30 mg/kg based on (Myers et al, 2017). MK-8722 was formulated in 0.25% (w/v) methylcellulose, 5% (v/v) polysorbate 80, and 0.02% (w/v) sodium lauryl sulfate in sterile water.

#### Study 2: Metformin treatment

This study assessed the effects of metformin on hepatic mitophagy. A total of 20 animals (*n* = 10 per group) were included. Animals received once-daily intraperitoneal injections of either saline (vehicle control) or metformin at 150 mg/kg, dissolved in saline, for 5 consecutive days based on (Fullerton et al, 2013).

### Blood glucose measurement

Blood glucose levels were assessed by blood micro-sampling in all animals at two time points: 30 min prior to and 60 min after the first intraperitoneal injection with vehicle or compounds mentioned above. For micro-sampling, the tail was punctured using a 25-gauge needle, and small blood droplets were collected directly onto glucose test strips. Glucose levels were quantified with a digital glucometer (CONTOUR® Meter).

### Tissue collection and processing

Animals were anesthetized by intraperitoneal injection of 20% (v/v) pentobarbital (Euthatal, Merial) in PBS after 4 h of the final intraperitoneal injection of respective compounds and vehicle. To eliminate blood from tissues, animals were transcardially perfused with PBS, after which various organs were collected and divided into two parts. One part was immediately fixed in 3.7% paraformaldehyde (PFA) prepared in 200 mM HEPES buffer (Sigma, #P6148; pH 7.0) overnight at 4 °C. The other portion was flash-frozen in liquid nitrogen for subsequent biochemical analyses by immunoblotting as mentioned above. Fixed tissues were washed three times with PBS containing 0.1% Azide and stored in 30% (w/v) sucrose in PBS for cryopreservation until use. Liver tissues were embedded in OCT compound (Agar Scientific, #AGR1180) and cryosectioned (10 µm) using a Leica CM1860UV cryostat. The sections were mounted on glass slides (Leica, #3800202) and air-dried in a fume hood. Sections were washed three times with PBS and counterstained with Hoechst 33258 (1 µg/ml, 5 min, room temperature). Excess stain was removed by two additional PBS washes, and slides were mounted with Vectashield Antifade Medium (Vector Laboratories, H-1000) and sealed with high-precision cover glasses (Marienfeld, #0107222) using transparent nail polish.

### Immunolabeling of brain free-floating sections

Sagittal brain sections of 50-µm thickness were generated using a sledge microtome (Leica SM2010R), and slices were stored in PBS containing 0.1% Azide at 4 °C until further processing. Free-floating sections were permeabilized three times for 5 min in DPBS (Gibco, #14190094) containing 0.3% Triton X-100 (Sigma-Aldrich, #T8787). Samples were then incubated for 1 h in blocking buffer composed of DPBS supplemented with 10% goat serum (Sigma-Aldrich, G9023) and 0.3% Triton X-100. The sections were then stained with anti-calbindin-D28k (Swant, #CB38, 1:1000 in blocking buffer) by overnight incubation of free-floating sections at room temperature. On the following day, sections were washed twice (8 min each) in DPBS with 0.3% Triton X-100 and incubated for 1 h in blocking buffer containing Pacific Blue anti-rabbit IgG secondary antibody (Invitrogen P10994, 1:200). After two additional 8-min washes in DPBS with 0.3% Triton X-100, sections were mounted on adhesive slides (Leica, #3800202) with coverslip using Vectashield Antifade Mounting Medium (Vector Laboratories, H-1000) and sealed with transparent nail polish.

### Tissue confocal microscopy and quantitation of mitophagy in vivo

Tissue sections were imaged using an LSM 880 Airyscan microscope with Plan-Apochromat 63x/1.4 Oil DIC M27 objective. Approximately ten images or 80% of the tissue area (for Hippocampus) were imaged across two to three sections for each tissue sample per individual animal. Quantification of the number of mitolysosomes was performed using Volocity® software (version 6.3, PerkinElmer) as described previously (Singh et al, 2021). In brief, all channels were processed using the software’s default fine filtering to reduce background noise. For liver analysis, EGFP fluorescence was thresholded to define the tissue area, while for brain sections, Pacific Blue fluorescence was thresholded to define the neuronal area. Mitolysosomes were identified by detecting peaks in the mCherry/EGFP ratio channel. The false-positive signals in areas of low reporter expression were excluded by applying an additional threshold for mCherry intensity. Threshold values were kept constant for all the tissues within a given experimental set. Mitophagy was quantified as the mean number of mitolysosomes per unit tissue area (µm²).

### Statistical analysis

GraphPad Prism software (Version 10.1.0.316) was used for the statistical analysis. All data were represented as mean ± SEM. The number of biological replicates for each experiment is mentioned in the figure legends. Various statistical tests used to determine significance between groups are mentioned in the figure legend. The exact *P* values are listed in the Appendix Table S1.

## Supplementary information

Peer Review File

Source data Fig. 1

Source data Fig. 2

Source data Fig. 3

Source data Fig. 4

Source data Fig. 5

Appendix Table S1

Expanded View Figures

## Data Availability

This study includes no data deposited in external repositories. The source data of this paper are collected in the following database record: biostudies:S-SCDT-10_1038-S44321-026-00471-z.

## References

[CR1] Allen GF, Toth R, James J, Ganley IG (2013) Loss of iron triggers PINK1/Parkin-independent mitophagy. EMBO Rep 14:1127–1135 24176932 10.1038/embor.2013.168 PMC3981094

[CR2] Almannai M, Dai H, El-Hattab AW, Wong L-JC (2017) FBXL4-related encephalomyopathic mitochondrial DNA depletion syndrome. In: GeneReviews*®* [Internet]. University of Washington 28383868

[CR3] Alsina D, Lytovchenko O, Schab A, Atanassov I, Schober FA, Jiang M, Koolmeister C, Wedell A, Taylor RW, Wredenberg A et al (2020) FBXL4 deficiency increases mitochondrial removal by autophagy. EMBO Mol Med 12:e11659 32525278 10.15252/emmm.201911659 PMC7338799

[CR4] Antoun G, McBride S, Vanstone JR, Naas T, Michaud J, Redpath S, McMillan HJ, Brophy J, Daoud H, Chakraborty P et al (2016) Detailed biochemical and bioenergetic characterization of FBXL4-related encephalomyopathic mitochondrial DNA depletion. JIMD Rep 27:1–9 26404457 10.1007/8904_2015_491 PMC5580732

[CR5] Attaallah B, Petitet P, Zambellas R, Toniolo S, Maio MR, Ganse-Dumrath A, Irani SR, Manohar SG, Husain M (2024) The role of the human hippocampus in decision-making under uncertainty. Nat Hum Behav 8:1366–1382 38684870 10.1038/s41562-024-01855-2 PMC11272595

[CR6] Ballout RA, Al Alam C, Bonnen PE, Huemer M, El-Hattab AW, Shbarou R (2019) FBXL4-related mitochondrial DNA depletion syndrome 13 (MTDPS13): a case report with a comprehensive mutation review. Front Genet 10:39 30804983 10.3389/fgene.2019.00039 PMC6370620

[CR7] BI, 2017. opnMe.com - Boehringer Ingelheim Open Innovation Portal. opnMe.com Boehringer Ingelheim.

[CR8] Bird CM, Burgess N (2008) The hippocampus and memory: insights from spatial processing. Nat Rev Neurosci 9:182–194 18270514 10.1038/nrn2335

[CR9] Bonnen PE, Yarham JW, Besse A, Wu P, Faqeih EA, Al-Asmari AM, Saleh MA, Eyaid W, Hadeel A, He L et al (2013) Mutations in FBXL4 cause mitochondrial encephalopathy and a disorder of mitochondrial DNA maintenance. Am J Hum Genet 93:471–481 23993193 10.1016/j.ajhg.2013.07.017 PMC3769921

[CR10] Cao Y, Zheng J, Wan H, Sun Y, Fu S, Liu S, He B, Cai G, Cao Y, Huang H et al (2023) A mitochondrial SCF-FBXL4 ubiquitin E3 ligase complex degrades BNIP3 and NIX to restrain mitophagy and prevent mitochondrial disease. EMBO J 42:e113033 36896912 10.15252/embj.2022113033 PMC10308365

[CR11] Chen Y, Jiao D, Liu Y, Xu X, Wang Y, Luo X, Saiyin H, Li Y, Gao K, Chen Y et al (2023) FBXL4 mutations cause excessive mitophagy via BNIP3/BNIP3L accumulation leading to mitochondrial DNA depletion syndrome. Cell Death Differ 30:2351–2363 37568009 10.1038/s41418-023-01205-1 PMC10589232

[CR12] Cipolat S, Martins de Brito O, Dal Zilio B, Scorrano L (2004) OPA1 requires mitofusin 1 to promote mitochondrial fusion. Proc Natl Acad Sci USA 101:15927–15932 15509649 10.1073/pnas.0407043101 PMC528769

[CR13] Cogliati S, Frezza C, Soriano ME, Varanita T, Quintana-Cabrera R, Corrado M, Cipolat S, Costa V, Casarin A, Gomes LC et al (2013) Mitochondrial cristae shape determines respiratory chain supercomplexes assembly and respiratory efficiency. Cell 155:160–171 24055366 10.1016/j.cell.2013.08.032 PMC3790458

[CR14] Cusi K, Alkhouri N, Harrison SA, Fouqueray P, Moller DE, Hallakou-Bozec S, Bolze S, Grouin J-M, Jeannin Megnien S, Dubourg J et al (2021) Efficacy and safety of PXL770, a direct AMP kinase activator, for the treatment of non-alcoholic fatty liver disease (STAMP-NAFLD): a randomised, double-blind, placebo-controlled, phase 2a study. Lancet Gastroenterol Hepatol 6:889–902 34560015 10.1016/S2468-1253(21)00300-9

[CR15] Dagorn PG, Buchholz B, Kraus A, Batchuluun B, Bange H, Blockken L, Steinberg GR, Moller DE, Hallakou-Bozec S (2023) A novel direct adenosine monophosphate kinase activator ameliorates disease progression in preclinical models of autosomal dominant polycystic kidney disease. Kidney Int 103:917–929 36804411 10.1016/j.kint.2023.01.026

[CR16] Eichenbaum H (2017) The role of the hippocampus in navigation is memory. J Neurophysiol 117:1785–1796 28148640 10.1152/jn.00005.2017 PMC5384971

[CR17] Elcocks H, Brazel AJ, McCarron KR, Kaulich M, Husnjak K, Mortiboys H, Clague MJ, Urbe S (2023) FBXL4 ubiquitin ligase deficiency promotes mitophagy by elevating NIX levels. EMBO J 42:e112799 37102372 10.15252/embj.2022112799 PMC10308357

[CR18] El-Hattab AW, Dai H, Almannai M, Wang J, Faqeih EA, Al Asmari A, Saleh MAM, Elamin MAO, Alfadhel M, Alkuraya FS et al (2017) Molecular and clinical spectra of FBXL4 deficiency. Hum Mutat 38:1649–1659 28940506 10.1002/humu.23341

[CR19] Emperador S, Garrido-Perez N, Amezcua-Gil J, Gaudo P, Andres-Sanz JA, Yubero D, Fernandez-Marmiesse A, O’Callaghan MM, Ortigoza-Escobar JD, Iriondo M et al (2019) Molecular characterization of new FBXL4 mutations in patients with mtDNA depletion syndrome. Front Genet 10:1300 31969900 10.3389/fgene.2019.01300 PMC6960396

[CR20] Esteban-Martinez L, Sierra-Filardi E, McGreal RS, Salazar-Roa M, Marino G, Seco E, Durand S, Enot D, Grana O, Malumbres M et al (2017) Programmed mitophagy is essential for the glycolytic switch during cell differentiation. EMBO J 36:1688–1706 28465321 10.15252/embj.201695916 PMC5470043

[CR21] Feng D, Biftu T, Romero FA, Kekec A, Dropinski J, Kassick A, Xu S, Kurtz MM, Gollapudi A, Shao Q et al (2018) Discovery of MK-8722: a systemic, direct pan-activator of AMP-activated protein kinase. ACS Med Chem Lett 9:39–44 29348809 10.1021/acsmedchemlett.7b00417 PMC5767883

[CR22] Fraguas, Bringas C, Ahangar MS, Cuenco J, Liu H, Addinsall AB, Lindahl M, Ovens AJ, Febbraio MA, Foretz M, Goransson O et al (2025) Mechanism and cellular actions of the potent AMPK inhibitor BAY-3827. Sci Adv 11:eadx2434 40845097 10.1126/sciadv.adx2434 PMC12372887

[CR23] Frezza C, Cipolat S, Martins de Brito O, Micaroni M, Beznoussenko GV, Rudka T, Bartoli D, Polishuck RS, Danial NN, De Strooper B et al (2006) OPA1 controls apoptotic cristae remodeling independently from mitochondrial fusion. Cell 126:177–189 16839885 10.1016/j.cell.2006.06.025

[CR24] Fullerton MD, Galic S, Marcinko K, Sikkema S, Pulinilkunnil T, Chen ZP, O’Neill HM, Ford RJ, Palanivel R, O’Brien M et al (2013) Single phosphorylation sites in Acc1 and Acc2 regulate lipid homeostasis and the insulin-sensitizing effects of metformin. Nat Med 19:1649–1654 24185692 10.1038/nm.3372 PMC4965268

[CR25] Gai X, Ghezzi D, Johnson MA, Biagosch CA, Shamseldin HE, Haack TB, Reyes A, Tsukikawa M, Sheldon CA, Srinivasan S et al (2013) Mutations in FBXL4, encoding a mitochondrial protein, cause early-onset mitochondrial encephalomyopathy. Am J Hum Genet 93:482–495 23993194 10.1016/j.ajhg.2013.07.016 PMC3769923

[CR26] Ganley IG, Simonsen A (2022) Diversity of mitophagy pathways at a glance. J Cell Sci 135:jcs259748 36504076 10.1242/jcs.259748 PMC10656428

[CR27] Gao K, Xu X, Wang C (2024) FBXL4 mutation-caused mitochondrial DNA depletion syndrome is driven by BNIP3/BNIP3L-dependent excessive mitophagy. Trends Mol Med 30:113–116 38123379 10.1016/j.molmed.2023.11.017

[CR28] Gluais-Dagorn P, Foretz M, Steinberg GR, Batchuluun B, Zawistowska-Deniziak A, Lambooij JM, Guigas B, Carling D, Monternier PA, Moller DE et al (2022) Direct AMPK activation corrects nash in rodents through metabolic effects and direct action on inflammation and fibrogenesis. Hepatol Commun 6:101–119 34494384 10.1002/hep4.1799 PMC8710801

[CR29] Goodall EA, Kraus F, Harper JW (2022) Mechanisms underlying ubiquitin-driven selective mitochondrial and bacterial autophagy. Mol Cell 82:1501–1513 35364016 10.1016/j.molcel.2022.03.012 PMC9254164

[CR30] Handschin C, Spiegelman BM (2006) Peroxisome proliferator-activated receptor gamma coactivator 1 coactivators, energy homeostasis, and metabolism. Endocr Rev 27:728–735 17018837 10.1210/er.2006-0037

[CR31] Hickey KL, Swarup S, Smith IR, Paoli JC, Miguel Whelan E, Paulo JA, Harper JW (2023) Proteome census upon nutrient stress reveals Golgiphagy membrane receptors. Nature 623:167–174 37757899 10.1038/s41586-023-06657-6 PMC10620096

[CR32] Holm E, Vermeulen I, Parween S, Lopez-Perez A, Cillero-Pastor B, Vandenbosch M, Remeseiro S, Hornblad A (2025) AMPK activator ATX-304 reduces oxidative stress and improves MASLD via metabolic switching. JCI Insight 10:e179990 40197369 10.1172/jci.insight.179990 PMC11981618

[CR33] Huemer M, Karall D, Schossig A, Abdenur JE, Al Jasmi F, Biagosch C, Distelmaier F, Freisinger P, Graham BH, Haack TB et al (2015) Clinical, morphological, biochemical, imaging and outcome parameters in 21 individuals with mitochondrial maintenance defect related to FBXL4 mutations. J Inherit Metab Dis 38:905–914 25868664 10.1007/s10545-015-9836-6 PMC4841446

[CR34] Huss JM, Torra IP, Staels B, Giguere V, Kelly DP (2004) Estrogen-related receptor alpha directs peroxisome proliferator-activated receptor alpha signaling in the transcriptional control of energy metabolism in cardiac and skeletal muscle. Mol Cell Biol 24:9079–9091 15456881 10.1128/MCB.24.20.9079-9091.2004 PMC517878

[CR35] Jäger S, Handschin C, St.-Pierre J, Spiegelman BM (2007) AMP-activated protein kinase (AMPK) action in skeletal muscle via direct phosphorylation of PGC-1α. Proc Natl Acad Sci USA 104:12017–12022 17609368 10.1073/pnas.0705070104 PMC1924552

[CR36] Kazyken D, Dame SG, Wang C, Wadley M, Fingar DC (2024) Unexpected roles for AMPK in the suppression of autophagy and the reactivation of MTORC1 signaling during prolonged amino acid deprivation. Autophagy 20:2017–2040 38744665 10.1080/15548627.2024.2355074 PMC11346535

[CR37] Lemos C, Schulze VK, Baumgart SJ, Nevedomskaya E, Heinrich T, Lefranc J, Bader B, Christ CD, Briem H, Kuhnke LP et al (2021) The potent AMPK inhibitor BAY-3827 shows strong efficacy in androgen-dependent prostate cancer models. Cell Oncol 44:581–594 10.1007/s13402-020-00584-8 PMC12980684 33492659

[CR38] Longo M, Bishnu A, Risiglione P, Montava-Garriga L, Cuenco J, Sakamoto K, MacKintosh C, Ganley IG (2024) Opposing roles for AMPK in regulating distinct mitophagy pathways. Mol Cell 84:4350–4367.e4359 39532100 10.1016/j.molcel.2024.10.025

[CR39] Malik N, Ferreira BI, Hollstein PE, Curtis SD, Trefts E, Weiser Novak S, Yu J, Gilson R, Hellberg K, Fang L et al (2023) Induction of lysosomal and mitochondrial biogenesis by AMPK phosphorylation of FNIP1. Science 380:eabj5559 37079666 10.1126/science.abj5559 PMC10794112

[CR40] Martinez-Vicente M (2017) Neuronal mitophagy in neurodegenerative diseases. Front Mol Neurosci 10:64 28337125 10.3389/fnmol.2017.00064 PMC5340781

[CR41] McWilliams TG, Prescott AR, Allen GF, Tamjar J, Munson MJ, Thomson C, Muqit MM, Ganley IG (2016) mito-QC illuminates mitophagy and mitochondrial architecture in vivo. J Cell Biol 214:333–345 27458135 10.1083/jcb.201603039 PMC4970326

[CR42] Montava-Garriga L, Ganley IG (2020) Outstanding questions in mitophagy: what we do and do not know. J Mol Biol 432:206–230 31299243 10.1016/j.jmb.2019.06.032

[CR43] Montava-Garriga L, Singh F, Ball G, Ganley IG (2020) Semi-automated quantitation of mitophagy in cells and tissues. Mech Ageing Dev 185:111196 31843465 10.1016/j.mad.2019.111196 PMC6961211

[CR44] Monternier PA, Parasar P, Theurey P, Gluais Dagorn P, Kaur N, Nagaraja TN, Fouqueray P, Bolze S, Moller DE, Singh J et al (2022) Beneficial effects of the direct AMP-kinase activator PXL770 in in vitro and in vivo models of X-linked adrenoleukodystrophy. J Pharmacol Exp Ther 382:208–222 35764327 10.1124/jpet.122.001208 PMC11047065

[CR45] Myers RW, Guan HP, Ehrhart J, Petrov A, Prahalada S, Tozzo E, Yang X, Kurtz MM, Trujillo M, Gonzalez Trotter D et al (2017) Systemic pan-AMPK activator MK-8722 improves glucose homeostasis but induces cardiac hypertrophy. Science 357:507–511 28705990 10.1126/science.aah5582

[CR46] Narendra DP, Youle RJ (2024) The role of PINK1-Parkin in mitochondrial quality control. Nat Cell Biol 26:1639–1651 39358449 10.1038/s41556-024-01513-9

[CR47] Narkar VA, Downes M, Yu RT, Embler E, Wang YX, Banayo E, Mihaylova MM, Nelson MC, Zou Y, Juguilon H et al (2008) AMPK and PPARdelta agonists are exercise mimetics. Cell 134:405–415 18674809 10.1016/j.cell.2008.06.051 PMC2706130

[CR48] Negoita F, Fraguas Bringas C, Hellberg K, Luda KM, Liu H, Li Z, Cuenco J, Zhao JF, Sathe G, Ganley IG et al (2026) AMPK promotes TFEB transcriptional activity through dephosphorylation at both MTORC1-dependent and -independent sites. Autophagy 22:1386–1400 10.1080/15548627.2026.2629720 PMC13185439 41661247

[CR49] Nguyen-Dien GT, Kozul KL, Cui Y, Townsend B, Kulkarni PG, Ooi SS, Marzio A, Carrodus N, Zuryn S, Pagano M et al (2023) FBXL4 suppresses mitophagy by restricting the accumulation of NIX and BNIP3 mitophagy receptors. EMBO J 42:e112767 37161784 10.15252/embj.2022112767 PMC10308361

[CR50] Nguyen-Dien GT, Townsend B, Kulkarni PG, Kozul KL, Ooi SS, Eldershaw DN, Weeratunga S, Liu M, Jones MJ, Millard SS et al (2024) PPTC7 antagonizes mitophagy by promoting BNIP3 and NIX degradation via SCF(FBXL4). EMBO Rep 25:3324–3347 38992176 10.1038/s44319-024-00181-y PMC11316107

[CR51] Niemi NM, Serrano LR, Muehlbauer LK, Balnis CE, Wei L, Smith AJ, Kozul KL, Forny M, Connor OM, Rashan EH et al (2023) PPTC7 maintains mitochondrial protein content by suppressing receptor-mediated mitophagy. Nat Commun 14:6431 37833277 10.1038/s41467-023-42069-w PMC10575892

[CR52] Nwadike C, Williamson LE, Gallagher LE, Guan JL, Chan EYW (2018) AMPK inhibits ULK1-dependent autophagosome formation and lysosomal acidification via distinct mechanisms. Mol Cell Biol 38:e00023–18 29507183 10.1128/MCB.00023-18 PMC5954193

[CR53] Ordureau A, Kraus F, Zhang J, An H, Park S, Ahfeldt T, Paulo JA, Harper JW (2021) Temporal proteomics during neurogenesis reveals large-scale proteome and organelle remodeling via selective autophagy. Mol Cell 81:5082–5098.e5011 34699746 10.1016/j.molcel.2021.10.001 PMC8688335

[CR54] Park JM, Lee DH, Kim DH (2023) Redefining the role of AMPK in autophagy and the energy stress response. Nat Commun 14:2994 37225695 10.1038/s41467-023-38401-z PMC10209092

[CR55] Perry CG, Lally J, Holloway GP, Heigenhauser GJ, Bonen A, Spriet LL (2010) Repeated transient mRNA bursts precede increases in transcriptional and mitochondrial proteins during training in human skeletal muscle. J Physiol 588:4795–4810 20921196 10.1113/jphysiol.2010.199448 PMC3010147

[CR56] Pilegaard H, Saltin B, Neufer PD (2003) Exercise induces transient transcriptional activation of the PGC-1alpha gene in human skeletal muscle. J Physiol 546:851–858 12563009 10.1113/jphysiol.2002.034850 PMC2342594

[CR57] Raimi OG, Ojha H, Ehses K, Dederer V, Lange SM, Rivera CP, Deegan TD, Chen Y, Wightman M, Toth R et al (2024) Mechanism of human PINK1 activation at the TOM complex in a reconstituted system. Sci Adv 10:eadn7191 38848361 10.1126/sciadv.adn7191 PMC11160474

[CR58] Rena G, Pearson ER, Sakamoto K (2013) Molecular mechanism of action of metformin: old or new insights? Diabetologia 56:1898–1906 23835523 10.1007/s00125-013-2991-0 PMC3737434

[CR59] Rhein P, Desjardins EM, Rong P, Ahwazi D, Bonhoure N, Stolte J, Santos MD, Ovens AJ, Ehrlich AM, Sanchez Garcia JL et al (2021) Compound- and fiber type-selective requirement of AMPKgamma3 for insulin-independent glucose uptake in skeletal muscle. Mol Metab 51:101228 33798773 10.1016/j.molmet.2021.101228 PMC8381060

[CR60] Salpeter SR, Greyber E, Pasternak GA, Salpeter EE (2010) Risk of fatal and nonfatal lactic acidosis with metformin use in type 2 diabetes mellitus. Cochrane Database Syst Rev 2010:CD00296 10.1002/14651858.CD002967 12804446

[CR61] Sandoval H, Thiagarajan P, Dasgupta SK, Schumacher A, Prchal JT, Chen M, Wang J (2008) Essential role for Nix in autophagic maturation of erythroid cells. Nature 454:232–235 18454133 10.1038/nature07006 PMC2570948

[CR62] Schreiber SN, Emter R, Hock MB, Knutti D, Cardenas J, Podvinec M, Oakeley EJ, Kralli A (2004) The estrogen-related receptor alpha (ERRalpha) functions in PPARgamma coactivator 1alpha (PGC-1alpha)-induced mitochondrial biogenesis. Proc Natl Acad Sci USA 101:6472–6477 15087503 10.1073/pnas.0308686101 PMC404069

[CR63] Shu Y, Sheardown SA, Brown C, Owen RP, Zhang S, Castro RA, Ianculescu AG, Yue L, Lo JC, Burchard EG et al (2007) Effect of genetic variation in the organic cation transporter 1 (OCT1) on metformin action. J Clin Invest 117:1422–1431 17476361 10.1172/JCI30558 PMC1857259

[CR64] Singh F, Prescott AR, Rosewell P, Ball G, Reith AD, Ganley IG (2021) Pharmacological rescue of impaired mitophagy in Parkinson’s disease-related LRRK2 G2019S knock-in mice. Elife 10:e67604 34340748 10.7554/eLife.67604 PMC8331189

[CR65] Skaar JR, Pagan JK, Pagano M (2013) Mechanisms and function of substrate recruitment by F-box proteins. Nat Rev Mol Cell Biol 14:369–381 23657496 10.1038/nrm3582 PMC3827686

[CR66] Soucy TA, Smith PG, Milhollen MA, Berger AJ, Gavin JM, Adhikari S, Brownell JE, Burke KE, Cardin DP, Critchley S et al (2009) An inhibitor of NEDD8-activating enzyme as a new approach to treat cancer. Nature 458:732–736 19360080 10.1038/nature07884

[CR67] Sowter HM, Ratcliffe PJ, Watson P, Greenberg AH, Harris AL (2001) HIF-1-dependent regulation of hypoxic induction of the cell death factors BNIP3 and NIX in human tumors. Cancer Res 61:6669–6673 11559532

[CR68] Stanojlovic M, Bishnu A, Singh F, Ganley IG (2022) Physiological functions of mitophagy. Curr Opin Physiol 30:100612

[CR69] Steinberg GR, Carling D (2019) AMP-activated protein kinase: the current landscape for drug development. Nat Rev Drug Discov 18:527–551 30867601 10.1038/s41573-019-0019-2

[CR70] Steinberg GR, Hardie DG (2023) New insights into activation and function of the AMPK. Nat Rev Mol Cell Biol 24:255–272 36316383 10.1038/s41580-022-00547-x

[CR71] Sun Y, Cao Y, Wan H, Memetimin A, Cao Y, Li L, Wu C, Wang M, Chen S, Li Q et al (2024) A mitophagy sensor PPTC7 controls BNIP3 and NIX degradation to regulate mitochondrial mass. Mol Cell 84:327–344.e329 38151018 10.1016/j.molcel.2023.11.038

[CR72] Varanita T, Soriano ME, Romanello V, Zaglia T, Quintana-Cabrera R, Semenzato M, Menabo R, Costa V, Civiletto G, Pesce P et al (2015) The OPA1-dependent mitochondrial cristae remodeling pathway controls atrophic, apoptotic, and ischemic tissue damage. Cell Metab 21:834–844 26039448 10.1016/j.cmet.2015.05.007 PMC4457892

[CR73] Wilhelm LP, Zapata-Munoz J, Villarejo-Zori B, Pellegrin S, Freire CM, Toye AM, Boya P, Ganley IG (2022) BNIP3L/NIX regulates both mitophagy and pexophagy. EMBO J 41:e111115 36215693 10.15252/embj.2022111115 PMC9753467

[CR74] Winder WW, Holmes BF, Rubink DS, Jensen EB, Chen M, Holloszy JO (2000) Activation of AMP-activated protein kinase increases mitochondrial enzymes in skeletal muscle. J Appl Physiol 88:2219–2226 10846039 10.1152/jappl.2000.88.6.2219

[CR75] Wu Z, Puigserver P, Andersson U, Zhang C, Adelmant G, Mootha V, Troy A, Cinti S, Lowell B, Scarpulla RC et al (1999) Mechanisms controlling mitochondrial biogenesis and respiration through the thermogenic coactivator PGC-1. Cell 98:115–124 10412986 10.1016/S0092-8674(00)80611-X

[CR76] Zhao JF, Rodger CE, Allen GFG, Weidlich S, Ganley IG (2020) HIF1alpha-dependent mitophagy facilitates cardiomyoblast differentiation. Cell Stress 4:99–113 32420530 10.15698/cst2020.05.220 PMC7212530

[CR77] Zong H, Ren JM, Young LH, Pypaert M, Mu J, Birnbaum MJ, Shulman GI (2002) AMP kinase is required for mitochondrial biogenesis in skeletal muscle in response to chronic energy deprivation. Proc Natl Acad Sci USA 99:15983–15987 12444247 10.1073/pnas.252625599 PMC138551

