# Supplementary material for: Activation of AMPK as a therapeutic strategy for FBXL4-related mitochondrial DNA depletion syndrome
Source: EMBO Mol Med. 2026 Jun 17;18(8):3109–36. doi: 10.1038/s44321-026-00471-z (PMC13470155; doi:10.1038/s44321-026-00471-z)
Supplement: Supplementary file 1 [file 44321_2026_471_MOESM2_ESM.zip › Figure 1/1A/Fig 1A Raw Blots Croping border and Annotation.pdf]

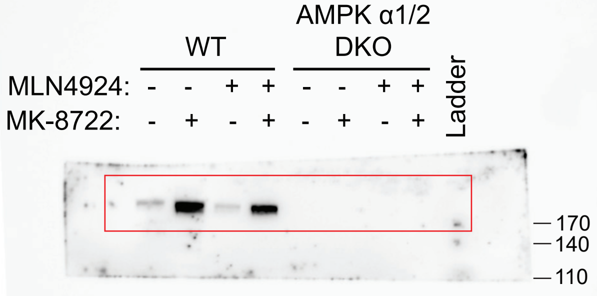

pSer80 ACC1

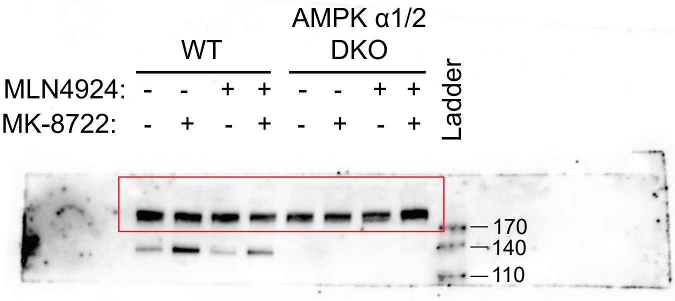

Total ACC1

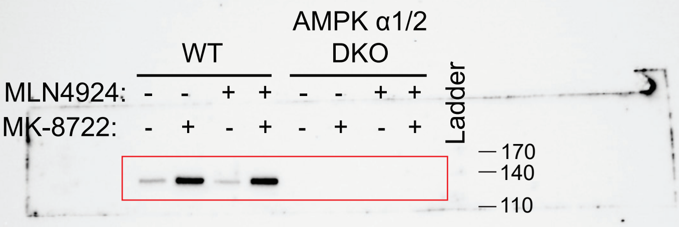

pSer556 ULK1

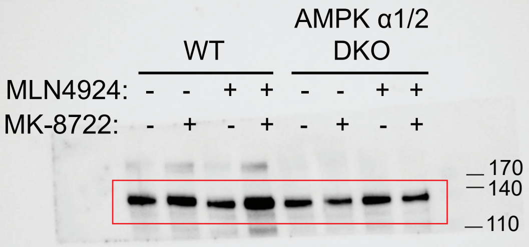

Total ULK1

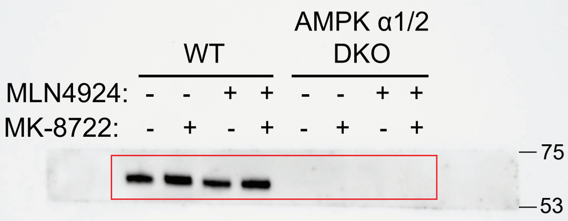

AMPK

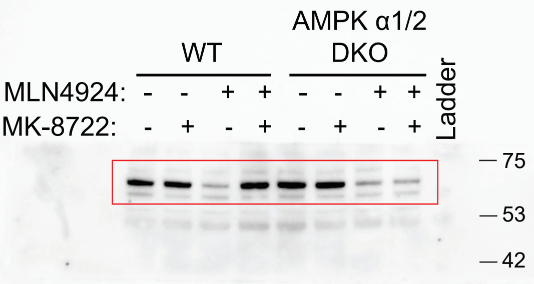

HSP60

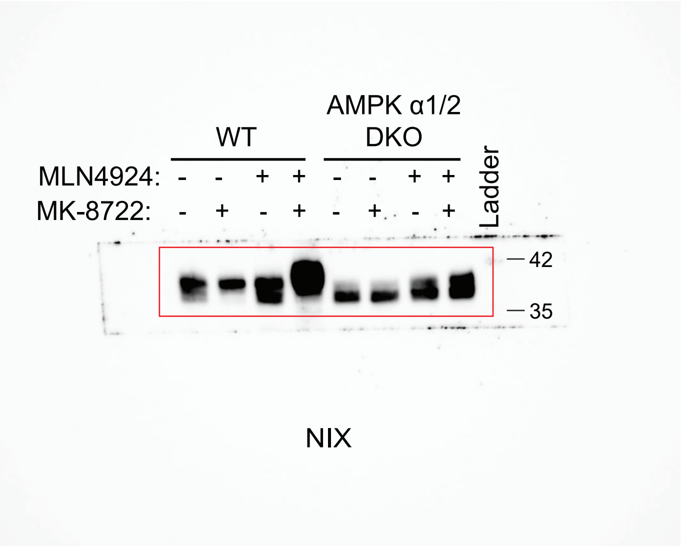
WT
AMPK α1/2 DKO
MLN4924: - - + + - - + +
MK-8722: - + - + - + - +
Ladder
42
35
NIX

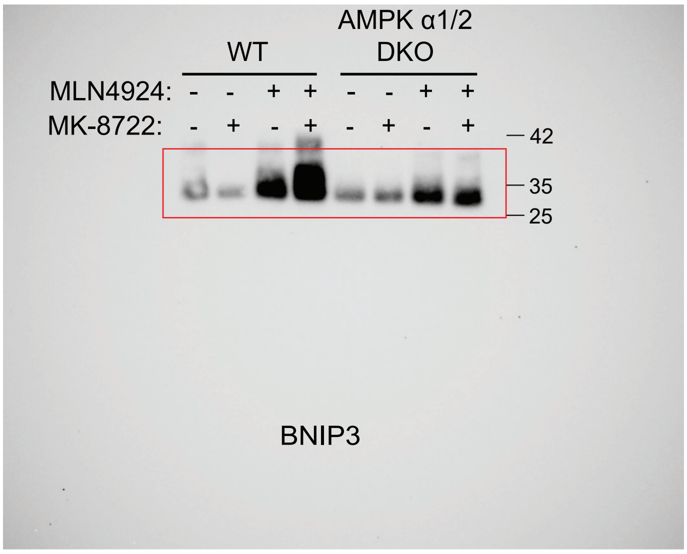
WT
AMPK α1/2 DKO
MLN4924: - - + + - - + +
MK-8722: - + - + - + - +
42
35
25
BNIP3

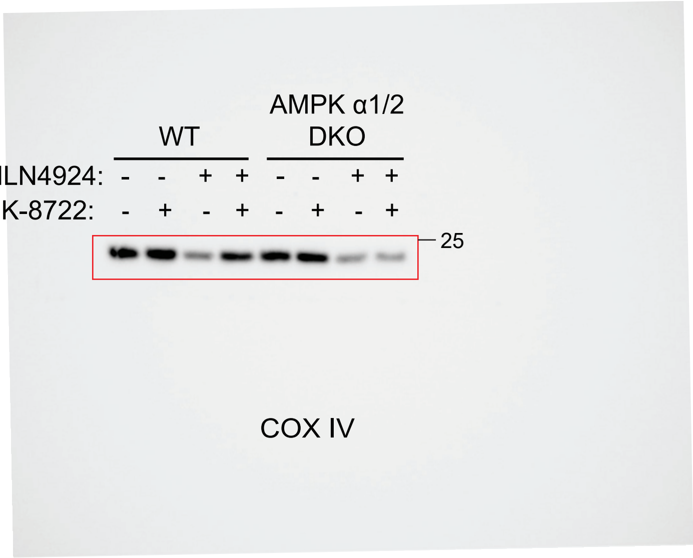
WT
AMPK α1/2 DKO
MLN4924: - - + + - - + +
MK-8722: - + - + - + - +
25
COX IV

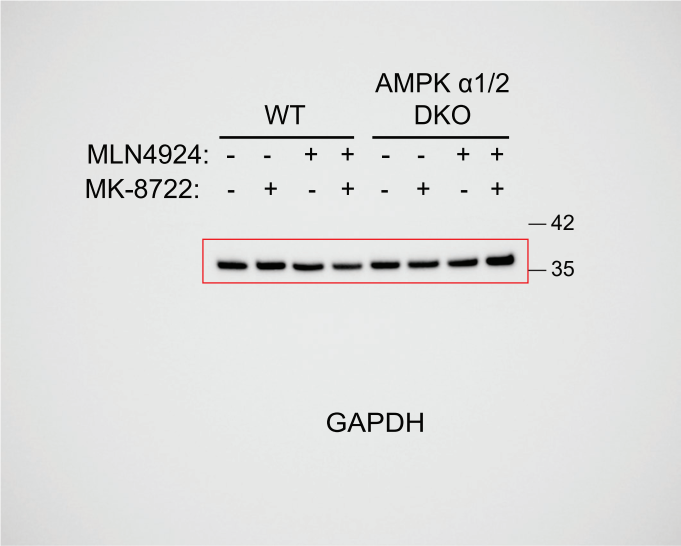
WT
AMPK α1/2 DKO
MLN4924: - - + + - - + +
MK-8722: - + - + - + - +
42
35
GAPDH
