# Supplementary material for: Activation of AMPK as a therapeutic strategy for FBXL4-related mitochondrial DNA depletion syndrome
Source: EMBO Mol Med. 2026 Jun 17;18(8):3109–36. doi: 10.1038/s44321-026-00471-z (PMC13470155; doi:10.1038/s44321-026-00471-z)
Supplement: Supplementary file 1 [file 44321_2026_471_MOESM2_ESM.zip › Figure 1/1C/Fig 1C Raw Blots Croping border and Annotation.pdf]

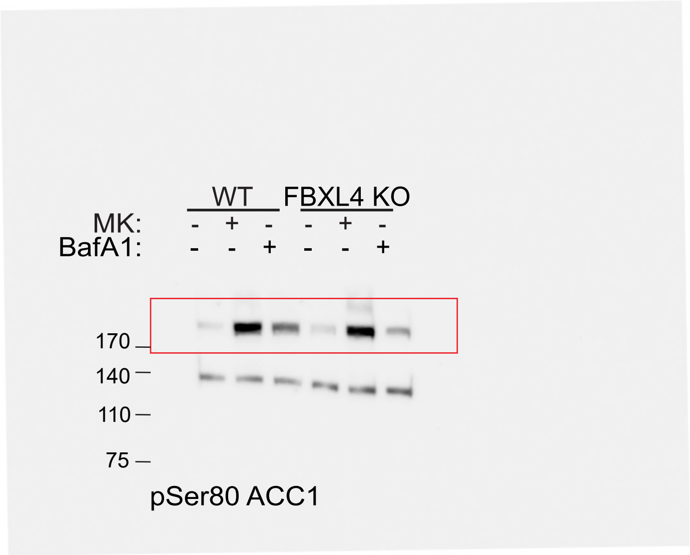
WT FBXL4 KO
MK: - + - - + -
BafA1: - - + - - +
170
140
110
75
pSer80 ACC1

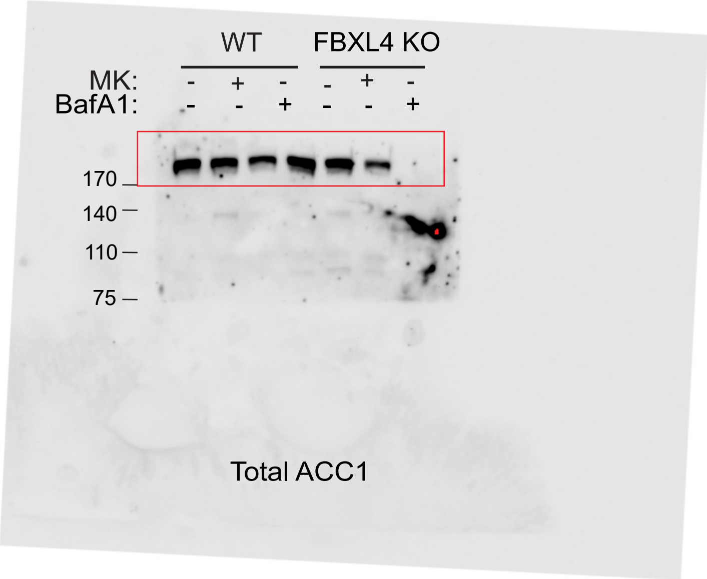
WT FBXL4 KO
MK: - + - - + -
BafA1: - - + - - +
170
140
110
75
Total ACC1

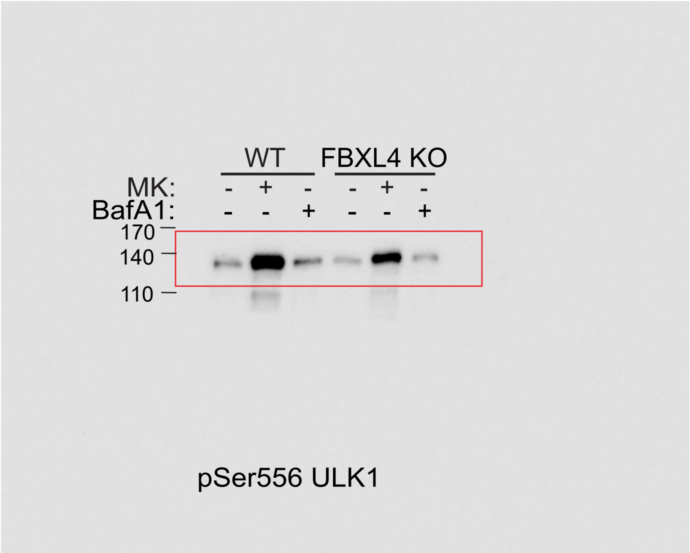
WT FBXL4 KO
MK: - + - - + -
BafA1: - - + - - +
170
140
110
pSer556 ULK1

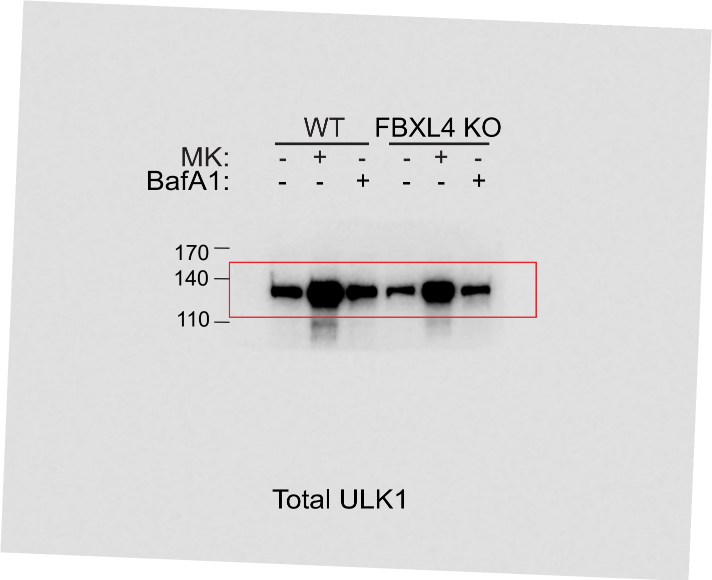
WT FBXL4 KO
MK: - + - - + -
BafA1: - - + - - +
170
140
110
Total ULK1

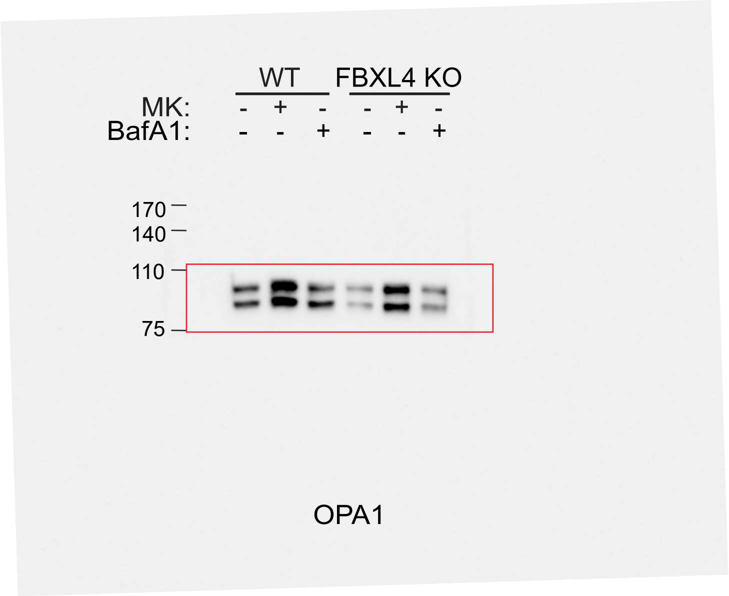
WT FBXL4 KO
MK: - + - - + -
BafA1: - - + - - +
170
140
110
75
OPA1

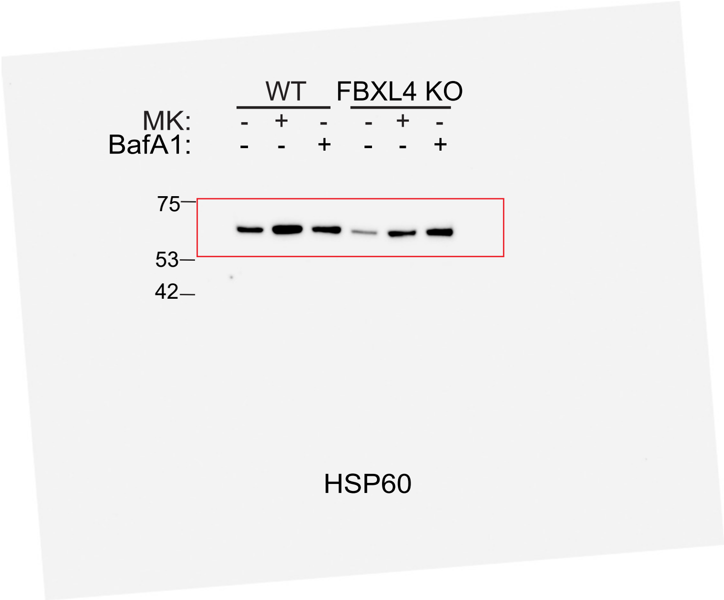
WT FBXL4 KO
MK: - + - - + -
BafA1: - - + - - +
75
53
42
HSP60

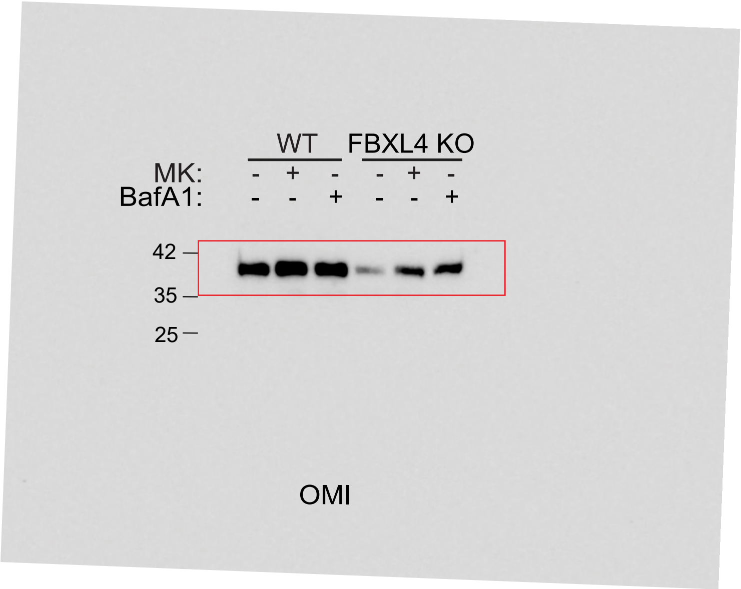
WT
FBXL4 KO
MK: - + - - + -
BafA1: - - + - - +
42
35
25
OMI

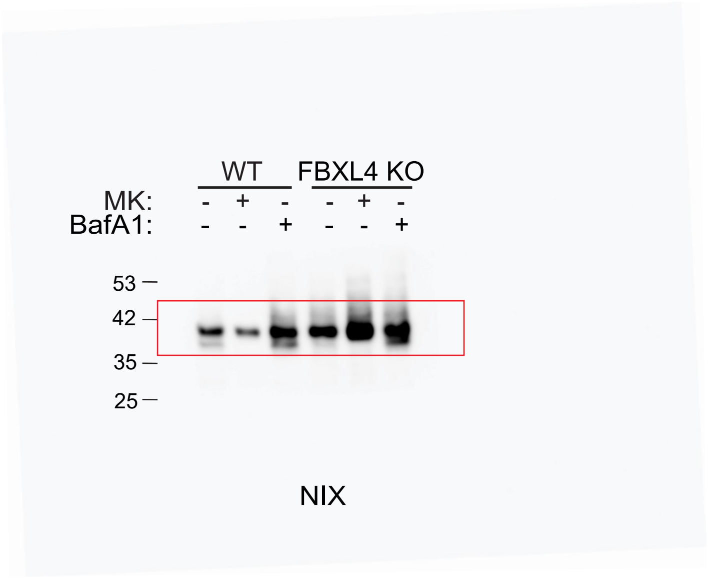
WT
FBXL4 KO
MK: - + - - + -
BafA1: - - + - - +
53
42
35
25
NIX

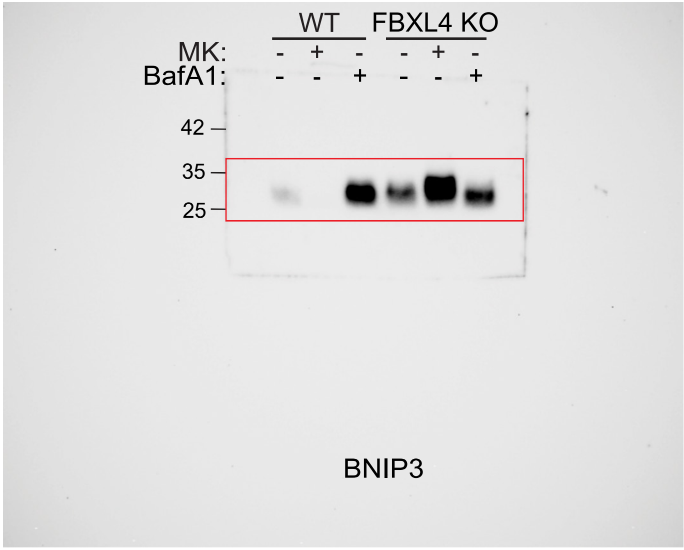
WT
FBXL4 KO
MK: - + - - + -
BafA1: - - + - - +
42
35
25
BNIP3

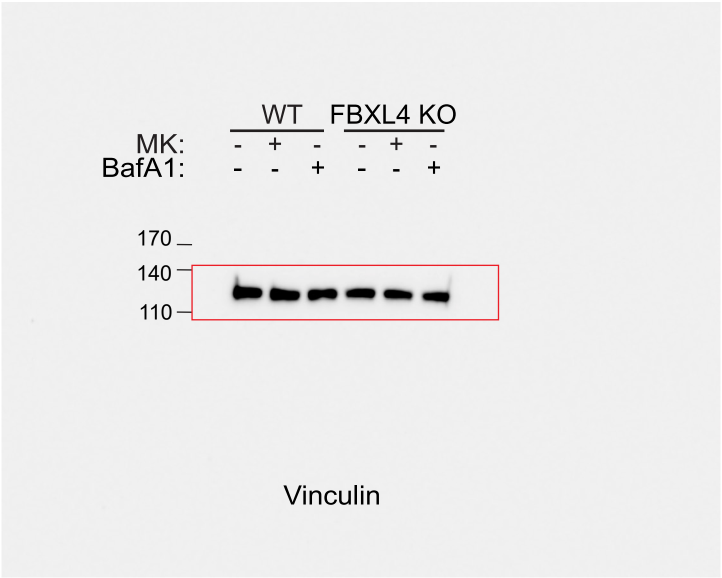
WT
FBXL4 KO
MK: - + - - + -
BafA1: - - + - - +
170
140
110
Vinculin
