# Supplementary material for: Activation of AMPK as a therapeutic strategy for FBXL4-related mitochondrial DNA depletion syndrome
Source: EMBO Mol Med. 2026 Jun 17;18(8):3109–36. doi: 10.1038/s44321-026-00471-z (PMC13470155; doi:10.1038/s44321-026-00471-z)
Supplement: Supplementary file 2 [file 44321_2026_471_MOESM3_ESM.zip › Figure 2/Fig 2A/Fig 2A Raw Blots Croping border and Annotation.pdf]

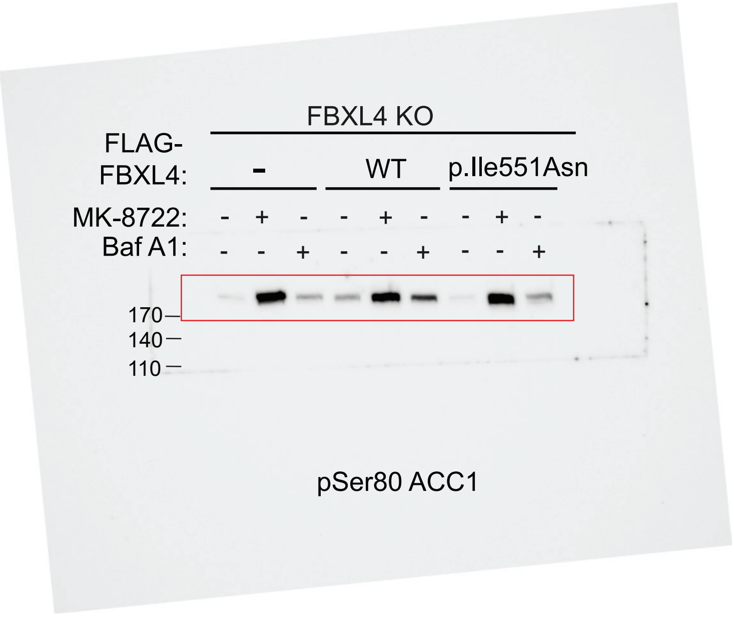
FBXL4 KO
FLAG-FBXL4:
-
WT
p.Ile551Asn
MK-8722: - + - - + - - + -
Baf A1: - - + - - + - - +
170
140
110
pSer80 ACC1

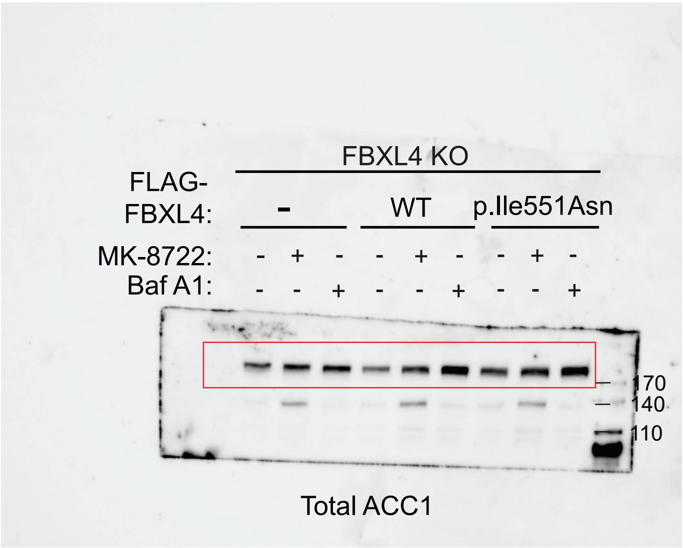
FBXL4 KO
FLAG-FBXL4:
-
WT
p.Ile551Asn
MK-8722: - + - - + - - + -
Baf A1: - - + - - + - - +
170
140
110
Total ACC1

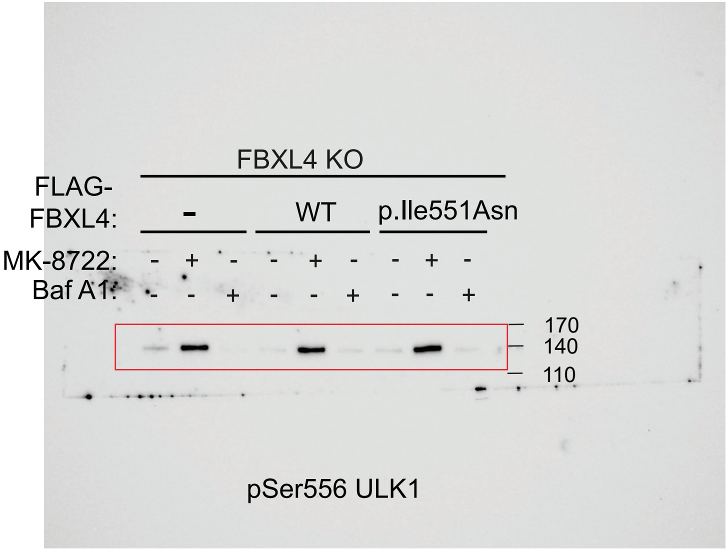
FBXL4 KO
FLAG-FBXL4:
-
WT
p.Ile551Asn
MK-8722: - + - - + - - + -
Baf A1: - - + - - + - - +
170
140
110
pSer556 ULK1

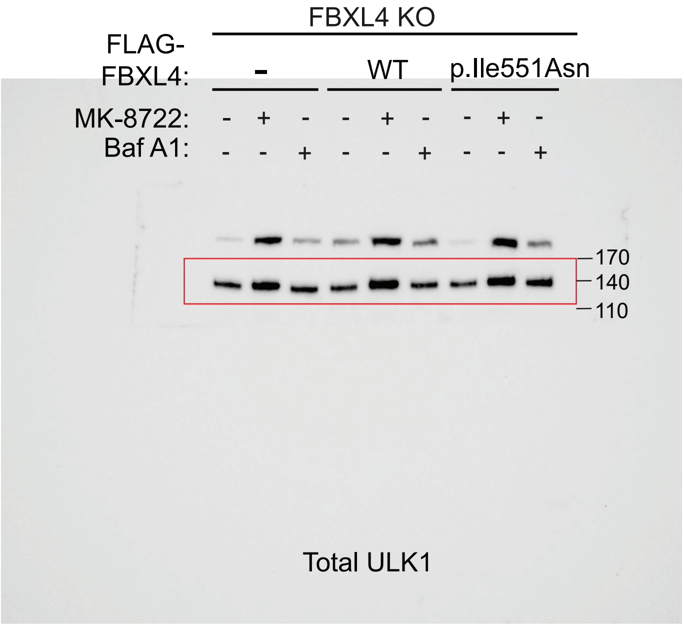
FBXL4 KO
FLAG-FBXL4:
-
WT
p.Ile551Asn
MK-8722: - + - - + - - + -
Baf A1: - - + - - + - - +
170
140
110
Total ULK1

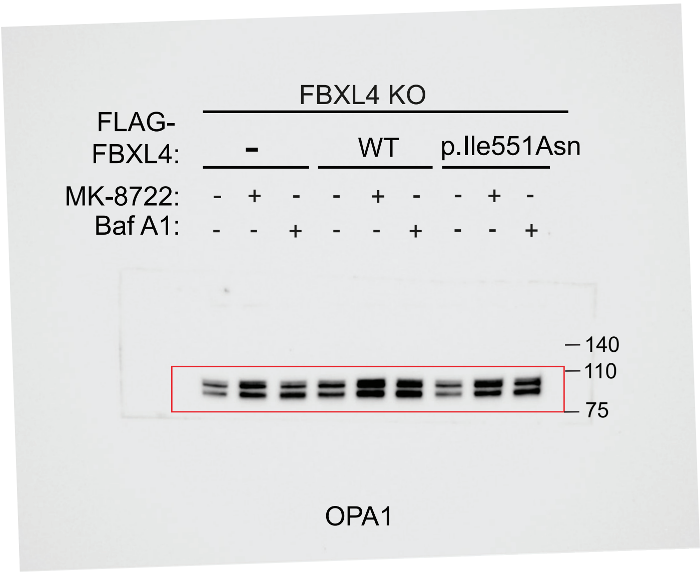
FBXL4 KO
FLAG-FBXL4:
-
WT
p.Ile551Asn
MK-8722: - + - - + - - + -
Baf A1: - - + - - + - - +
140
110
75
OPA1

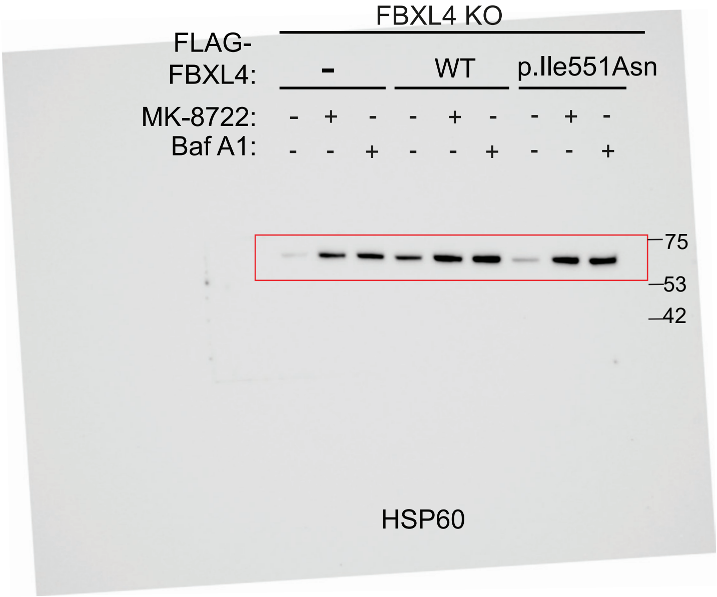
FBXL4 KO
FLAG-FBXL4:
-
WT
p.Ile551Asn
MK-8722: - + - - + - - + -
Baf A1: - - + - - + - - +
75
53
42
HSP60

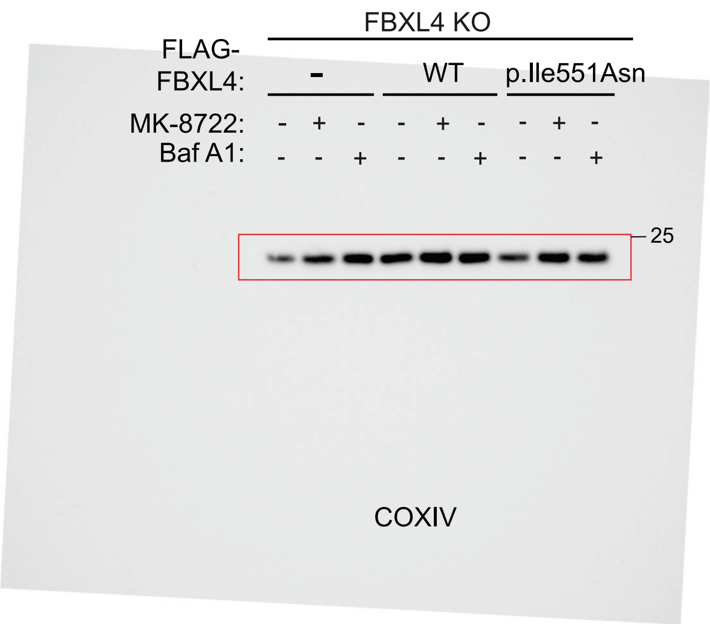
FBXL4 KO
FLAG-FBXL4:
-
WT
p.Ile551Asn
MK-8722: - + - - + - - + -
Baf A1: - - + - - + - - +
25
COXIV

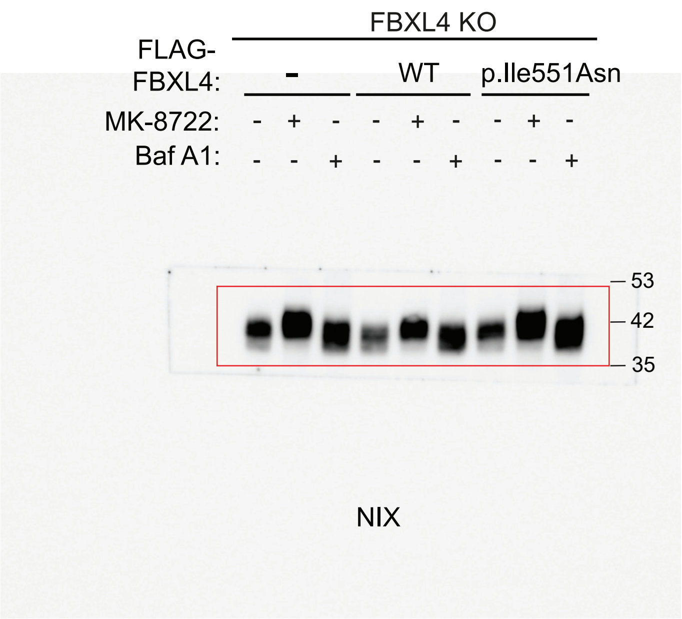
FBXL4 KO
FLAG-FBXL4:
-
WT
p.Ile551Asn
MK-8722: - + - - + - - + -
Baf A1: - - + - - + - - +
53
42
35
NIX

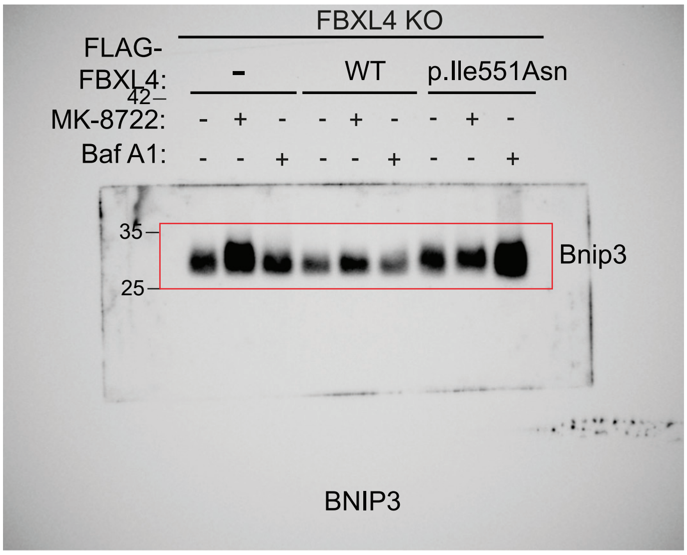
FBXL4 KO
FLAG-FBXL4:
-
WT
p.Ile551Asn
42
MK-8722: - + - - + - - + -
Baf A1: - - + - - + - - +
35
25
Bnip3
BNIP3

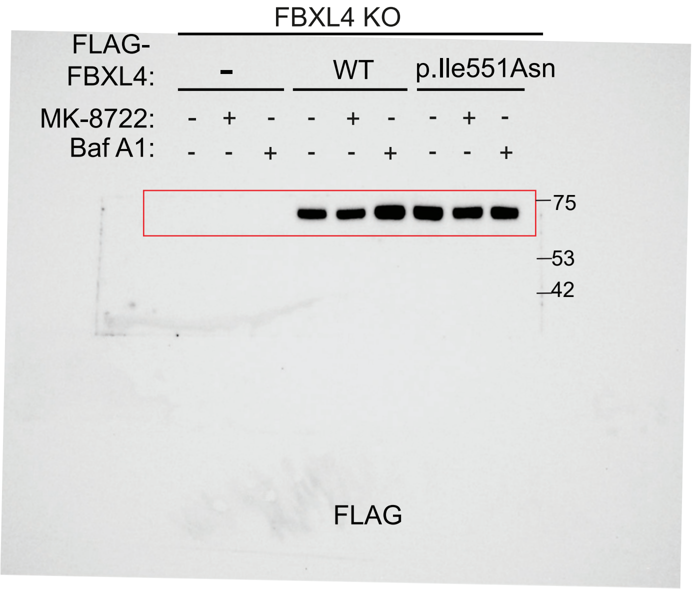
FBXL4 KO
FLAG-FBXL4:
-
WT
p.Ile551Asn
MK-8722: - + - - + - - + -
Baf A1: - - + - - + - - +
75
53
42
FLAG

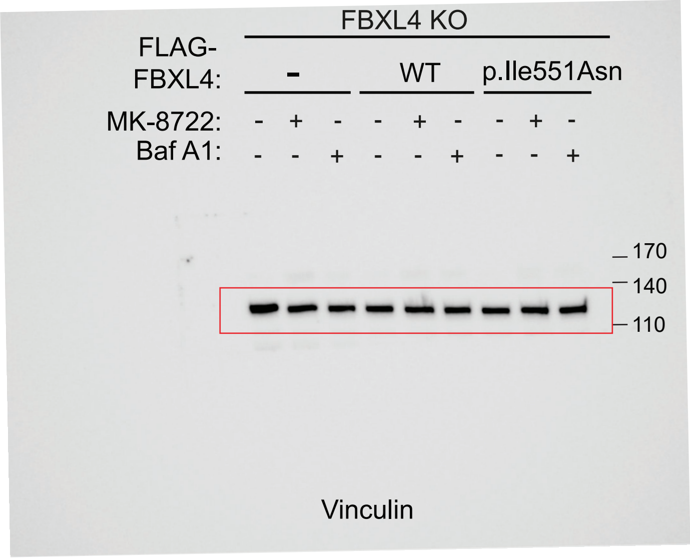
FBXL4 KO
FLAG-FBXL4:
-
WT
p.Ile551Asn
MK-8722: - + - - + - - + -
Baf A1: - - + - - + - - +
170
140
110
Vinculin
