# Supplementary material for: Activation of AMPK as a therapeutic strategy for FBXL4-related mitochondrial DNA depletion syndrome
Source: EMBO Mol Med. 2026 Jun 17;18(8):3109–36. doi: 10.1038/s44321-026-00471-z (PMC13470155; doi:10.1038/s44321-026-00471-z)
Supplement: Supplementary file 3 [file 44321_2026_471_MOESM4_ESM.zip › Figure 3/Fig 3A/Fig 3A Raw Blots Croping border and Annotation.pdf]

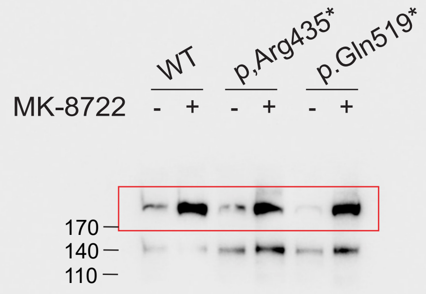

pSer80 ACC1

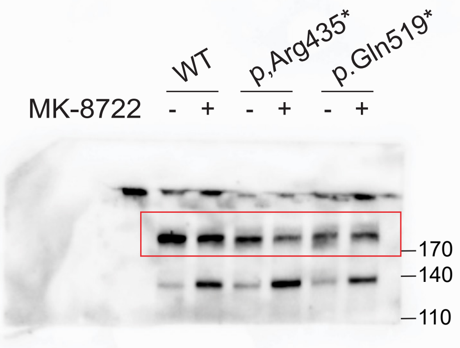

Total ACC1

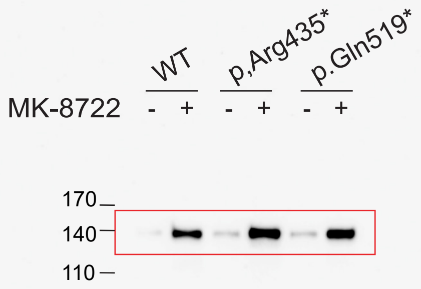

pSer556 ULK1

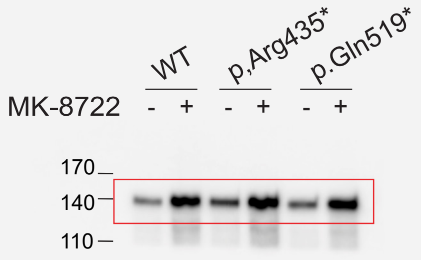

Total ULK1

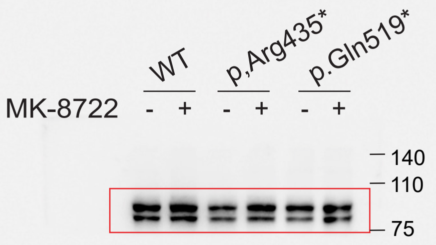

OPA1

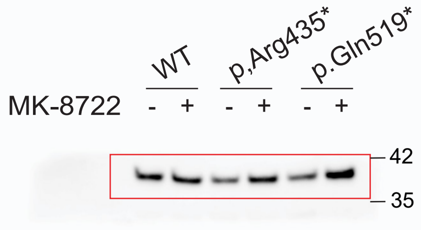

OMI

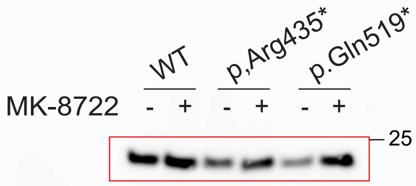

COX IV

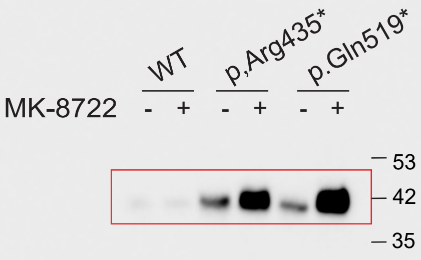

NIX

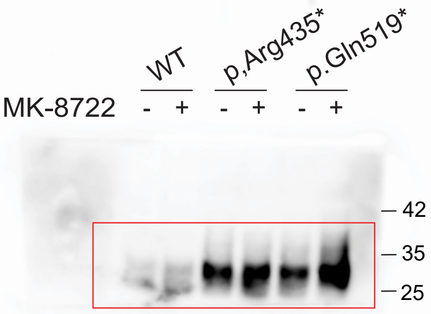

Bnip3

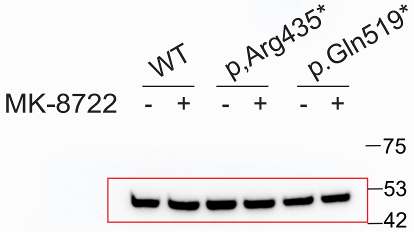

Tubulin
