# Supplementary material for: Activation of AMPK as a therapeutic strategy for FBXL4-related mitochondrial DNA depletion syndrome
Source: EMBO Mol Med. 2026 Jun 17;18(8):3109–36. doi: 10.1038/s44321-026-00471-z (PMC13470155; doi:10.1038/s44321-026-00471-z)
Supplement: Supplementary file 3 [file 44321_2026_471_MOESM4_ESM.zip › Figure 3/Fig 3D/Read Me.docx]

Each excel depicts semi-automated quantification of the number of mitolysosome in wide field images of WT, p,Arg435* and p.Gln519* cells. Each ROI represents a single cell. Average number of mitolysosome was calculated for each cell treated with either DMSO or MK-8722. The Average of 3 independent experiment was used to calculate the mean number of mitolysosome per cell.

In the final figure

1. WT UT represents WT MK (–)
2. WT MK represents WT MK (+)
3. R435 UT represents p,Arg435* MK (–)
4. R435 MK represents p,Arg435* MK (+)
5. Q519 UT represents p.Gln519* MK (–)
6. Q519 MK represents p.Gln519* MK (+)
