# Supplementary material for: Activation of AMPK as a therapeutic strategy for FBXL4-related mitochondrial DNA depletion syndrome
Source: EMBO Mol Med. 2026 Jun 17;18(8):3109–36. doi: 10.1038/s44321-026-00471-z (PMC13470155; doi:10.1038/s44321-026-00471-z)
Supplement: Supplementary file 4 [file 44321_2026_471_MOESM5_ESM.zip › Figure 4/Fig 4A/Fig 4A Raw Blots Croping border and Annotation.pdf]

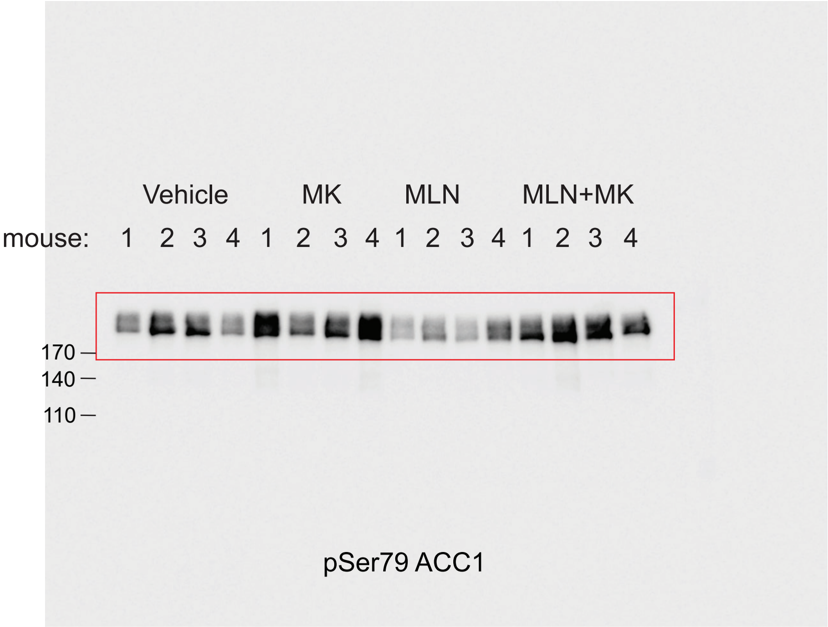
Vehicle
MK
MLN
MLN+MK
mouse: 1 2 3 4 1 2 3 4 1 2 3 4 1 2 3 4
170
140
110
pSer79 ACC1

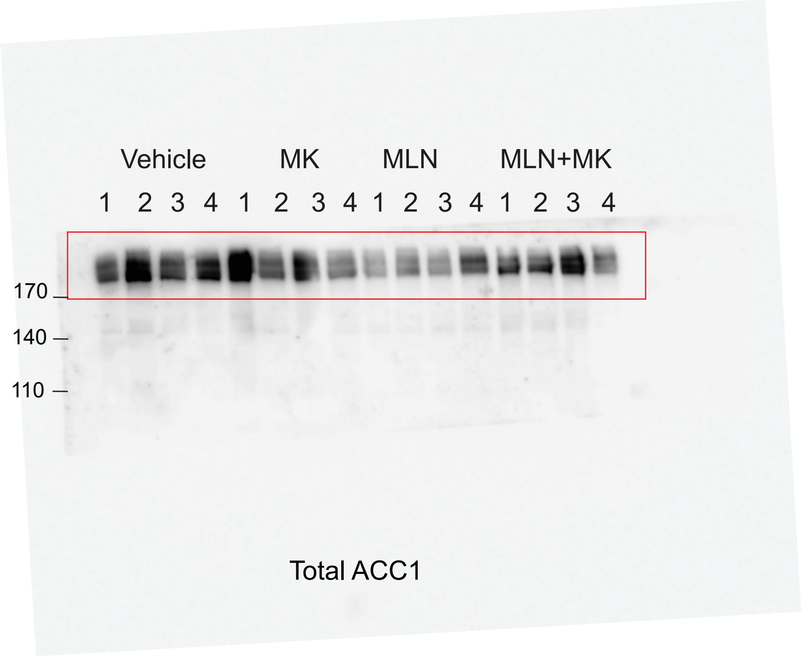
Vehicle
MK
MLN
MLN+MK
1 2 3 4 1 2 3 4 1 2 3 4 1 2 3 4
170
140
110
Total ACC1

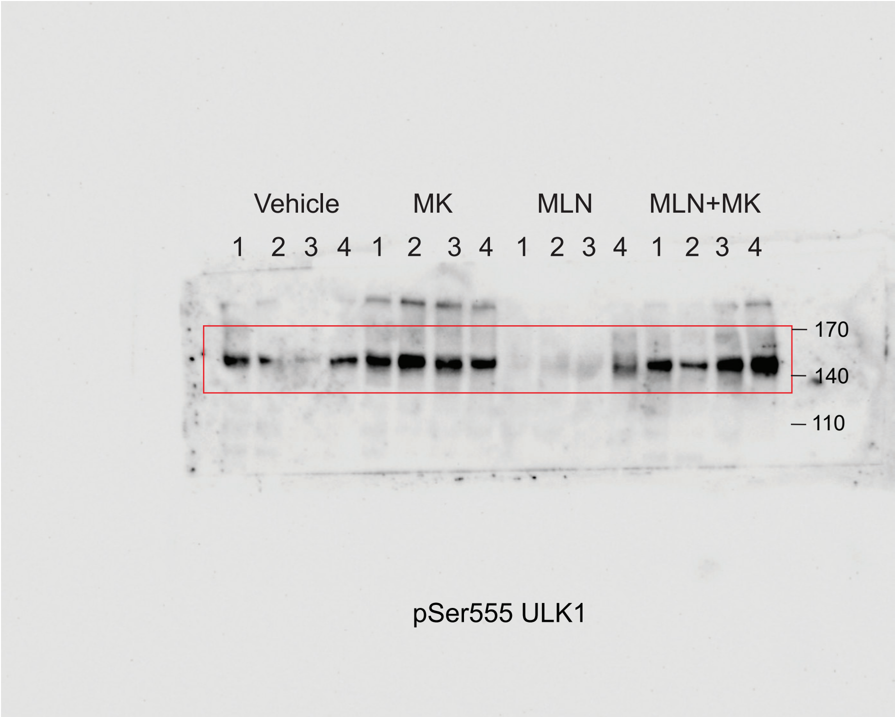
Vehicle
MK
MLN
MLN+MK
1 2 3 4 1 2 3 4 1 2 3 4 1 2 3 4
170
140
110
pSer555 ULK1

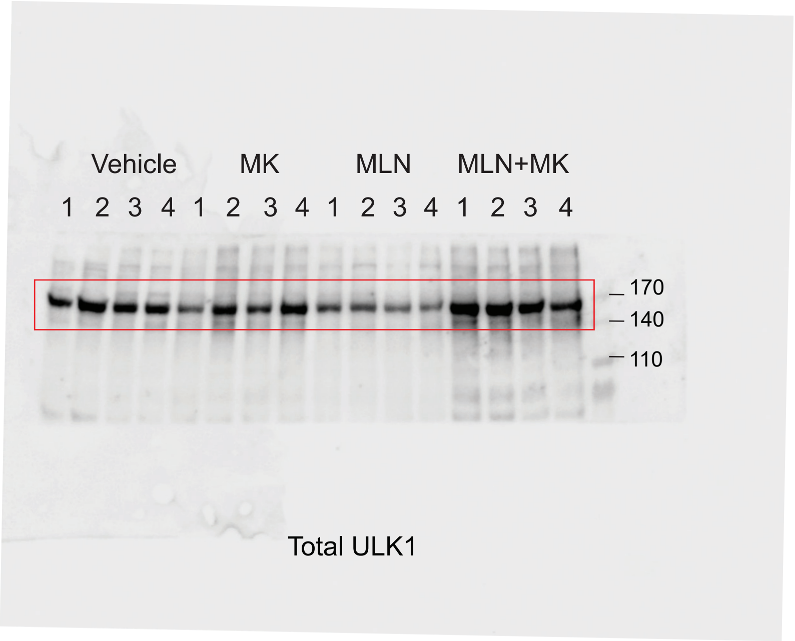

Total ULK1

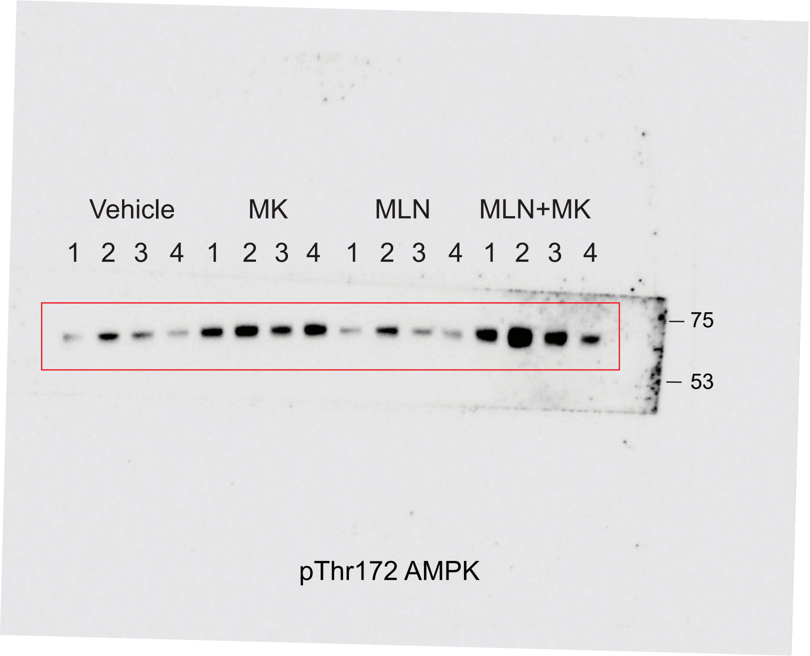

pThr172 AMPK

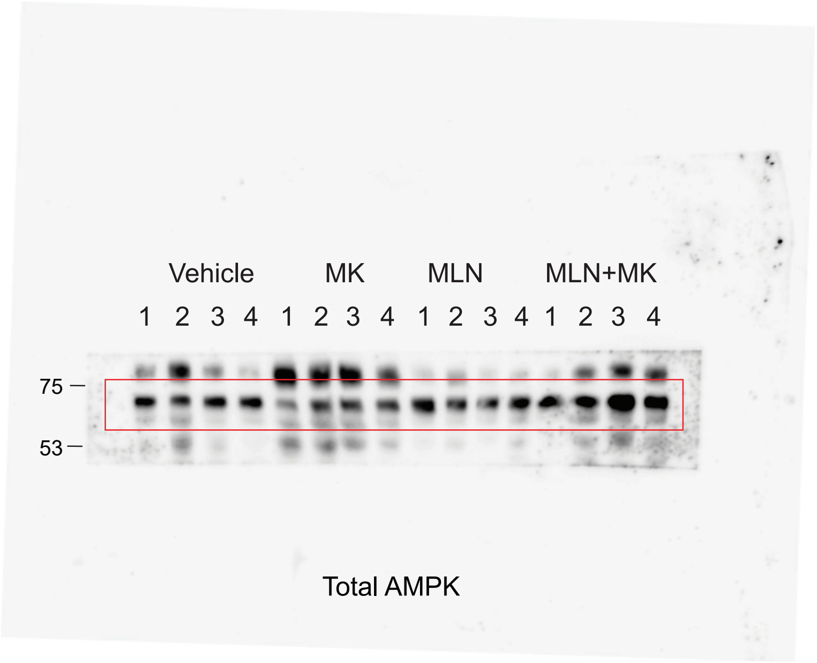

Total AMPK

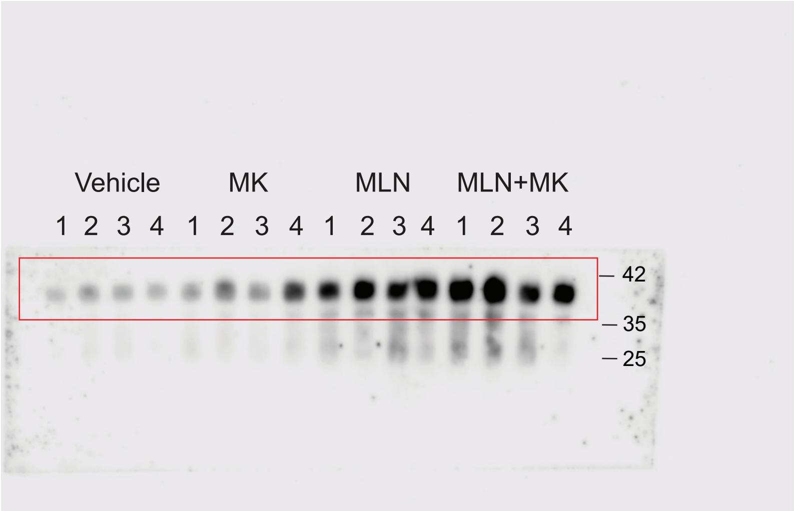

NIX

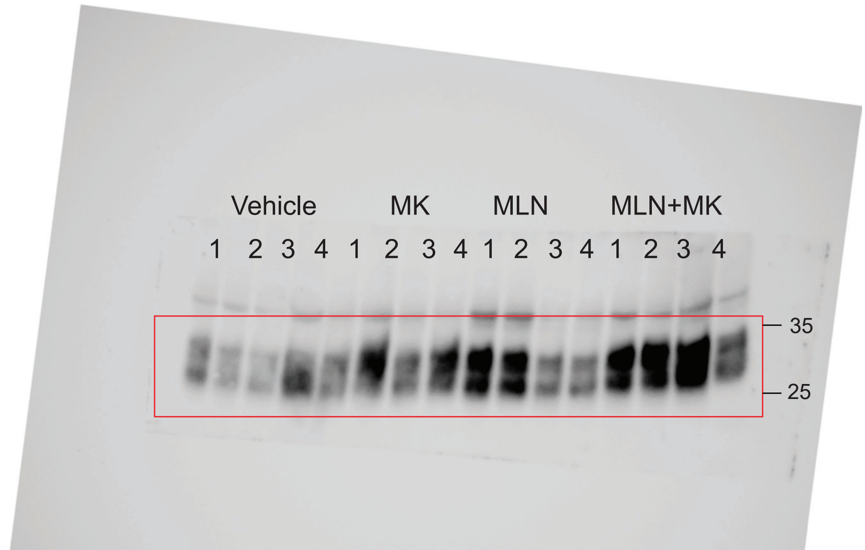

BNIP3

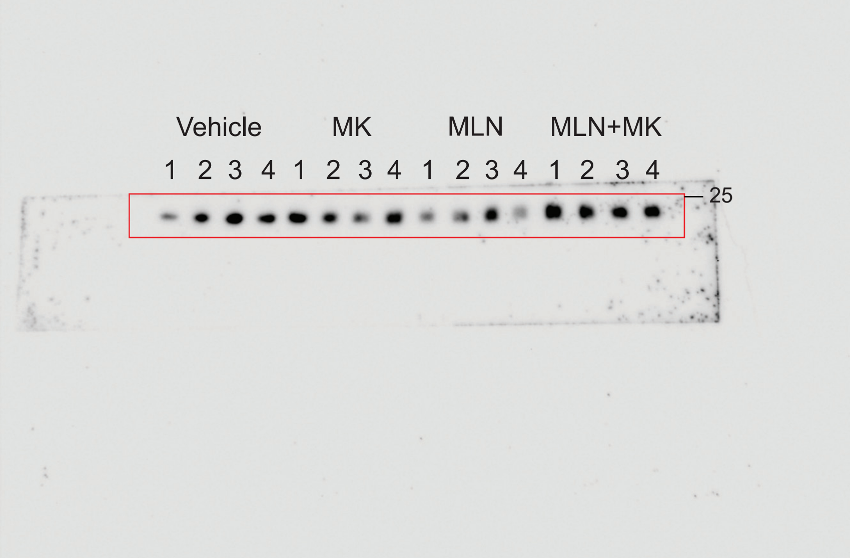

COXIV

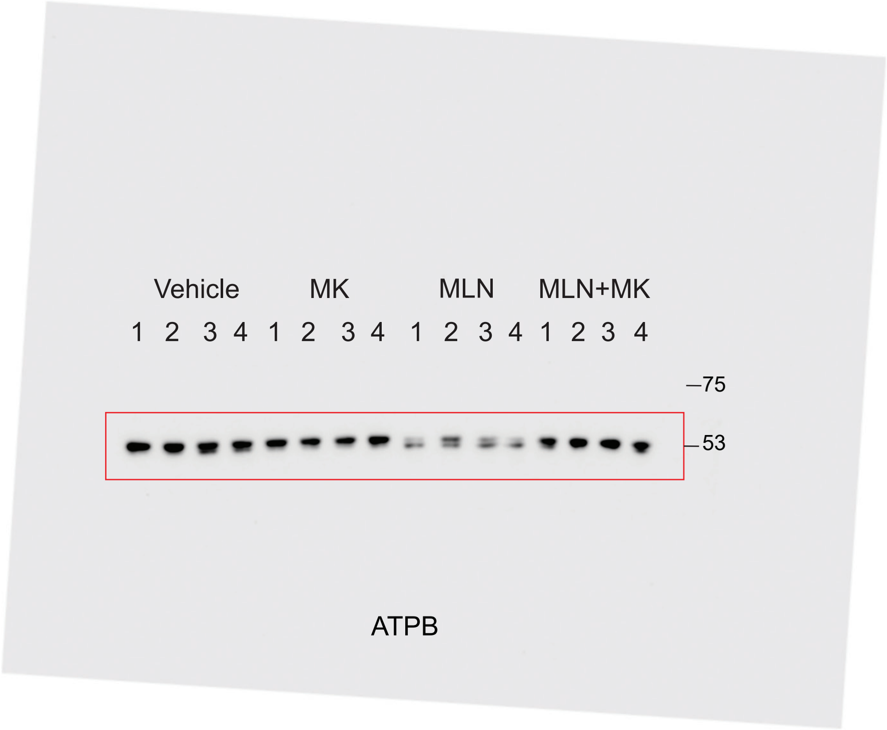
Vehicle MK MLN MLN+MK
1 2 3 4 1 2 3 4 1 2 3 4 1 2 3 4
75
53
ATPB

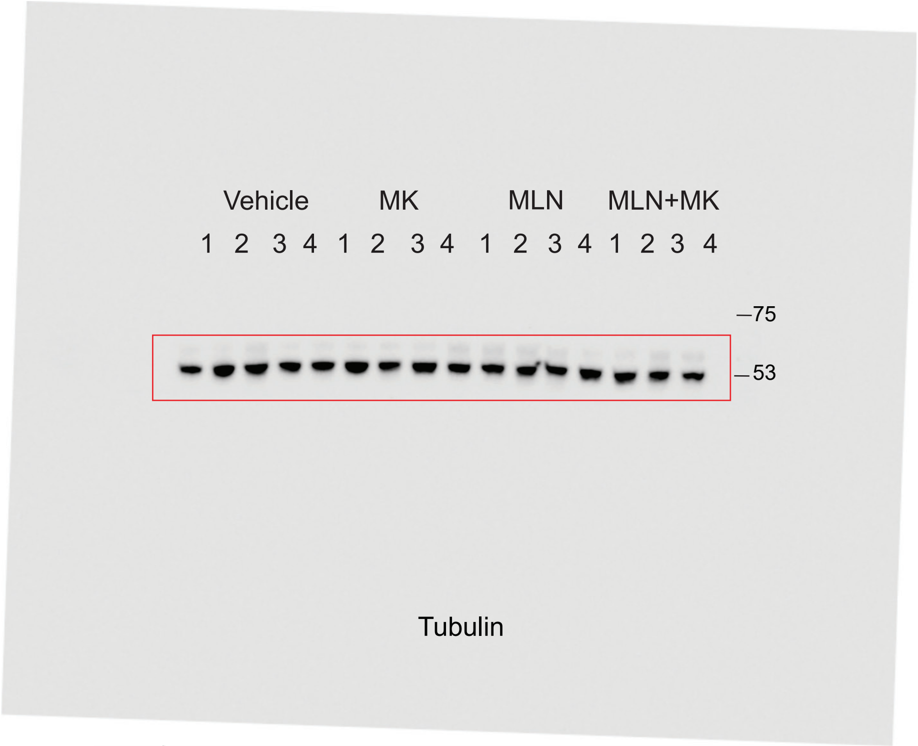
Vehicle MK MLN MLN+MK
1 2 3 4 1 2 3 4 1 2 3 4 1 2 3 4
75
53
Tubulin

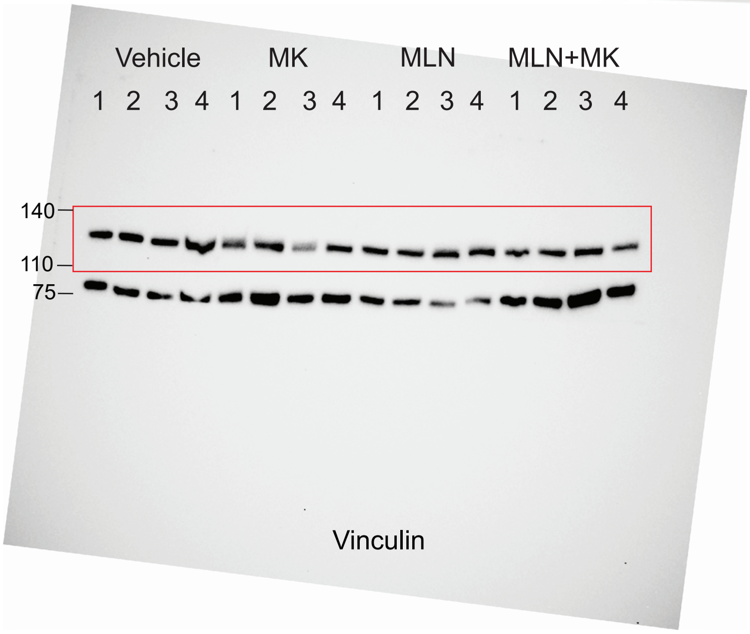
Vehicle MK MLN MLN+MK
1 2 3 4 1 2 3 4 1 2 3 4 1 2 3 4
140
110
75
Vinculin
