# Supplementary material for: Activation of AMPK as a therapeutic strategy for FBXL4-related mitochondrial DNA depletion syndrome
Source: EMBO Mol Med. 2026 Jun 17;18(8):3109–36. doi: 10.1038/s44321-026-00471-z (PMC13470155; doi:10.1038/s44321-026-00471-z)
Supplement: Supplementary file 5 [file 44321_2026_471_MOESM6_ESM.zip › Figure 5/Fig 5C/Read Me.docx]

Semi-automated quantification of the number of mitolysosome in each image acquired from various fields of two tissue sections from each animal of four groups. Each image represents a different field of view. The Images are labelled as follows Treatment Group Animal ID Image ID. The Average mitolysosome no/Area was calculated from 4-5 different field of view of each animal. The Average mitolysosome no/Area of 8 animals was used to calculate the mean number of mitolysosome/Area of each treatment group.
