# Supplementary material for: Activation of AMPK as a therapeutic strategy for FBXL4-related mitochondrial DNA depletion syndrome
Source: EMBO Mol Med. 2026 Jun 17;18(8):3109–36. doi: 10.1038/s44321-026-00471-z (PMC13470155; doi:10.1038/s44321-026-00471-z)
Supplement: Supplementary file 6 [file 44321_2026_471_MOESM7_ESM.docx]

| **Figure 1** |  |  |
| --- | --- | --- |
| **Figure 1B** |  |  |
|  |  |  |
| **pS80 ACC1/ACC1** | Summary | Adjusted P Value |
| AMPK WT DMSO vs. MK-8722 | * | 0.0267 |
| AMPK WT MLN-4924 vs. MLN4924+MK-8722 | * | 0.0186 |
| AMPK WT MK-8722 vs. AMPK DKO MK-8722 | *** | 0.0004 |
| AMPK WT MLN4924+MK-8722 vs. AMPK DKO MLN4924+MK-8722 | *** | 0.0002 |
|  |  |  |
| **pS556 ULK1/ULK1** |  |  |
| AMPK WT DMSO vs. MK-8722 | ** | 0.0066 |
| AMPK WT MLN-4924 vs. MLN4924+MK-8722 | * | 0.0385 |
| AMPK WT MK-8722 vs. AMPK DKO MK-8722 | **** | <0.0001 |
| AMPK WT MLN4924+MK-8722 vs. AMPK DKO MLN4924+MK-8722 | **** | <0.0001 |
|  |  |  |
|  |  |  |
| **HSP60 levels** |  |  |
| AMPK WT DMSO vs. MLN-4924 | * | 0.0226 |
| AMPK WT MLN-4924 vs. MLN4924+MK-8722 | ** | 0.0013 |
| AMPK DKO DMSO vs. MLN-4924 | *** | 0.0007 |
| AMPK WT MLN4924+MK-8722 vs. AMPK DKO MLN4924+MK-8722 | **** | <0.0001 |
| AMPK DKO MLN-4924 vs. MLN4924+MK-8722 | ns | 0.9983 |
|  |  |  |
|  |  |  |
| **COXIV levels** |  |  |
| AMPK WT DMSO vs. MLN-4924 | * | 0.0121 |
| AMPK WT MLN-4924 vs. MLN4924+MK-8722 | * | 0.0237 |
| AMPK DKO DMSO vs. MLN-4924 | *** | 0.0006 |
| AMPK WT MLN4924+MK-8722 vs. AMPK DKO MLN4924+MK-8722 | **** | <0.0001 |
| AMPK DKO MLN-4924 vs. MLN4924+MK-8722 | ns | 0.9998 |
|  |  |  |
|  |  |  |
| **Figure 1D** |  |  |
|  |  |  |
| **OPA1 levels** |  |  |
| FBXL4 WT DMSO vs KO DMSO | *** | 0.0005 |
| FBXL4 KO DMSO vs KO MK-8722 | ** | 0.0015 |
| FBXL4 KO DMSO vs KO BafA1 | * | 0.0133 |
|  |  |  |
| **HSP60 levels** |  |  |
| FBXL4 WT DMSO vs KO DMSO | ** | 0.0013 |
| FBXL4 KO DMSO vs KO MK-8722 | * | 0.0218 |
| FBXL4 KO DMSO vs KO BafA1 | ** | 0.0038 |
|  |  |  |
| **OMI levels** |  |  |
| FBXL4 WT DMSO vs KO DMSO | ** | 0.0057 |
| FBXL4 KO DMSO vs KO MK-8722 | * | 0.0267 |
| FBXL4 KO DMSO vs KO BafA1 | ** | 0.009 |
|  |  |  |
| **NIX levels** |  |  |
| FBXL4 WT DMSO vs KO DMSO | * | 0.0299 |
| FBXL4 KO DMSO vs KO MK-8722 | * | 0.0485 |
| FBXL4 KO DMSO vs KO BafA1 | * | 0.045 |
|  |  |  |
| **Figure 1F** |  |  |
| FBXL4 WT DMSO vs KO DMSO | **** | <0.0001 |
| FBXL4 KO DMSO vs KO MK-8722 | **** | <0.0001 |
|  |  |  |
|  |  |  |
| **Figure EV1** |  |  |
| **Fig EV1 A** |  |  |
|  |  |  |
| WT DMSO vs WT MLN4924 | **** | <0.0001 |
| NIX KO DMSO vs NIK KO MLN4924 | ns | 0.0514 |
| BNIP3 KO DMSO vs BNIP3 KO MLN4924 | ** | 0.0014 |
| NIX/BNIP3 KO DMSO vs  NIX/BNIP3 KO MLN4924 | ns | 0.721 |
| WT MLN4924 vs. NIX KO MLN4924 | **** | <0.0001 |
| WT MLN4924 vs. BNIP3 KO MLN4924 | **** | <0.0001 |
| WT MLN4924 vs. NIX/BNIP3 DKO MLN4924 | **** | <0.0001 |
| NIX KO MLN4924 vs. NIX/BNIP3 DKO MLN4924 | ns | 0.4239 |
| BNIP3 KO MLN4924 vs. NIX/BNIP3 DKO MLN4924 | ** | 0.0048 |
|  |  |  |
|  |  |  |
| **Fig EV1 C** |  |  |
| **Catalase levels** |  |  |
| FBXL4 WT DMSO v KO DMSO | ns | 0.68 |
| FBXL4 WT DMSO v WT MK-8722 | ns | 0.9142 |
| FBXL4 KO DMSO vs KO MK-8722 | ns | 0.5542 |
|  |  |  |
| **PMP70 levels** |  |  |
| FBXL4 WT DMSO v KO DMSO | ns | 0.8605 |
| FBXL4 WT DMSO v WT MK-8722 | ns | 0.7317 |
| FBXL4 KO DMSO vs KO MK-8722 | ns | 0.9458 |
|  |  |  |
| **COX IV levels** |  |  |
| FBXL4 WT DMSO v KO DMSO | ** | 0.0082 |
| FBXL4 WT DMSO v WT MK-8722 | ns | 0.2541 |
| FBXL4 KO DMSO vs KO MK-8722 | * | 0.0204 |
|  |  |  |
|  |  |  |
| **Fig EV1 E** |  |  |
| **pS80 ACC1/ACC1** |  |  |
| KO DMSO vs. KO MK | ** | 0.0044 |
| KO DMSO vs. KO PXL | * | 0.0481 |
| KO DMSO vs. KO BI | ** | 0.0056 |
|  |  |  |
| **pS556 ULK1/ULK1** |  |  |
| KO DMSO vs. KO MK | * | 0.013 |
| KO DMSO vs. KO PXL | * | 0.0215 |
| KO DMSO vs. KO BI | * | 0.0138 |
|  |  |  |
| **OPA1 levels** |  |  |
| WT DMSO vs KO DMSO | * | 0.0174 |
| KO DMSO vs. KO MK | *** | 0.0001 |
| KO DMSO vs. KO PXL | *** | 0.0005 |
| KO DMSO vs. KO BI | ** | 0.0011 |
|  |  |  |
| **HSP60 levels** |  |  |
| WT DMSO vs KO DMSO | ** | 0.0054 |
| KO DMSO vs. KO MK | * | 0.013 |
| KO DMSO vs. KO PXL | ** | 0.0018 |
| KO DMSO vs. KO BI | ** | 0.0085 |
|  |  |  |
|  |  |  |
| **Fig EV1G** |  |  |
| WT DMSO vs KO DMSO | **** | <0.0001 |
| KO DMSO vs. KO MK | **** | <0.0001 |
| KO DMSO vs. KO PXL | *** | 0.0005 |
| KO DMSO vs. KO BI | *** | 0.0001 |
|  |  |  |
|  |  |  |
| **Fig EV1I** |  |  |
| **pS80 ACC1/ACC1** |  |  |
| DMSO vs. MK | *** | 0.0002 |
| MK vs. MK+Bay | *** | 0.0001 |
|  |  |  |
| **pS556 ULK1/ULK1** |  |  |
| DMSO vs. MK | * | 0.0269 |
| MK vs. MK+Bay | * | 0.0232 |
|  |  |  |
| **OPA1 levels** |  |  |
| DMSO vs. MK | **** | <0.0001 |
| MK vs. MK+Bay | ** | 0.0037 |
|  |  |  |
| **HSP60 levels** |  |  |
| DMSO vs. MK | *** | 0.0004 |
| MK vs. MK+Bay | *** | 0.0001 |
|  |  |  |
| **OMI levels** |  |  |
| DMSO vs. MK | * | 0.0188 |
| MK vs. MK+Bay | * | 0.0109 |
|  |  |  |
| **COXIV levels** |  |  |
| DMSO vs. MK | * | 0.0447 |
| MK vs. MK+Bay | * | 0.0206 |
|  |  |  |
|  |  |  |
| **FigEV1K** |  |  |
| DMSO vs. MK8722 | * | 0.0164 |
| MK8722 vs. Bay-3827 | * | 0.0322 |
| MK8722 vs. MK8722+Bay-3827 | * | 0.0369 |
|  |  |  |
|  |  |  |
| **Figure 2** |  |  |
| **Figure 2B** |  |  |
| **pS80 ACC1/ACC1** |  |  |
| Vector DMSO vs Vector MK | **** | <0.0001 |
| FLAG-WT DMSO vs FLAG-WT MK | *** | 0.0001 |
| FLAG-Ile551Asn DMSO vs FLAG-Ile551Asn MK | **** | <0.0001 |
|  |  |  |
| **pS556 ULK1/ULK1** |  |  |
| Vector DMSO vs Vector MK | **** | <0.0001 |
| FLAG-WT DMSO vs FLAG-WT MK | **** | <0.0001 |
| FLAG-Ile551Asn DMSO vs FLAG-Ile551Asn MK | **** | <0.0001 |
|  |  |  |
| **OPA1 levels** |  |  |
| Vector DMSO vs Vector MK | **** | <0.0001 |
| Vector DMSO vs Vector Baf | ** | 0.0021 |
| Vector DMSO vs FLAG-WT DMSO | *** | 0.0007 |
| FLAG-WT DMSO vs FLAG-Ile551Asn DMSO | * | 0.018 |
| FLAG-Ile551Asn DMSO vs FLAG-Ile551Asn MK | **** | <0.0001 |
| FLAG-Ile551Asn DMSO vs FLAG-Ile551Asn Baf | *** | 0.0008 |
|  |  |  |
| **HSP60 levels** |  |  |
| Vector DMSO vs Vector MK | ** | 0.0024 |
| Vector DMSO vs Vector Baf | ** | 0.0013 |
| Vector DMSO vs FLAG-WT DMSO | ** | 0.003 |
| FLAG-WT DMSO vs FLAG-Ile551Asn DMSO | ** | 0.0051 |
| FLAG-Ile551Asn DMSO vs FLAG-Ile551Asn MK | *** | 0.0004 |
| FLAG-Ile551Asn DMSO vs FLAG-Ile551Asn Baf | *** | 0.001 |
|  |  |  |
| **COXIV levels** |  |  |
| Vector DMSO vs Vector MK | ** | 0.0011 |
| Vector DMSO vs Vector Baf | **** | <0.0001 |
| Vector DMSO vs FLAG-WT DMSO | **** | <0.0001 |
| FLAG-WT DMSO vs FLAG-Ile551Asn DMSO | *** | 0.0008 |
| FLAG-Ile551Asn DMSO vs FLAG-Ile551Asn MK | *** | 0.0007 |
| FLAG-Ile551Asn DMSO vs FLAG-Ile551Asn Baf | ** | 0.0058 |
|  |  |  |
| **NIX levels** |  |  |
| Vector DMSO vs Vector MK | *** | 0.0002 |
| Vector DMSO vs FLAG-WT DMSO | * | 0.0232 |
| FLAG-WT DMSO vs FLAG-Ile551Asn DMSO | *** | 0.0004 |
| FLAG-Ile551Asn DMSO vs FLAG-Ile551Asn MK | *** | 0.0006 |
|  |  |  |
|  |  |  |
| **Figure 2D** |  |  |
| Vector DMSO vs Vector MK | *** | 0.0001 |
| Vector DMSO vs FLAG-WT DMSO | **** | <0.0001 |
| FLAG-WT DMSO vs FLAG-WT MK | ns | 0.9434 |
| FLAG-WT DMSO vs FLAG-Ile551Asn DMSO | ** | 0.0021 |
| FLAG-Ile551Asn DMSO vs FLAG-Ile551Asn MK | ** | 0.0045 |
|  |  |  |
|  |  |  |
| **Figure 2E** |  |  |
| WT DMSO  vs. KO DMSO | * | 0.0263 |
| KO DMSO vs. KO MK | * | 0.0329 |
| KO MK vs. KO MK+Bay | * | 0.0376 |
|  |  |  |
| **Figure 2F** |  |  |
| WT DMSO  vs. KO DMSO | ** | 0.007 |
| KO DMSO vs. KO MK | ** | 0.0094 |
| KO MK vs. KO MK+Bay | ** | 0.0083 |
|  |  |  |
| **Figure 2G** |  |  |
| WT DMSO  vs. KO DMSO | ** | 0.0013 |
| KO DMSO vs. KO MK | ** | 0.007 |
| KO MK vs. KO MK+Bay | ** | 0.0026 |
|  |  |  |
| **Figure 2H** |  |  |
| WT DMSO  vs. KO DMSO | *** | 0.0006 |
| KO DMSO vs. KO MK | ** | 0.0057 |
| KO MK vs. KO MK+Bay | ** | 0.0017 |
|  |  |  |
|  |  |  |
| **Figure EV2** |  |  |
| **Figure EV2B** |  |  |
| Flag WT DMSO vs Flag WT MK | ns | 0.0983 |
| Flag WT DMSO vs Flag DMSO | ns | 0.1351 |
| Flag WT MK vs Flag MK | ns | 0.1472 |
| Flag DMSO vs Flag MK | ns | 0.0914 |
|  |  |  |
| **Figure EV2D** |  |  |
| **MT-CO1 levels** |  |  |
| Flag WT DMSO vs Flag WT MK | ns | 0.3263 |
| Flag WT DMSO vs Flag DMSO | * | 0.0125 |
| Flag DMSO vs Flag MK | * | 0.0135 |
|  |  |  |
| **TOM20 levels** |  |  |
| Flag WT DMSO vs Flag WT MK | ns | 0.0782 |
| Flag WT DMSO vs Flag DMSO | * | 0.0319 |
| Flag DMSO vs Flag MK | * | 0.0167 |
|  |  |  |
|  |  |  |
| **Figure EV2E** |  |  |
| WT DMSO vs. KO DMSO | ns | 0.1369 |
| WT DMSO  vs. KO MK | ns | 0.2485 |
| KO DMSO vs. KO MK | ns | 0.8866 |
|  |  |  |
| **Figure EV2F** |  |  |
| WT DMSO vs. KO DMSO | ns | 0.9766 |
| WT DMSO  vs. KO MK | ns | 0.7004 |
| KO DMSO vs. KO MK | ns | 0.8142 |
|  |  |  |
| **Figure EV2G** |  |  |
| WT DMSO vs. KO DMSO | ns | 0.7108 |
| WT DMSO  vs. KO MK | ns | 0.7816 |
| KO DMSO vs. KO MK | ns | 0.3582 |
|  |  |  |
| **Figure EV2H** |  |  |
| WT DMSO vs. KO DMSO | ns | 0.75 |
| WT DMSO  vs. KO MK | ns | 0.9224 |
| KO DMSO vs. KO MK | ns | 0.5342 |
|  |  |  |
|  |  |  |
| **Figure EV2I** |  |  |
| Flag WT DMSO vs Flag WT MK | ns | 0.8367 |
| Flag WT DMSO vs Flag DMSO | ns | 0.6657 |
| Flag DMSO vs Flag MK | * | 0.023 |
|  |  |  |
| **Figure EV2J** |  |  |
| Flag WT DMSO vs Flag WT MK | ns | 0.9449 |
| Flag WT DMSO vs Flag DMSO | ns | 0.8799 |
| Flag DMSO vs Flag MK | ns | 0.2733 |
|  |  |  |
| **Figure EV2K** |  |  |
| Flag WT DMSO vs Flag WT MK | ns | 0.6604 |
| Flag WT DMSO vs Flag DMSO | ns | 0.9125 |
| Flag DMSO vs Flag MK | ns | 0.1536 |
|  |  |  |
| **Figure EV2L** |  |  |
| Flag WT DMSO vs Flag WT MK | ns | 0.8774 |
| Flag WT DMSO vs Flag DMSO | ns | 0.6508 |
| Flag DMSO vs Flag MK | ns | 0.1591 |
|  |  |  |
| **Figure EV2M** |  |  |
| Flag WT DMSO vs Flag WT MK | ns | 0.7596 |
| Flag WT DMSO vs Flag DMSO | ns | 0.7512 |
| Flag DMSO vs Flag MK | ns | 0.4901 |
|  |  |  |
| **Figure EV2O** |  |  |
| Flag WT DMSO vs Flag WT MK | ns | 0.8914 |
| Flag WT DMSO vs Flag DMSO | * | 0.0113 |
| Flag DMSO vs Flag MK | * | 0.0369 |
|  |  |  |
| **Figure EV2P** |  |  |
| Flag WT DMSO vs Flag WT MK | ns | 0.3287 |
| Flag WT DMSO vs Flag DMSO | * | 0.0195 |
| Flag DMSO vs Flag MK | * | 0.0169 |
|  |  |  |
|  |  |  |
| **Figure 3** |  |  |
| **Figure 3B** |  |  |
| **OPA1 levels** |  |  |
| WT DMSO vs p.Arg435* DMSO | ** | 0.005 |
| WT DMSO vs p.Gln519* DMSO | * | 0.0174 |
| p.Arg435* DMSO vs p.Arg435* MK | *** | 0.0002 |
| p.Gln519* DMSO vs p.Gln519* MK | *** | 0.0003 |
|  |  |  |
| **OMI levels** |  |  |
| WT DMSO vs p.Arg435* DMSO | ** | 0.0039 |
| WT DMSO vs p.Gln519* DMSO | ** | 0.006 |
| p.Arg435* DMSO vs p.Arg435* MK | ** | 0.0043 |
| p.Gln519* DMSO vs p.Gln519* MK | ** | 0.002 |
|  |  |  |
| **COXIV levels** |  |  |
| WT DMSO vs p.Arg435* DMSO | ** | 0.0076 |
| WT DMSO vs p.Gln519* DMSO | * | 0.0232 |
| p.Arg435* DMSO vs p.Arg435* MK | ** | 0.0057 |
| p.Gln519* DMSO vs p.Gln519* MK | ** | 0.0017 |
|  |  |  |
| **NIX levels** |  |  |
| WT DMSO vs p.Arg435* DMSO | * | 0.0297 |
| WT DMSO vs p.Gln519* DMSO | * | 0.0243 |
| p.Arg435* DMSO vs p.Arg435* MK | ** | 0.0065 |
| p.Gln519* DMSO vs p.Gln519* MK | ** | 0.007 |
|  |  |  |
| **Figure 3D** |  |  |
| WT DMSO vs p.Arg435* DMSO | **** | <0.0001 |
| WT DMSO vs p.Gln519* DMSO | ** | 0.0017 |
| p.Arg435* DMSO vs p.Arg435* MK | **** | <0.0001 |
| p.Gln519* DMSO vs p.Gln519* MK | *** | 0.0003 |
|  |  |  |
|  |  |  |
| **Figure 4** |  |  |
| **Figure 4B** |  |  |
| **pS80 ACC1/ACC1** |  |  |
| Vehicle vs MK | * | 0.0222 |
| MLN vs MLN+MK | ** | 0.0075 |
|  |  |  |
| **pS556 ULK1/ULK1** |  |  |
| Vehicle vs MK | * | 0.0164 |
| MLN vs MLN+MK | * | 0.0226 |
|  |  |  |
| **NIX levels** |  |  |
| Vehicle vs MLN | **** | <0.0001 |
| Vehicle vs MLN+MK | **** | <0.0001 |
|  |  |  |
| **BNIP3 levels** |  |  |
| Vehicle vs MLN | ** | 0.0099 |
| Vehicle vs MLN+MK | *** | 0.0004 |
|  |  |  |
| **ATPB levels** |  |  |
| Vehicle vs MLN | *** | 0.0003 |
| MLN vs MLN+MK | ** | 0.0029 |
|  |  |  |
| **COXIV levels** |  |  |
| Vehicle vs MLN | ns | 0.0504 |
| MLN vs MLN+MK | *** | 0.0006 |
|  |  |  |
|  |  |  |
| **Figure 4D** |  |  |
| Vehicle vs. MK-8722 | ** | 0.0074 |
| Vehicle vs. MLN | * | 0.0351 |
| MLN vs. MLN+MK | ** | 0.0058 |
|  |  |  |
|  |  |  |
| **Figure 4F** |  |  |
| Vehicle vs Metformin | *** | 0.0003 |
|  |  |  |
| **Figure EV4** |  |  |
| **Figure EV4A** |  |  |
| MK -30 min (Pre) vs. +60 min (Post) | ** | 0.0046 |
| MLN4924 +60 min (Post) vs. MLN4924+MK-8722 +60 min (Post) | ** | 0.0032 |
| Vehicle +60 min (Post) vs. MK8722 +60 min (Post) | **** | <0.0001 |
|  |  |  |
| **Figure EV4B** |  |  |
| Metformin -30 min (Pre) vs. +60 min (Post) | * | 0.0275 |
| Vehicle +60 min (Post) vs. Metformin +60 min (Post) | ** | 0.0015 |
|  |  |  |
|  |  |  |
| **Figure EV4D** |  |  |
| **pS80 ACC1/ACC1** |  |  |
| Vehicle vs. Metformin | * | 0.0306 |
|  |  |  |
| **pS556 ULK1/ULK1** |  |  |
| Vehicle vs. Metformin | * | 0.0419 |
|  |  |  |
| **pS757 ULK1/ULK1** |  |  |
| Vehicle vs. Metformin | * | 0.0484 |
|  |  |  |
| **pT172 AMPK/AMPK** |  |  |
| Vehicle vs. Metformin | * | 0.0474 |
|  |  |  |
| **Figure 5** |  |  |
| **Figure 5C** |  |  |
| Vehicle vs. MLN | * | 0.0316 |
| MLN vs. MLN+MK | * | 0.0313 |
|  |  |  |
| **Figure 5E** |  |  |
| Vehicle vs. MLN | ns | 0.5253 |
| MLN vs. MLN+MK | *** | 0.001 |
|  |  |  |
|  |  |  |
| **Figure EV5** |  |  |
| **Figure EV5B** |  |  |
| **pS80 ACC1/ACC1** |  |  |
| Vehicle vs MK | *** | 0.0006 |
| MLN vs MLN+MK | **** | <0.0001 |
|  |  |  |
| **pS556 ULK1/ULK1** |  |  |
| Vehicle vs MK | *** | 0.0005 |
| MLN vs MLN+MK | **** | <0.0001 |
|  |  |  |
| **pS757 ULK1/ULK1** |  |  |
| Vehicle vs MK | ** | 0.0069 |
| MLN vs MLN+MK | ** | 0.0051 |
|  |  |  |
| **pT172 AMPK/AMPK** |  |  |
| Vehicle vs MK | * | 0.0229 |
| MLN vs MLN+MK | ns | 0.1642 |
|  |  |  |
| **NIX levels** |  |  |
| Vehicle vs MLN | *** | 0.0005 |
| Vehicle vs MLN+MK | **** | <0.0001 |
|  |  |  |
| **BNIP3 levels** |  |  |
| Vehicle vs MK | ns | 0.1094 |
| Vehicle vs MLN | ns | 0.4417 |
| Vehicle vs MLN+MK | ns | 0.7701 |
|  |  |  |
|  |  |  |
| **Figure EV5D** |  |  |
| Vehicle vs. MLN | ns | 0.2996 |
| MLN vs. MLN+MK | ns | 0.9315 |
