# Supplementary material for: Activation of AMPK as a therapeutic strategy for FBXL4-related mitochondrial DNA depletion syndrome
Source: EMBO Mol Med. 2026 Jun 17;18(8):3109–36. doi: 10.1038/s44321-026-00471-z (PMC13470155; doi:10.1038/s44321-026-00471-z)
Supplement: Supplementary file 1 [file 44321_2026_471_MOESM1_ESM.pdf]

EMBO Molecular Medicine

Activation of AMPK as a therapeutic strategy for FBXL4-related mitochondrial DNA depletion syndrome

Aniketh Bishnu, Juan Zapata-Munoz, Robert Taylor, Kei Sakamoto, and Ian Ganley

Corresponding author: Ian Ganley (i.ganley@dundee.ac.uk)

Review Timeline:

Submission Date:	18th Dec 25
Editorial Decision:	14th Jan 26
Revision Received:	23rd Apr 26
Editorial Decision:	19th May 26
Revision Received:	2nd Jun 26
Accepted:	3rd Jun 26

Editor: Zeljko Durdevic

Transaction Report:

(Note: With the exception of the correction of typographical or spelling errors that could be a source of ambiguity, letters and reports are not edited. Depending on transfer agreements, referee reports obtained elsewhere may or may not be included in this compilation. Referee reports are anonymous unless the Referee chooses to sign their reports.)

14th Jan 2026

Dear Prof. Ganley,

Thank you for the submission of your manuscript to EMBO Molecular Medicine. We have now received feedback from the three reviewers who agreed to evaluate your manuscript. All three referees recognize interest of the study but also raise important concerns that should be addressed in a major revision. If you would like to discuss further the points raised by the referees, I am available to do so via email or video. Let me know if you are interested in this option.

Further consideration of a revision that addresses reviewers' concerns in full will entail a second round of review. EMBO Molecular Medicine encourages a single round of revision only and therefore, acceptance or rejection of the manuscript will depend on the completeness of your responses included in the next, final version of the manuscript. For this reason, and to save you from any frustrations in the end, I would strongly advise against returning an incomplete revision.

We would welcome the submission of a revised version within three months for further consideration. Please let us know if you require longer to complete the revision.

I look forward to receiving your revised manuscript.

Yours sincerely,

Zeljko Durdevic

Zeljko Durdevic
Senior Editor
EMBO Molecular Medicine

*****
When preparing your revised manuscript, please refer to our guidelines: https://link.springer.com/journal/44321/submission-guidelines#cms-Revised-submissions. We perform an initial quality control of all revised manuscripts before re-review; failure to include requested items will delay the evaluation of your revision.

We require:

1) A .docx formatted version of the manuscript text (including legends for main figures, EV figures and tables). Please make sure that the changes are highlighted to be clearly visible.

2) Individual production quality figure files as .eps, .tif, .jpg (one file per figure). For guidance, download the 'Figure Guide PDF': https://media.springernature.com/original/springer-cms/rest/v1/content/27825798/data/v1.

3) A .docx formatted letter INCLUDING the reviewers' reports and your detailed point-by-point responses to their comments. As part of the EMBO Press transparent editorial process, the point-by-point response is part of the Review Process File (RPF), which will be published alongside your paper.

4) A complete author checklist, which you can download from our author guidelines. Please insert information in the checklist that is also reflected in the manuscript. The completed author checklist will also be part of the RPF.

5) Please note that all corresponding authors are required to supply an ORCID ID for their name upon submission of a revised manuscript.

6) It is mandatory to include a 'Data Availability' section after the Materials and Methods. Before submitting your revision, primary datasets produced in this study need to be deposited in an appropriate public database, and the accession numbers and database listed under 'Data Availability'. Please remember to provide a reviewer password if the datasets are not yet public.

In case you have no data that requires deposition in a public database, please state so in this section. Note that the Data Availability Section is restricted to new primary data that are part of this study.

7) For data quantification: please specify the name of the statistical test used to generate error bars and P values, the number (n) of independent experiments (specify technical or biological replicates) underlying each data point and the test used to calculate p-values in each figure legend. The figure legends should contain a basic description of n, P and the test applied. Graphs must include a description of the bars and the error bars (s.d., s.e.m.).

8) At EMBO Press we ask authors to provide source data for the main manuscript figures. You will receive a separate email with instructions for providing source data with your revised manuscript, including how to upload and organize the files.

9) Our journal encourages inclusion of *data citations in the reference list* to directly cite datasets that were re-used and obtained from public databases. Data citations in the article text are distinct from normal bibliographical citations and should directly link to the database records from which the data can be accessed. In the main text, data citations are formatted as follows: "Data ref: Smith et al, 2001" or "Data ref: NCBI Sequence Read Archive PRJNA342805, 2017". In the Reference list, data citations must be labeled with "[DATASET]". A data reference must provide the database name, accession number/identifiers and a resolvable link to the landing page from which the data can be accessed at the end of the reference.

10) We replaced Supplementary Information with Expanded View (EV) Figures and Tables that are collapsible/expandable online. A maximum of 5 EV Figures can be typeset. EV Figures should be cited as 'Figure EV1, Figure EV2" etc... in the text and their respective legends should be included in the main text after the legends of regular figures.

- For the figures that you do NOT wish to display as Expanded View figures, they should be bundled together with their legends in a single PDF file called *Appendix*, which should start with a short Table of Content. Appendix figures should be referred to in the main text as: "Appendix Figure S1, Appendix Figure S2" etc.

- Additional Tables/Datasets should be labeled and referred to as Table EV1, Dataset EV1, etc. Legends have to be provided in a separate tab in case of .xls files. Alternatively, the legend can be supplied as a separate text file (README) and zipped together with the Table/Dataset file.

11) The paper explained: EMBO Molecular Medicine articles are accompanied by a summary of the articles to emphasize the major findings in the paper and their medical implications for the non-specialist reader. Please provide a draft summary of your article highlighting

- the medical issue you are addressing,

- the results obtained and

- their clinical impact.

This may be edited to ensure that readers understand the significance and context of the research. Please refer to any of our published articles for an example.

12) Author contributions: You will be asked to provide CRediT (Contributor Role Taxonomy) terms in the submission system. These replace a narrative author contribution section in the manuscript.

13) A Conflict of Interest statement should be provided in the main text.

14) Every published paper includes a 'Synopsis' to further enhance discoverability. Synopses are displayed on the journal webpage and are freely accessible to all readers. They include a short stand first (maximum of 300 characters, including space) as well as 2-5 one-sentences bullet points that summarizes the paper. Please write the bullet points to summarize the key NEW findings. They should be designed to be complementary to the abstract - i.e. not repeat the same text. We encourage inclusion of key acronyms and quantitative information (maximum of 30 words / bullet point). Please use the passive voice. Please attach these in a separate file or send them by email, we will incorporate them accordingly.

Please also suggest a striking image or visual abstract to illustrate your article as a PNG file 550 px wide x 300-600 px high.

15) Include a Reagents and Tools Table as part of the Methods section, which can be downloaded from our author guidelines.

EMBO Molecular Medicine has a "scooping protection" policy, whereby similar findings that are published by others during review or revision are not a criterion for rejection. Should you decide to submit a revised version, I do ask that you get in touch after three months if you have not completed it, to update us on the status.

***** Reviewer's comments *****

Referee #1 (Comments on Novelty/Model System for Author):

This is a follow up study based on a previous publication by the same research group (Longo et al., 2024). In that respect, the proposed study is not novel, although this is the first time to my knowledge that MK-8722 is tested in models of FBXL4 inhibition/KO and in the context of mitochondrial quality control.

Referee #1 (Remarks for Author):

This is a very interesting paper that highlights the importance of balanced mitochondrial turnover in a specific disease model associated to mitochondrial DNA depletion syndrome. In recent years, it became clear that mitophagy is required not only to get rid of dysfunctional mitochondria under stressful condition, but it also occurs under basal condition to maintain the mitochondrial repertoire stable. While many progresses have been made in the dissection of the molecular mechanisms underlying stress-induced mitophagy (executed by PINK1/Parkin), we do know a lot less about so-called basal mitophagy, which is very relevant in differentiating tissues, but can become pathological if overactivated.
This work adds new interesting insights into AMPK activation as approach to inhibit NIX/BNIP3-dependent basal mitophagy, and its potential importance as therapeutic target for devastating mitochondrial disease, MTDPS13 syndrome, caused by mutations in the mitochondrial E3 Ub ligase FBXL4.
Methods are robust and data are convincing, we do have however few suggestions to increase relevance for broader audience.

-Levels of pleiotropic mitochondrial protein OPA1 are increased upon AMPK activation, also in FBXL4 KO background, and in fibroblasts from patients. OPA1 is a pro-survival protein, which controls fusion of inner mitochondrial membrane. It is also a master regulator of mitochondrial-dependent cell death (via regulation of the mitochondrial cristae junctions), and it controls the assembly of the respiratory supercomplexes for efficient mitochondrial respiration (seminal works of Luca Scorrano and José Antonio Enríquez, among many others). What is the role of pro-survival protein OPA1 in the context of the protective effect of AMPK activation proposed by the authors? Did the authors test the levels of OPA1 when cells are co-treated with MK and BAY? It is possible that the effect on mitophagy is secondary to the effect that AMPK activation exerts on pro-survival protein OPA1. Ultrastructural studies by EM as well as analysis of supercomplexes assembly and activity are strongly advised. Experiments performed in Fig.EV3A-D do not fully address this important issue.

- The evaluation of the levels of mitochondrial proteins as read out of mitochondrial mass is not ideal, unless this is performed upon assessment of the total mitochondrial proteome (i.e. mass spec approach). In fact, the evaluation of steady state levels of two/three mitochondrial resident proteins is not indicative of the mitochondrial mass, as this depends on protein turnover, which might be different from organelle turnover. HSP60 and COXIV are often used as conventional indicators of mitochondrial content but I recommend being more cautious about directly linking mitochondrial protein levels with organelle mass and/or mitophagy.

- It would be very interesting to assess mitochondrial biogenesis in parallel to mitophagy, specifically when the two drugs are used in combination, and in the KO model. The restoration of mitochondrial protein might be the result of decreased degradation or enhanced biogenesis (or both). Does AMPK activation enhance mitochondrial biogenesis in the proposed pathological model?

-Additional information on the pharmacokinetics properties of MK-8722 and metformin would be very helpful. Do they cross the BBB? Are they orally bioavailable? Half life in plasma, liver, brain? Stability in the presence of liver microsomes?

-This study is a follow up of a previous publication (Longo et al, 2024), which unveils the dual role of AMPK activation in mitochondrial turnover. AMPK activation promotes PINK1/Parkin dependent degradation of dysfunctional mitochondria, while in parallel seems to enhance mitochondrial turnover, by activating mitochondrial biogenesis and by inhibiting NIX/BNIP3-dependent basal mitophagy. The paper would benefit from a broader description of the AMPK literature in the context of autophagy/mitophagy and mitochondrial quality control. The role of AMPK in mitochondrial biogenesis is only briefly mentioned in the introduction, but it seems very relevant in this context. Is it known the molecular mechanism of biogenesis activation? The effect of AMPK activation on general macroautophagy (i.e. block of autophagy) is limited to mitophagy or other selective types of autophagy are affected? Could this be relevant in the pathology of MTDPS13 syndrome?

Referee #2 (Comments on Novelty/Model System for Author):

This is a concise and very interesting study that identifies AMPK activation as a potential therapeutic strategy for Mitochondrial DNA Depletion Syndrome 13 (MTDPS13), a rare, inborn error of metabolism caused by mutations in the FBXL4 gene in humans. Building on previous work that demonstrated that AMPK activation can inhibit receptor-mediated mitophagy through the sequestration of ULK1, the authors demonstrate that pharmacological activation of AMPK through multiple independent compounds can rescue the elevated mitophagy in FBXL4-deficient cells and patient fibroblasts, as well as in a chemically-induced in vivo model of FBXL4-deficiency.

Referee #2 (Remarks for Author):

This is a concise and very interesting study that identifies AMPK activation as a potential therapeutic strategy for Mitochondrial DNA Depletion Syndrome 13 (MTDPS13), a rare, inborn error of metabolism caused by mutations in the FBXL4 gene in humans. Building on previous work that demonstrated that AMPK activation can inhibit receptor-mediated mitophagy through the sequestration of ULK1, the authors demonstrate that pharmacological activation of AMPK through multiple independent compounds can rescue the elevated mitophagy in FBXL4-deficient cells and patient fibroblasts, as well as in a chemically-induced in vivo model of FBXL4-deficiency.

The study is well articulated, logically sound, and utilizes a breadth of models to orthogonally test hypotheses. The experiments are largely consistent with the models put forth by the authors, yet there may be alternative explanations to some of the proposed models that could be explored to gain further insights into the mechanisms underlying the rescue seen in FBXL4-deficient models. Furthermore, the study would be enhanced by the inclusion of a few additional controls and literature citations, which are outlined below. Overall, this was a very enjoyable paper to read, and is an important advance for patients with MTDPS13, for which there are no currently available treatments.

1. The authors state that their previous work demonstrated that AMPK activation inhibits NIX-mediated mitophagy, and throughout the paper, reference only NIX-mediated mitophagy in reference to FBXL4-deficient models (e.g., end of paragraph 1, page 3; second paragraph, page 4, etc). Furthermore, multiple figures test the upregulation of NIX alone and in the absence of any study of BNIP3. This is not well justified, as recent work on FBXL4 (PMIDs: 36896912, 37161784, 37102372) demonstrates that both mitophagy receptors are upregulated upon pharmacological or genetic inactivation of FBXL4. The authors should amend appropriate statements within the text to include both BNIP3 and NIX, or describe why only one receptor is described in the absence of the other. Furthermore, Figures 1A, EV1A, 2A, EV2A, 4A, and 5A should include BNIP3 blots in the associated conditions. This is particularly important for the study in liver tissue, as there is limited literature linking NIX to liver function. In contrast, BNIP3 is well established as a regulator of lipid metabolism and hepatic homeostasis (PMID: 22547685, PMID: 33459136, PMID: 19147804, PMID: 38615890 etc.). Assessing BNIP3 with similar rigor would greatly strengthen this section.

2. The authors perform an experiment to test whether "the mitochondria restored upon AMPK-mediate mitophagy inhibition are functional." The authors demonstrate that AMPK activation indeed increases oxygen consumption across all tested conditions in FBXL4 KO cells, which is consistent with this model. However, the authors also note that AMPK activation can induce mitochondrial biogenesis, which confounds the interpretation of this experiment, as AMPK activation could either: 1. Rescue mitophagy with functional organelles, 2. Promote biogenesis of new functional organelles, or 3. A combination of both. Any of these models would be interesting. However, the current experimentation does not support the authors' conclusions that AMPK promotes the "preferential degradation of functional mitochondria," nor does it support that the actual mitochondria that are "rescued" from degradation were functional. The authors could perform additional experimentation to distinguish the aforementioned models (perhaps by disrupting the biogenesis machinery in the presence of pharmacological activation of AMPK), or, instead, could modify their claims to include a greater emphasis on the potential role of mitochondrial biogenesis and decrease the strength of their claims that AMPK preferentially degrades functional mitochondria.

3. Some of the representative blots do not seem to match the densitometry shown. For instance, in Figure 3, OMI levels do not seem to be rescued to WT levels by MK-8722 in the representative blot, but are reported to be very similar levels in the densitometry analysis in 3B. Similarly, in EV2A, HSP60 levels are barely detectable in lanes 3+4 of the blot shown, though the densitometry analysis shows approximately equal expression to untreated FBXL4 KO cells. Please comment on this apparent discrepancy and/or select a different blot that is more representative of the quantification shown.

4. In multiple figures (in particular, 1A, 1C, EV1A, 2A, EV2A), NIX seems to have an upward shift in MK-8722-treated cells. As NIX has been demonstrated to be phosphorylated (PMID: 28442745, 37833277), the authors could expand upon whether MK-8722 promotes accumulation of post-translationally modified forms of these proteins, and whether evidence exists that this could be direct regulation. This expansion can either be postulated or shown experimentally.

5. Figures would be strengthened by including statistical comparisons across all conditions instead of only selected comparisons that are reported.

6. Some statements could be interpreted as overstated and should be reworded. For instance, when describing data in Figures 2A-B (page 7) and 3A (page 9), the authors claim restoration of "mitochondrial proteins" but only demonstrate this for a handful of targets. Addition of "tested" or "select" to describe these data would be more appropriate. On page 7, the authors claim that

"loss of FBXL4 catalytic activity is responsible for the observed increase in mitophagy," however, there is little evidence to support the claim that this mutation disrupts catalytic activity. The authors could address this either experimentally or by referencing previously published data. Finally, as noted by the authors, MLN4924 is a broad-spectrum cullin-RING ligase inhibitor that targets more F-box proteins than just FBXL4. The use of terms such as "FBXL4 inhibition" (including in Figure 5) should therefore be revised to "SCF inhibition".

7. Some literature citations could be included such as some on MTDPS13 patient symptom presentation (PMID: 23993193, 23993194, 30804983, 25868664) and recent evidence that FBXL4 works in concert with PPTC7 to facilitate BNIP3/NIX turnover (PMID: 38151018, 38992176, 37833277).

Referee #3 (Remarks for Author):

Rare FBXL4 mutations lead to a mitochondrial depletion syndrome for which there is no rational treatment option. The typical life expectancy is about 3 years old. In 2023 several labs robustly showed that the relevant mechanism involves FBXL4-mediated degradation of the mitophagy adaptors BNIP3 and BNIP3L/NIX. In FBXL4 syndrome patients these factors are elevated and the resultant enhanced mitochondrial turnover leads to depletion of mitochondria. Thus interfering with this mitophagy pathway could restore mitochondrial mass. Here, the Ganley group build on their published observations that AMPK activators inhibit BNIP3/NIX mitophagy by sequestering ULK1, whilst the PINK/Parkin pathway (which senses damaged mitochondria remains intact). They have now shown that this approach can be used to replenish mitochondria in patient derived cells and in an animal model which recapitulates some aspects of the disease. I think this offers a real possibility of repurposing drugs towards an experimental therapy for children, as any potential negative effects of the drug are offset by the severity of the disease. Thus I am enthusiastic about the manuscript in general and consider the technical quality to be high.

General comments:

I feel that the following points need a bit more discussion.

sequestration of ULK1 is likely to result in inhibition of multiple autophagy pathways - the PINK1/Parkin pathway may be an exception.
The mouse model for chemical depletion uses MLN2924 which will inhibit all Cullin E3 ligases including FBXL4. For most of them this does not matter, but VHL has also been shown to be a regulator of BNIP3 and NIX and a strong suppressor of mitophagy. So the effect on mitochondria in the mouse will likely reflect a combination of these two E3s rather than just FBXL4 as occurs in patients- this needs to be made clearer. Nevertheless the reversal by MK is impressive.
Figure 3, shows a couple of patient derived fibroblast lines with enhanced mitophagy compared with a single wild type line. What is never quite clear is what is the variation between wild type individuals.
At some points there is an emphasis on NIX rather than BNIP3- this needs some explanation.

Referee #1 (Remarks for Author):

This is a very interesting paper that highlights the importance of balanced mitochondrial turnover in a specific disease model associated to mitochondrial DNA depletion syndrome. In recent years, it became clear that mitophagy is required not only to get rid of dysfunctional mitochondria under stressful condition, but it also occurs under basal condition to maintain the mitochondrial repertoire stable. While many progresses have been made in the dissection of the molecular mechanisms underlying stress-induced mitophagy (executed by PINK1/Parkin), we do know a lot less about so-called basal mitophagy, which is very relevant in differentiating tissues, but can become pathological if overactivated.

This work adds new interesting insights into AMPK activation as approach to inhibit NIX/BNIP3-dependent basal mitophagy, and its potential importance as therapeutic target for devastating mitochondrial disease, MTDPS13 syndrome, caused by mutations in the mitochondrial E3 Ub ligase FBXL4.

Methods are robust and data are convincing, we do have however few suggestions to increase relevance for broader audience.

Response: We thank the Reviewer for taking time to review the manuscript and provide positive and constructive feedback.

-Levels of pleiotropic mitochondrial protein OPA1 are increased upon AMPK activation, also in FBXL4 KO background, and in fibroblasts from patients. OPA1 is a pro-survival protein, which controls fusion of inner mitochondrial membrane. It is also a master regulator of mitochondrial-dependent cell death (via regulation of the mitochondrial cristae junctions), and it controls the assembly of the respiratory supercomplexes for efficient mitochondrial respiration (seminal works of Luca Scorrano and José Antonio Enríquez, among many others).

What is the role of pro-survival protein OPA1 in the context of the protective effect of AMPK activation proposed by the authors?

Response: We thank the reviewer for the important points regarding OPA1, for which we had not considered. We now include additional experimentation to investigate this further.

Did the authors test the levels of OPA1 when cells are co-treated with MK and BAY?

Response: Yes, the level of OPA1 was tested in FBXL4 KO cells co-treated with MK-8722+BAY-3827 (Fig.EV1I) in the original submission. OPA1 levels show a similar change to the other mitochondrial proteins blotted for, i.e. it increases upon MK-8722 treatment, which is reduced upon cotreatment with BAY-3827 (as it inhibits MK-8722-mediated AMPK activation).

It is possible that the effect on mitophagy is secondary to the effect that AMPK activation exerts on pro-survival protein OPA1. Ultrastructural studies by EM as well as analysis of supercomplexes assembly and activity are strongly advised.

Experiments performed in Fig.EV3A-D do not fully address this important issue.

Response: Thank you for raising this important point. As per the suggestion, we have analysed supercomplex assembly by Blue-Native PAGE of crude mitochondrial fraction lysed with Digitonin. Mitochondrial complexes were detected by immunoblotting with MT-CO1 (Complex IV) antibody. The ratio of MT-CO1 supercomplex (MITO-SC, higher molecular weight bands) to MT-CO1 complex (lower molecular weight band) was used to estimate supercomplex assembly. Our data suggest that there is no significant change in supercomplex assembly in FBXL4 KO or WT cells post AMPK activation. However, we do note the FBXL4 KO cells have a lower amount of MT-CO1, which increases upon AMPK activation. This was confirmed by conventional SDS-PAGE and immunoblotting. This increase matches TOM20 in the same blot and is consistent with all other mitochondrial markers tested in the manuscript. Therefore, at least under the conditions tested, we think that increased supercomplex assemble is not a major contributor to the observed effects of AMPK activation. The new data is shown below and added to the manuscript (Fig EV2A-D). The new text describing this is on page 10 (highlighted in track-changes manuscript version).

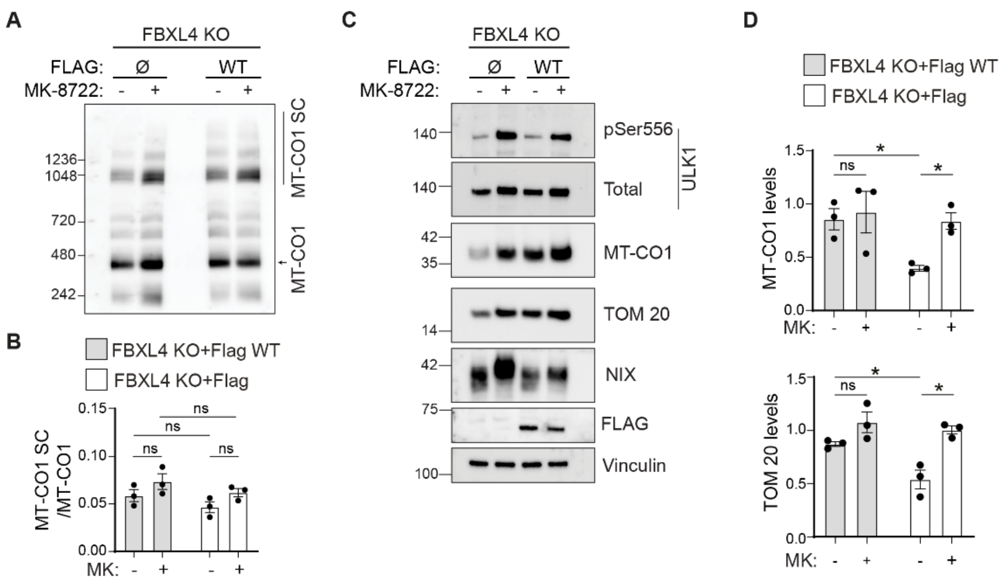

Unfortunately, due to technical difficulties, we didn’t manage to perform TEM but have performed super resolution microscopy (Airyscan 100X objective with 8X digital zoom) of mitochondria in FBXL4 KO cells treated with either DMSO or MK-822 and did not observe any significant differences. The data are shown below for the reviewer. The outer

mitochondrial membrane is tagged with EGFP-FIS1 while the inner mitochondrial membrane is immune-stained with COXIV (Red).

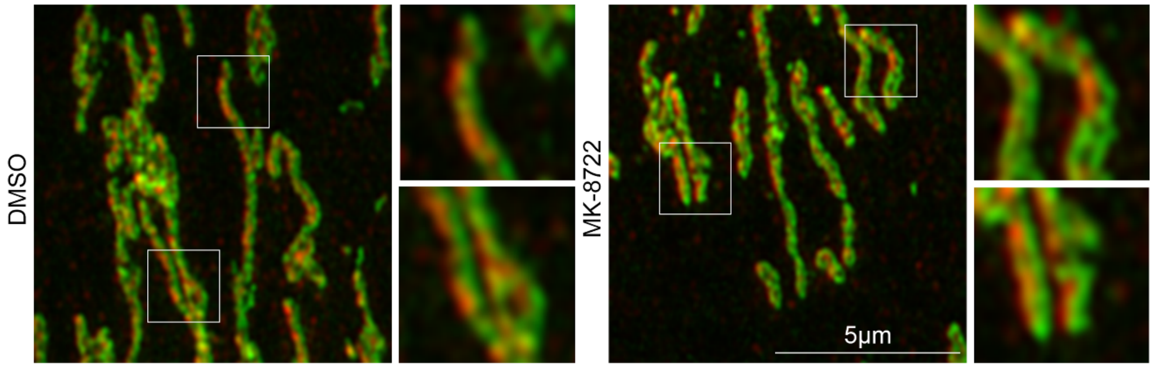

- The evaluation of the levels of mitochondrial proteins as read out of mitochondrial mass is not ideal, unless this is performed upon assessment of the total mitochondrial proteome (i.e. mass spec approach). In fact, the evaluation of steady state levels of two/three mitochondrial resident proteins is not indicative of the mitochondrial mass, as this depends on protein turnover, which might be different from organelle turnover. HSP60 and COXIV are often used as conventional indicators of mitochondrial content but I recommend being more cautious about directly linking mitochondrial protein levels with organelle mass and/or mitophagy.

Response: We appreciate and agree with the Reviewer's comment. Originally, we had tested five different mitochondrial proteins across various cell and mouse models (OPA1, HSP60, OMI, COXIV and ATPB), and to further strengthen the argument for mitochondrial content in general, we have added data for three more proteins (MT-CO1, TOM20 and TFAM). The levels of all these mitochondrial proteins tested show the same pattern (reduced in FBXL4 KO, which increase with AMPK activation). The new MT-CO1 and TOM20 blots are shown above and in new Fig.EV2C-D) and the TFAM blot is shown below and in new Fig.EV2N-O). As a non-protein-based readout of mitochondrial mass we have quantified the level of mitochondrial DNA (mtDNA) by qPCR. We observed a significant decrease in mtDNA in FBXL4 KO cells compared to the WT rescue cells. AMPK activation with MK-8722 restored the level of mtDNA to a level similar to WT cells (new Fig.EV2P). Additionally, we have also modified our statement to "AMPK activation restores the level of all the evaluated mitochondrial proteins".

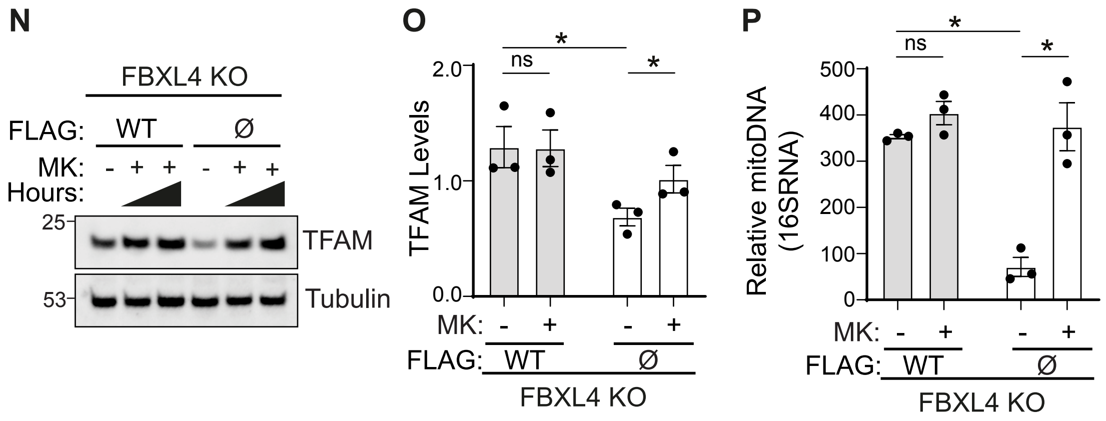

- It would be very interesting to assess mitochondrial biogenesis in parallel to mitophagy, specifically when the two drugs are used in combination, and in the KO model. The restoration of mitochondrial protein might be the result of decreased degradation or enhanced biogenesis (or both). Does AMPK activation enhance mitochondrial biogenesis in the proposed pathological model?

Response: This is a key point raised by this (and the other) Reviewer. As per suggestions, we have assessed mitochondrial biogenesis in our FBXL4 KO and WT rescue cells post MK-8722 treatment. Our data show (see below and in new Fig.EV2I-L) that AMPK significantly increases *PGC1α* transcript levels in FBXL4 KO cells (and to a lesser extent in WT cells). However, we did not observe any significant change in transcripts of mitochondrial proteins, including COXIV, CPT1a, ATPB and TFAM, upon AMPK activation in FBXL4 KO and WT cells. However, as mentioned above, in this time frame we see significant changes in TFAM protein levels and in mtDNA content. We interpret this as the block in mitophagy happening first, and is the primary reason we see increased mitochondrial markers following AMPK activation. Mitochondrial biogenesis is nevertheless initiated and is likely to contribute following prolonged AMPK activation. This is described in more detail in the additional text at the bottom of page 10 in the main manuscript.

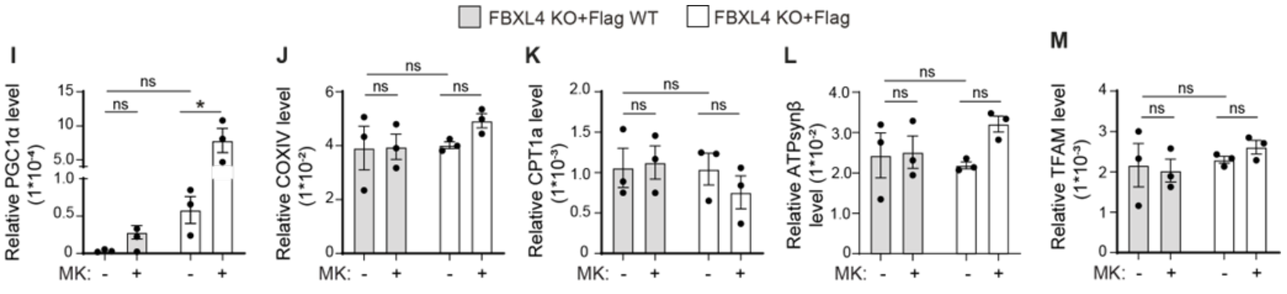

We have not tested the effect of BAY-3827 on the MK-8722-induced gene transcription here, as recently published RNA-sequencing data (from the group of co-author Kei

Sakamoto) demonstrated that BAY-3827 inhibits MK-8722-mediated gene expression in AMPK WT, but not in AMPK α1/2 double knockout, hepatocytes. One of the most significantly modulated gene in this data set was *PGC1α* (Fraguas Bringas et al. 2025).

-Additional information on the pharmacokinetics properties of MK-8722 and metformin would be very helpful. Do they cross the BBB? Are they orally bioavailable? Half life in plasma, liver, brain? Stability in the presence of liver microsomes? Response: Some of these data have already been published, as noted in the Discussion. As stated in the Discussion MK-8722 has not entered clinical trials, hence much of this information is lacking. We are currently in discussions with a pharmaceutical company to utilise PLX-770 in MTDPS13 patients. For the Reviewer, MK-8722 is an orally bioavailable (~82% in mouse) and has profound tissue penetrance across multiple tissues indicated by increased AMPK substrate phosphorylation. Previously it was reported that the unbound fraction of MK-8722 in the plasma is ~0.1%. Half-life in plasma is ~2.5 hrs. To the best of our knowledge the tissue specific half-life is not investigated/reported. The ability of MK-8722 to cross the BBB has not been reported (however we do see effects in this study, which may or may not be indirect). Given the systemic pan-activation of MK-8722 in can considered stable in presence of liver microsomes (Myers et al. 2017). Metformin is orally bioavailable (~50-60% in humans) and first-line therapy for type 2 diabetes. The half-life of metformin in the plasma ranges from ~1.7-4.5 hours in humans. Using C11-labelled metformin in human, it has been demonstrated that metformin is predominantly taken up by liver, intestine and kidney by organic cation transporters including OCT1 (Sundelin et al. 2020). Metformin is not metabolised and excreted in the urine (Graham et al. 2011).

-This study is a follow up of a previous publication (Longo et al, 2024), which unveils the dual role of AMPK activation in mitochondrial turnover. AMPK activation promotes PINK1/Parkin dependent degradation of dysfunctional mitochondria, while in parallel seems to enhance mitochondrial turnover, by activating mitochondrial biogenesis and by inhibiting NIX/BNIP3-dependent basal mitophagy. The paper would benefit from a broader description of the AMPK literature in the context of autophagy/mitophagy and mitochondrial quality control. The role of AMPK in mitochondrial biogenesis is only briefly mentioned in the introduction, but it seems very relevant in this context.

Is it known the molecular mechanism of biogenesis activation?

Response: We apologise for the brevity in the Introduction but the role that AMPK plays in regulation of autophagy/mitophagy is complex and context dependent – as we have shown in the cited Longo et al., 2024 paper. We have now briefly expanded on the

mechanism of how AMPK enhances mitochondrial biogenesis. The text is shown below and is on page three of the main manuscript:

"Of relevance, AMPK activation can also positively regulate mitochondrial biogenesis, in large part via upregulation of the transcriptional co-activator, peroxisome proliferator-activated receptor gamma coactivator 1α (PGC1α) – a master regulator of mitochondrial biogenesis (Handschin and Spiegelman 2006). AMPK achieves this via multiple mechanisms including phosphorylation of Folliculin-Interacting Protein 1, which results in nuclear translocation of transcription factor-EB (TFEB) and/or transcription factor binding to IGHM enhancer 3 (TFE3), which in turn leads to increased PGC1α levels and mitochondrial biogenesis (Malik et al. 2023)."

The effect of AMPK activation on general macroautophagy (i.e. block of autophagy) is limited to mitophagy or other selective types of autophagy are affected?

Could this be relevant in the pathology of MTDPS13 syndrome?

Response: This is an intriguing point and certainly could be relevant. We have previously demonstrated that NIX also regulates pexophagy under hypoxic-like conditions (Wilhelm et al. 2022). We have also shown that AMPK activation blocks this form of pexophagy in ARPE-19 cells (Longo et al. 2024). To look and see if the increased NIX/BNIP3 levels caused by loss of FBXL4 could also be affecting pexophagy, we looked at the level of two peroxisomal proteins in WT and FBXL4 KO cells (-/+ MK-8722). In contrast to mitochondrial proteins, we found no change in catalase or PMP70 (which we have previously shown to decrease upon NIX-mediated pexophagy). These data are shown in below and in new Fig. EV1B-C. This suggests that NIX/BNIP3 on peroxisomes are regulated in a different manner (at least in ARPE19 cells). This is discussed at the bottom of page 6 in the main text of manuscript.

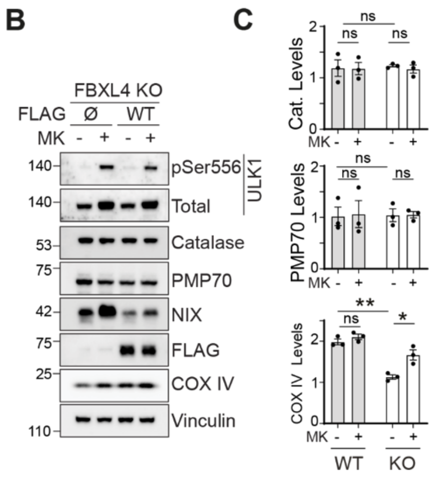

Referee #2 (Remarks for Author):

This is a concise and very interesting study that identifies AMPK activation as a potential therapeutic strategy for Mitochondrial DNA Depletion Syndrome 13 (MTDPS13), a rare, inborn error of metabolism caused by mutations in the FBXL4 gene in humans. Building on previous work that demonstrated that AMPK activation can inhibit receptor-mediated mitophagy through the sequestration of ULK1, the authors demonstrate that pharmacological activation of AMPK through multiple independent compounds can rescue the elevated mitophagy in FBXL4-deficient cells and patient fibroblasts, as well as in a chemically-induced in vivo model of FBXL4-deficiency.

The study is well articulated, logically sound, and utilizes a breadth of models to orthogonally test hypotheses. The experiments are largely consistent with the models put forth by the authors, yet there may be alternative explanations to some of the proposed models that could be explored to gain further insights into the mechanisms underlying the rescue seen in FBXL4-deficient models. Furthermore, the study would be enhanced by the inclusion of a few additional controls and literature citations, which are outlined below. Overall, this was a very enjoyable paper to read, and is an important advance for patients with MTDPS13, for which there are no currently available treatments.

Response: We thank the Reviewer for their time, positive feedback, and helpful comments.

1. The authors state that their previous work demonstrated that AMPK activation inhibits NIX-mediated mitophagy, and throughout the paper, reference only NIX-mediated mitophagy in reference to FBXL4-deficient models (e.g., end of paragraph 1, page 3; second paragraph, page 4, etc). Furthermore, multiple figures test the upregulation of NIX alone and in the absence of any study of BNIP3. This is not well justified, as recent work on FBXL4 (PMIDs: 36896912, 37161784, 37102372) demonstrates that both mitophagy receptors are upregulated upon pharmacological or genetic inactivation of FBXL4. The authors should amend appropriate statements within the text to include both BNIP3 and NIX, or describe why only one receptor is described in the absence of the other. Furthermore, Figures 1A, EV1A, 2A, EV2A, 4A, and 5A should include BNIP3 blots in the associated conditions. This is particularly important for the study in liver tissue, as there is limited literature linking NIX to liver function. In contrast, BNIP3 is well established as a regulator of lipid metabolism and hepatic homeostasis (PMID: 22547685, PMID: 33459136, PMID: 19147804, PMID: 38615890 etc.). Assessing BNIP3 with similar rigor would greatly strengthen this section.

Response: Many thanks for raising this point and we apologise for the omission, as we had not purposely meant to say all the effects were NIX and not BNIP3-dependent. To avoid any confusion, we have now also included the requested BNIP3 blots and amended

the statements to include both NIX and BNIP3. In line with this, the modulation of BNIP3 levels in FBXL4 KO cells both pre and post MK-8722 treatment mirrors the change in NIX. Of note, we did not manage to provide BNIP3 blots for Fig.EV1H (FBXL4 KO treated with MK-8722 and Bay-3827), as we no longer had cell lysates.

For the liver tissue we have now included the BNIP3 data and observed a significant increase in BNIP3 in animals receiving MLN-4924 and/or MK-8722 treatment. This is shown in new Fig.4A-B and the relevant BNIP3 data is shown below:

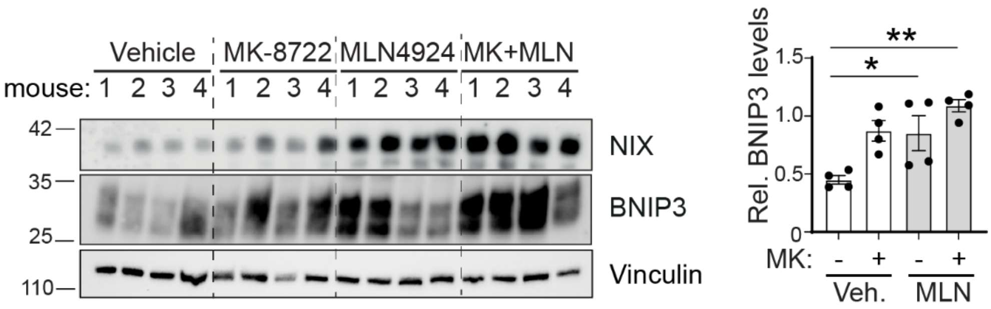

To more definitively analyse the contribution of NIX and BNIP3 to mitophagy in our ARPE19 cells, we created NIX and BNIP3 single and DKO cells. Upon induction of mitophagy with MLN4924, we found that loss of NIX had a greater impact on mitophagy compared to BNIP3, with almost complete mitophagy loss in the DKO cells. Therefore, NIX is the major regulator of mitophagy in ARPE19 cells, being only partially redundant with BNIP3. These new data are shown below and in new Fig.EV1A.

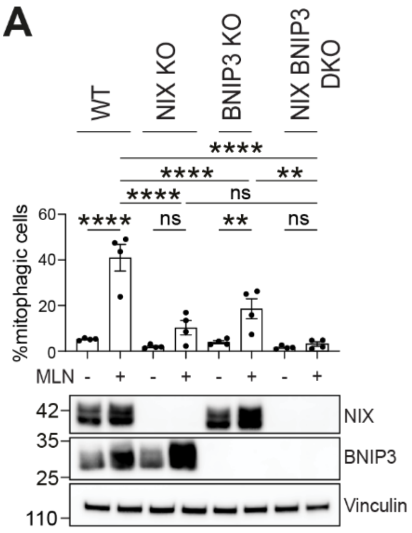

2. The authors perform an experiment to test whether "the mitochondria restored upon AMPK-mediate mitophagy inhibition are functional." The authors demonstrate that AMPK

activation indeed increases oxygen consumption across all tested conditions in FBXL4 KO cells, which is consistent with this model. However, the authors also note that AMPK activation can induce mitochondrial biogenesis, which confounds the interpretation of this experiment, as AMPK activation could either: 1. Rescue mitophagy with functional organelles, 2. Promote biogenesis of new functional organelles, or 3. A combination of both. Any of these models would be interesting. However, the current experimentation does not support the authors' conclusions that AMPK promotes the "preferential degradation of functional mitochondria," nor does it support that the actual mitochondria that are "rescued" from degradation were functional. The authors could perform additional experimentation to distinguish the aforementioned models (perhaps by disrupting the biogenesis machinery in the presence of pharmacological activation of AMPK), or, instead, could modify their claims to include a greater emphasis on the potential role of mitochondrial biogenesis and decrease the strength of their claims that AMPK preferentially degrades functional mitochondria.

Response: The Reviewer indeed raises a valid point with respect to mitochondrial biogenesis (which was also pointed out by the other reviewers). We copy the Response to Reviewer 1 here: Our data show (see below and in new Fig.EV2I-L) that AMPK significantly increases *PGC1a* transcript levels in FBXL4 KO cells (and to a lesser extent in WT cells). However, we did not observe any significant change in transcripts of mitochondrial proteins, including COXIV, CPT1a, ATPB and TFAM, upon AMPK activation in FBXL4 KO and WT cells. However, as mentioned above (and shown below), in this time frame we see significant changes in TFAM protein levels and in mtDNA content. We take this to mean the block in mitophagy happens first and is the prime reason we see increased mitochondrial markers following AMPK activation. Mitochondrial biogenesis is

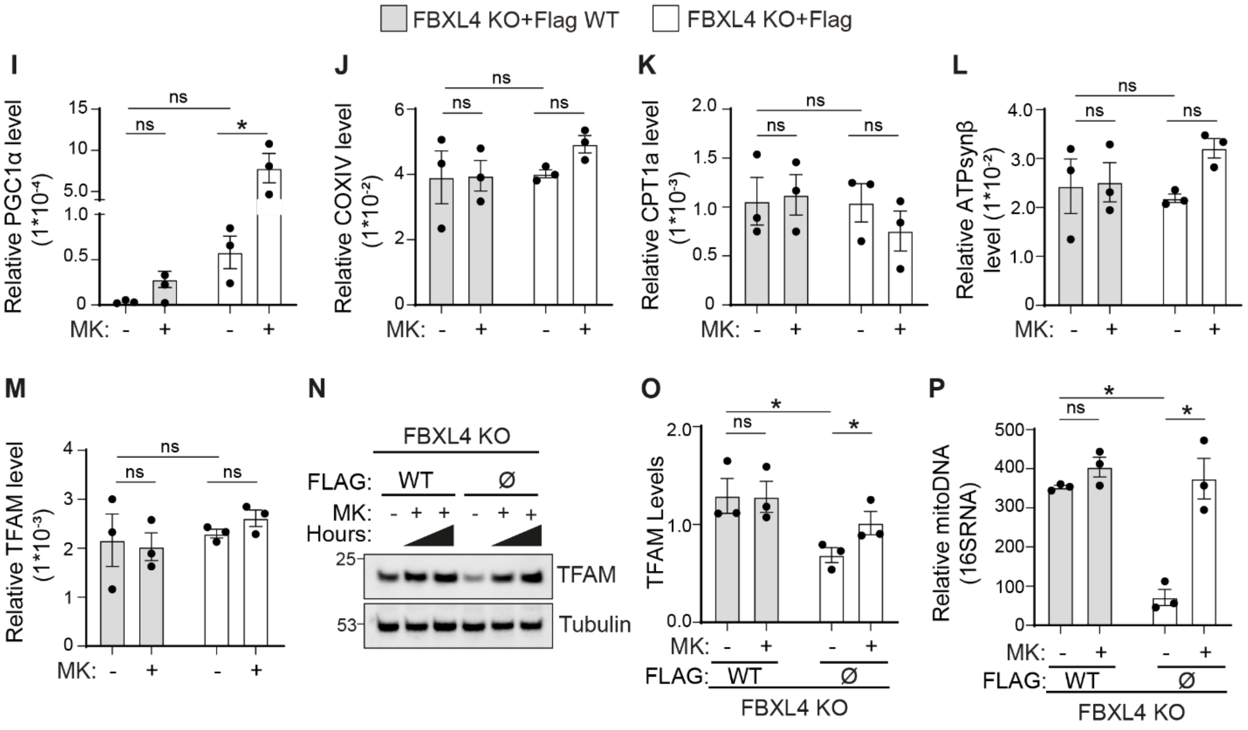

initiated however and will likely contribute to mitochondrial levels after prolonged AMPK activation. This is described in more detail in the additional text starting at the bottom of page 10 in the main manuscript.

3. Some of the representative blots do not seem to match the densitometry shown. For instance, in Figure 3, OMI levels do not seem to be rescued to WT levels by MK-8722 in the representative blot, but are reported to be very similar levels in the densitometry analysis in 3B. Similarly, in EV2A, HSP60 levels are barely detectable in lanes 3+4 of the blot shown, though the densitometry analysis shows approximately equal expression to untreated FBXL4 KO cells. Please comment on this apparent discrepancy and/or select a different blot that is more representative of the quantification shown.

Response: We apologise for the apparent discrepancy – the apparent variation between the blots and quantification was due to variation in loading control between the samples (Fig 3. OMI) and variation in the replicate (Fig.EV2A HSP60 now EV1H). Both set of blots have been changed with a different replicate. We note that all the primary immunoblots from each of the replicates are included in the supplemental data.

4. In multiple figures (in particular, 1A, 1C, EV1A, 2A, EV2A), NIX seems to have an upward shift in MK-8722-treated cells. As NIX has been demonstrated to be phosphorylated (PMID: 28442745, 37833277), the authors could expand upon whether MK-8722 promotes accumulation of post-translationally modified forms of these proteins, and whether evidence exists that this could be direct regulation. This expansion can either be postulated or shown experimentally.

Response: The Reviewer is correct – NIX is phosphorylated in an AMPK dependent manner. However, we would rather not bring attention to this in the current paper as we have another (unrelated) manuscript in preparation that in part examines and reports on this phosphorylation event.

5. Figures would be strengthened by including statistical comparisons across all conditions instead of only selected comparisons that are reported.

Response: We purposely did not include all the seemingly non-relevant comparisons as we felt this made the figures too crowded and harder to interpret. However, we have now gone back and added additional ones. We do note that all the statistical comparisons for each figure are deposited in the supplemental data file.

6. Some statements could be interpreted as overstated and should be reworded. For instance, when describing data in Figures 2A-B (page 7) and 3A (page 9), the authors claim restoration of "mitochondrial proteins" but only demonstrate this for a handful of targets. Addition of "tested" or "select" to describe these data would be more appropriate. On page 7, the authors claim that "loss of FBXL4 catalytic activity is responsible for the observed increase in mitophagy," however, there is little evidence to support the claim that this mutation disrupts catalytic activity. The authors could address this either experimentally or by referencing previously published data. Finally, as noted by the authors, MLN4924 is a broad-spectrum cullin-RING ligase inhibitor that targets more F-box proteins than just FBXL4. The use of terms such as "FBXL4 inhibition" (including in Figure 5) should therefore be revised to "SCF inhibition".

Response: We thank the reviewer for pointing this out and have reworded the statements as suggested throughout the manuscript. For the term on original p7, we have changed "loss of FBXL4 catalytic activity" to "loss of FBXL4 function" and described the mutation as one "that impairs SCF-FBXL4 assembly (El-Hattab et al. 2017; Cao et al. 2023)". The term the term "FBXL4 inhibition" has been changed to "SCF inhibition" in the main text.

In context of restoration of mitochondrial proteins, we have now added three more proteins (MT-CO1, TOM20 in Fig.EV2C-D and TFAM in FigEV2N-O). All these proteins show a similar changes as observed with the previously examined proteins. Furthermore, we see a similar change in mtDNA (Fig.EV2P), which can be correlated with mitochondrial content. The new data is shown below and is part of the new Fig.EV2.

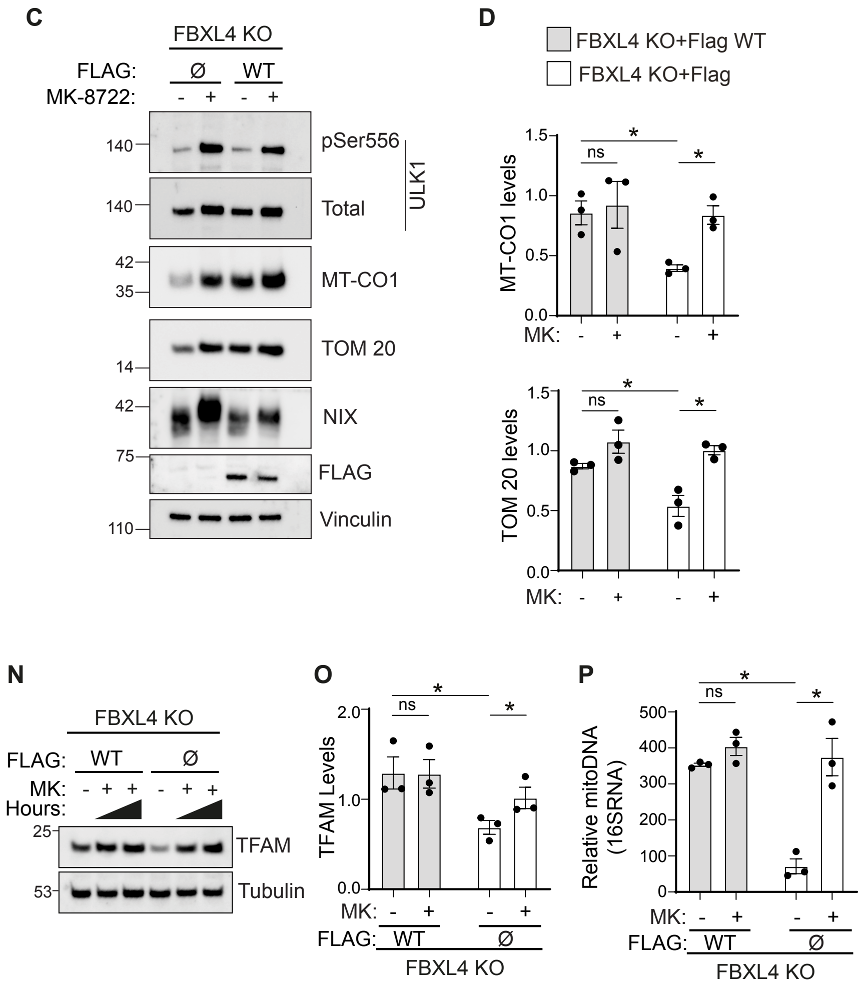

7. Some literature citations could be included such as some on MTDPS13 patient symptom presentation (PMID: 23993193, 23993194, 30804983, 25868664) and recent evidence that FBXL4 works in concert with PPTC7 to facilitate BNIP3/NIX turnover (PMID: 38151018, 38992176, 37833277).

Response: We apologise for the omissions - all these published papers have now been cited in the introduction.

Referee #3 (Remarks for Author):

Rare FBXL4 mutations lead to a mitochondrial depletion syndrome for which there is no rational treatment option. The typical life expectancy is about 3 years old. In 2023 several labs robustly showed that the relevant mechanism involves FBXL4-mediated degradation of the mitophagy adaptors BNIP3 and BNIP3L/NIX. In FBXL4 syndrome patients these factors are elevated and the resultant enhanced mitochondrial turnover leads to depletion of mitochondria. Thus interfering with this mitophagy pathway could restore

mitochondrial mass. Here, the Ganley group build on their published observations that AMPK activators inhibit BNIP3/NIX mitophagy by sequestering ULK1, whilst the PINK/Parkin pathway (which senses damaged mitochondria remains intact). They have now shown that this approach can be used to replenish mitochondria in patient derived cells and in an animal model which recapitulates some aspects of the disease. I think this offers a real possibility of repurposing drugs towards an experimental therapy for children, as any potential negative effects of the drug are offset by the severity of the disease. Thus I am enthusiastic about the manuscript in general and consider the technical quality to be high.

Response: We appreciate the positive and encouraging feedback and acknowledgement the referee's effort, time and constructive comments.

General comments:

I feel that the following points need a bit more discussion. sequestration of ULK1 is likely to result in inhibition of multiple autophagy pathways - the PINK1/Parkin pathway may be an exception.

Response: Indeed, we do believe AMPK activation may block ULK1-dependent autophagy in multiple contexts (which others have also recently published: (Kazyken et al. 2024; Park, Lee, and Kim 2023; Shang et al. 2011). We also have previously demonstrated that AMPK activation blocks iron chelation mediated pexophagy, which is dependent on NIX (Longo et al. 2024; Wilhelm et al. 2022). We therefore tested this pathway in the context of FBXL4 loss. However, we did not observe any significant loss in the peroxisomal proteins catalase or PMP70 in FBXL4 KO compared to WT cells (with or without AMPK activation). This suggests for an alternate E3 ligase (if any) that regulates NIX level on the peroxisome. This data and the discussion have been added in the results section of the manuscript. This is now included in the result section of the manuscript.

The mouse model for chemical depletion uses MLN2924 which will inhibit all Cullin E3 ligases including FBXL4. For most of them this does not matter, but VHL has also been shown to be a regulator of BNIP3 and NIX and a strong suppressor of mitophagy. So the effect on mitochondria in the mouse will likely reflect a combination of these two E3s rather than just FBXL4 as occurs in patients- this needs to be made clearer. Nevertheless the reversal by MK is impressive.

Response: We thank the referee for positive feedback and we do agree that we cannot rule out a contribution of VHL inhibition in the mouse. We have changed the Figure legend titles of the mouse experiments (Fig.4 and 5) from "FBXL4-induced mitophagy" to MLN4924-induced mitophagy. We also now state in the main text on p5 that "Although

MLN4924 is a global SCF E3 ligase inhibitor, and will also block the Von Hippel-Lindau (VHL)-CRL that downregulates HIF1-mediated mitophagy (Ganley and Simonsen 2022), it was shown in cells that MLN4924 mediated increase in NIX was specifically via FBXL4 inhibition (Elcocks et al. 2023)". We also modified the sentence on p13 that introduces the mouse work to: "Given that conditional FBXL4 KO mice are not yet available, we opted for a pharmacological approach to mimic the disease by inhibiting FBXL4 with MLN4924 (though in this instance we cannot rule out that inhibition of other CRLs, such as VHL, may contribute to any observed phenotypes)".

Figure 3, shows a couple of patient derived fibroblast lines with enhanced mitophagy compared with a single wild type line. What is never quite clear is what is the variation between wild type individuals.

Response: Unfortunately we only have one fibroblast line from a heathy individual, from the original study with the patient lines (Bonnen et al. 2013). However, the low level of basal mitophagy is consistent with our WT ARPE19 cells, as well as our previous work using different primary human fibroblasts and the same mitophagy assay (Allen et al. 2013). We therefore feel the results here are representative in general and not an exception.

At some points there is an emphasis on NIX rather than BNIP3- this needs some explanation.

Response: We apologise for this confusion – it was a simple mistake on our part and we did not intend to imply that the pathway was NIX focussed. We have now included BNIP3 blots for all figures and include BNIP3 in the results and discussion. As expected, the change in BNIP3 levels in FBXL4 KO mirrors that of NIX.

To more definitively analyse the contribution of NIX and BNIP3 to mitophagy in our ARPE19 cells, we created NIX and BNIP3 single and DKO cells. Upon induction of mitophagy with MLN4924, we found that loss of NIX had a greater impact on mitophagy compared to BNIP3, with almost complete mitophagy loss in the DKO cells. Therefore NIX is the major regulator of mitophagy in ARPE19 cells, being only partially redundant with BNIP3. These new data are shown below and in new Fig.EV1A.

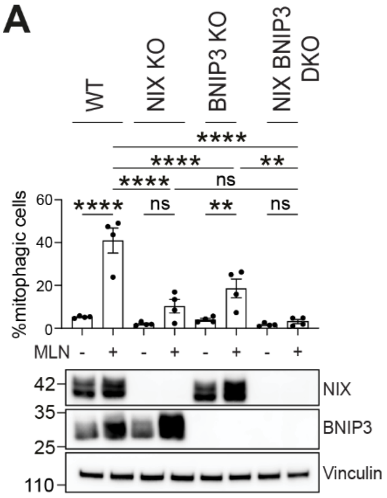

References:

Allen, G. F., R. Toth, J. James, and I. G. Ganley. 2013. 'Loss of iron triggers PINK1/Parkin-independent mitophagy', *EMBO Rep*, 14: 1127-35.

Bonnen, P. E., J. W. Yarham, A. Besse, P. Wu, E. A. Faqeih, A. M. Al-Asmari, M. A. Saleh, W. Eyaid, A. Hadeel, L. He, F. Smith, S. Yau, E. M. Simcox, S. Miwa, T. Donti, K. K. Abu-Amero, L. J. Wong, W. J. Craigen, B. H. Graham, K. L. Scott, R. McFarland, and R. W. Taylor. 2013. 'Mutations in FBXL4 cause mitochondrial encephalopathy and a disorder of mitochondrial DNA maintenance', *Am J Hum Genet*, 93: 471-81.

Cao, Y., J. Zheng, H. Wan, Y. Sun, S. Fu, S. Liu, B. He, G. Cai, Y. Cao, H. Huang, Q. Li, Y. Ma, S. Chen, F. Wang, and H. Jiang. 2023. 'A mitochondrial SCF-FBXL4 ubiquitin E3 ligase complex degrades BNIP3 and NIX to restrain mitophagy and prevent mitochondrial disease', *EMBO J*, 42: e113033.

El-Hattab, A. W., H. Dai, M. Almannai, J. Wang, E. A. Faqeih, A. Al Asmari, M. A. M. Saleh, M. A. O. Elamin, M. Alfadhel, F. S. Alkuraya, M. Hashem, M. S. Aldosary, R. Almass, F. B. Almutairi, M. Alsagob, M. Al-Owain, S. Al-Sharfa, Z. N. Al-Hassnan, Z. Rahbeeni, M. A. Al-Muhaizea, N. Makhseed, G. K. Foskett, D. A. Stevenson, N. Gomez-Ospina, C. Lee, R. G. Boles, S. A. Schrier Vergano, S. B. Wortmann, W. Sperl, T. Opladen, G. F. Hoffmann, M. Hempel, H. Prokisch, B. Alhaddad, J. A. Mayr, W. Chan, N. Kaya, and L. C. Wong. 2017. 'Molecular and clinical spectra of FBXL4 deficiency', *Hum Mutat*, 38: 1649-59.

Elcocks, H., A. J. Brazel, K. R. McCarron, M. Kaulich, K. Husnjak, H. Mortiboys, M. J. Clague, and S. Urbe. 2023. 'FBXL4 ubiquitin ligase deficiency promotes mitophagy by elevating NIX levels', *EMBO J*, 42: e112799.

Fraguas Bringas, C., M. S. Ahangar, J. Cuenco, H. Liu, A. B. Addinsall, M. Lindahl, A. J. Ovens, M. A. Febbraio, M. Foretz, O. Goransson, J. W. Scott, E. Zeqiraj, and K. Sakamoto. 2025. 'Mechanism and cellular actions of the potent AMPK inhibitor BAY-3827', *Sci Adv*, 11: eadx2434.

Ganley, I. G., and A. Simonsen. 2022. 'Diversity of mitophagy pathways at a glance', *J Cell Sci*, 135.

Graham, G. G., J. Punt, M. Arora, R. O. Day, M. P. Doogue, J. K. Duong, T. J. Furlong, J. R. Greenfield, L. C. Greenup, C. M. Kirkpatrick, J. E. Ray, P. Timmins, and K. M. Williams. 2011. 'Clinical pharmacokinetics of metformin', *Clin Pharmacokinet*, 50: 81-98.

Handschin, C., and B. M. Spiegelman. 2006. 'Peroxisome proliferator-activated receptor gamma coactivator 1 coactivators, energy homeostasis, and metabolism', *Endocr Rev*, 27: 728-35.

Kazyken, Dubek, Sydney G. Dame, Claudia Wang, Maxwell Wadley, and Diane C. Fingar. 2024. 'Unexpected roles for AMPK in the suppression of autophagy and the reactivation of MTORC1 signaling during prolonged amino acid deprivation', *Autophagy*, 20: 2017-40.

Longo, M., A. Bishnu, P. Risiglione, L. Montava-Garriga, J. Cuenco, K. Sakamoto, C. MacKintosh, and I. G. Ganley. 2024. 'Opposing roles for AMPK in regulating distinct mitophagy pathways', *Mol Cell*, 84: 4350-67 e9.

Malik, N., B. I. Ferreira, P. E. Hollstein, S. D. Curtis, E. Trefts, S. Weiser Novak, J. Yu, R. Gilson, K. Hellberg, L. Fang, A. Sheridan, N. Hah, G. S. Shadel, U. Manor, and R. J. Shaw. 2023. 'Induction of lysosomal and mitochondrial biogenesis by AMPK phosphorylation of FNIP1', *Science*, 380: eabj5559.

Myers, R. W., H. P. Guan, J. Ehrhart, A. Petrov, S. Prahalada, E. Tozzo, X. Yang, M. M. Kurtz, M. Trujillo, D. Gonzalez Trotter, D. Feng, S. Xu, G. Eiermann, M. A. Holahan, D. Rubins, S. Conarello, X. Niu, S. C. Souza, C. Miller, J. Liu, K. Lu, W. Feng, Y. Li, R. E. Painter, J. A. Milligan, H. He, F. Liu, A. Ogawa, D. Wisniewski, R. J. Rohm, L. Wang, M. Bunzel, Y. Qian, W. Zhu, H. Wang, B. Bennet, L. LaFranco Scheuch, G. E. Fernandez, C. Li, M. Klimas, G. Zhou, M. van Heek, T. Biftu, A. Weber, D. E. Kelley, N. Thornberry, M. D. Erion, D. M. Kemp, and I. K. Sebhat. 2017. 'Systemic pan-AMPK activator MK-8722 improves glucose homeostasis but induces cardiac hypertrophy', *Science*, 357: 507-11.

Park, J. M., D. H. Lee, and D. H. Kim. 2023. 'Redefining the role of AMPK in autophagy and the energy stress response', *Nat Commun*, 14: 2994.

Shang, L., S. Chen, F. Du, S. Li, L. Zhao, and X. Wang. 2011. 'Nutrient starvation elicits an acute autophagic response mediated by Ulk1 dephosphorylation and its subsequent dissociation from AMPK', *Proc Natl Acad Sci U S A*, 108: 4788-93.

Sundelin, E., J. B. Jensen, S. Jakobsen, L. C. Gormsen, and N. Jessen. 2020. 'Metformin Biodistribution: A Key to Mechanisms of Action?', *J Clin Endocrinol Metab*, 105.

Wilhelm, L. P., J. Zapata-Munoz, B. Villarejo-Zori, S. Pellegrin, C. M. Freire, A. M. Toye, P. Boya, and I. G. Ganley. 2022. 'BNIP3L/NIX regulates both mitophagy and pexophagy', *EMBO J*, 41: e111115.

19th May 2026

Dear Prof. Ganley,

Thank you for submitting your revised manuscript to EMBO Molecular Medicine. Your manuscript will be ready for acceptance pending the following final amendments:

1) In the main manuscript file, please do the following:
- Please address all comments suggested by our data editors listed below:
o Figure legends:
1. Please note that the exact p values are not provided in the legends of 1B,D,F; 2B,D-H; 3B,D; 4B,D,F; 5C,E; EV-1A,C,E,G,I,K; EV-2D,O,P; EV-4A,B,D; EV-5B.
2. Please indicate the statistical test used for data analysis in the legends of figures EV-2G,H.
3. Please note that information related to n is missing in the legends of EV-2G,H.
4. Please note that the error bars are not defined in the legends of figures EV-2G,H.
- Please update the order and the headings of the sections in the manuscript text to: Abstract / The Paper Explained / Introduction / Results / Discussion / Methods / Data Availability / Acknowledgements / Disclosure and Competing Interests Statement / References / Figure Legends Expanded View Figure Legends
- Please include structured Methods section that includes a Reagents and Tools Table (should be uploaded as a separate file) followed by a Methods and Protocols section. More information on how to adhere to this format as well as downloadable templates (.docx) for the Reagents and Tools Table can be found in our author guidelines:
https://www.embopress.org/page/journal/17574684/authorguide#structuredmethods
An example of a paper with Structured Methods can be found here:
https://www.embopress.org/doi/full/10.1038/s44320-024-00037-6#sec-4
- Indicate in legends number and nature of replicates and exact p= values, not a range, along with the statistical test used. To keep the figures "clear" some authors found providing an Appendix table Sx with all exact p-values preferable. You are welcome to do this if you want to.
- Please add the heading "Disclosure and competing interests statement" to the conflict of interests statement.
- Author contributions: Please remove it from the manuscript and specify author contributions in our submission system. CRediT has replaced the traditional author contributions section because it offers a systematic machine-readable author contributions format that allows for more effective research assessment. You are encouraged to use the free text boxes beneath each contributing author's name to add specific details on the author's contribution. More information is available in our guide to authors:
https://www.embopress.org/page/journal/17574684/authorguide#authorshipguidelines
- Correct the reference citation in the reference list. Where there are more than 10 authors on a paper, 10 will be listed, followed by "et al.". Please check "Author Guidelines" for more information.
https://www.embopress.org/page/journal/17574684/authorguide#referencesformat
2) Funding: Please rename it to "Acknowledgements" and ensure that all funding institutions mentioned in the manuscript text are included in the list of funders in our system. The list will be linked from PubMed, so it is important that it is complete and accurate. Please do not use the comments box underneath the list, as this cannot be linked.
3) The Paper Explained: Please provide "The Paper Explained" and add it to the main manuscript text. Please check "Author Guidelines" for more information. https://www.embopress.org/page/journal/17574684/authorguide#researcharticleguide
4) Synopsis: Every published paper now includes a 'Synopsis' to further enhance discoverability. Synopses are displayed on the journal webpage and are freely accessible to all readers. They include separate synopsis image and synopsis text.
- Synopsis image: Please provide a visual abstract as a high-resolution jpeg file, exactly 550 px-wide x 300 pixels high to illustrate your article.
- Synopsis text: Please provide a short standfirst (maximum of 300 characters, including space) as well as 2-5 one sentence bullet points that summarise the paper as a .doc file. Please write the bullet points to summarise the key NEW findings. They should be designed to be complementary to the abstract - i.e. not repeat the same text. We encourage inclusion of key acronyms and quantitative information (maximum of 30 words / bullet point). Please use the passive voice.
- Please check your synopsis text and image before submission with your revised manuscript. Please be aware that in the proof stage minor corrections only are allowed (e.g., typos).
5) As part of the EMBO Publications transparent editorial process (see our Editorial at http://embomolmed.embopress.org/content/2/9/329), EMBO Molecular Medicine will publish online a Review Process File (RPF) to accompany accepted manuscripts. This file will be published in conjunction with your paper and will include the anonymous referee reports, your point-by-point response and all pertinent correspondence relating to the manuscript. Let us know if you want to remove or not any figures from it prior to publication. Please note that the Authors checklist will be published at the end of the RPF.
6) Please provide a point-by-point letter INCLUDING my comments as well as the reviewer's reports and your detailed responses (as Word file).

I look forward to reading a new revised version of your manuscript as soon as possible.

Yours sincerely,

Zeljko Durdevic

Zeljko Durdevic
Senior Editor
EMBO Molecular Medicine

*** Instructions to submit your revised manuscript ***

*** PLEASE NOTE *** As part of the EMBO Publications transparent editorial process initiative (see our Editorial at https://www.embopress.org/doi/pdf/10.1002/emmm.201000094), EMBO Molecular Medicine will publish online a Review Process File to accompany accepted manuscripts.

In the event of acceptance, this file will be published in conjunction with your paper and will include the anonymous referee reports, your point-by-point response and all pertinent correspondence relating to the manuscript. If you do NOT want this file to be published, please inform the editorial office at contact@embomolmed.org.

*****

When preparing your revised manuscript, please refer to our guidelines: https://link.springer.com/journal/44321/submission-guidelines#cms-Revised-submissions. We perform an initial quality control of all revised manuscripts before re-review; failure to include requested items will delay the evaluation of your revision.

We require:

1) A .docx formatted version of the manuscript text (including legends for main figures, EV figures and tables). Please make sure that the changes are highlighted to be clearly visible.

2) Individual production quality figure files as .eps, .tif, .jpg (one file per figure). For guidance, download the 'Figure Guide PDF': https://media.springernature.com/original/springer-cms/rest/v1/content/27825798/data/v1.

3) A .docx formatted letter INCLUDING the reviewers' reports and your detailed point-by-point responses to their comments. As part of the EMBO Press transparent editorial process, the point-by-point response is part of the Review Process File (RPF), which will be published alongside your paper.

4) A complete author checklist, which you can download from our author guidelines. Please insert information in the checklist that is also reflected in the manuscript. The completed author checklist will also be part of the RPF.

5) Please note that all corresponding authors are required to supply an ORCID ID for their name upon submission of a revised manuscript.

6) It is mandatory to include a 'Data Availability' section after the Materials and Methods. Before submitting your revision, primary datasets produced in this study need to be deposited in an appropriate public database, and the accession numbers and database listed under 'Data Availability'. Please remember to provide a reviewer password if the datasets are not yet public.

In case you have no data that requires deposition in a public database, please state so in this section. Note that the Data Availability Section is restricted to new primary data that are part of this study.

7) For data quantification: please specify the name of the statistical test used to generate error bars and P values, the number (n) of independent experiments (specify technical or biological replicates) underlying each data point and the test used to calculate p-values in each figure legend. The figure legends should contain a basic description of n, P and the test applied. Graphs must include a description of the bars and the error bars (s.d., s.e.m.).

8) At EMBO Press we ask authors to provide source data for the main manuscript figures. You will receive a separate email with instructions for providing source data with your revised manuscript, including how to upload and organize the files.

9) Our journal encourages inclusion of *data citations in the reference list* to directly cite datasets that were re-used and obtained from public databases. Data citations in the article text are distinct from normal bibliographical citations and should directly link to the database records from which the data can be accessed. In the main text, data citations are formatted as follows: "Data ref: Smith et al, 2001" or "Data ref: NCBI Sequence Read Archive PRJNA342805, 2017". In the Reference list, data citations must be labeled with "[DATASET]". A data reference must provide the database name, accession number/identifiers and a resolvable link to the landing page from which the data can be accessed at the end of the reference.

10) We replaced Supplementary Information with Expanded View (EV) Figures and Tables that are collapsible/expandable online. A maximum of 5 EV Figures can be typeset. EV Figures should be cited as 'Figure EV1, Figure EV2" etc... in the text and their respective legends should be included in the main text after the legends of regular figures.

- For the figures that you do NOT wish to display as Expanded View figures, they should be bundled together with their legends in a single PDF file called *Appendix*, which should start with a short Table of Content. Appendix figures should be referred to in the main text as: "Appendix Figure S1, Appendix Figure S2" etc.

- Additional Tables/Datasets should be labeled and referred to as Table EV1, Dataset EV1, etc. Legends have to be provided in a separate tab in case of .xls files. Alternatively, the legend can be supplied as a separate text file (README) and zipped together with the Table/Dataset file.

11) The paper explained: EMBO Molecular Medicine articles are accompanied by a summary of the articles to emphasize the major findings in the paper and their medical implications for the non-specialist reader. Please provide a draft summary of your article highlighting

- the medical issue you are addressing,

- the results obtained and

- their clinical impact.

This may be edited to ensure that readers understand the significance and context of the research. Please refer to any of our published articles for an example.

12) Author contributions: You will be asked to provide CRediT (Contributor Role Taxonomy) terms in the submission system. These replace a narrative author contribution section in the manuscript.

13) A Conflict of Interest statement should be provided in the main text.

14) Every published paper includes a 'Synopsis' to further enhance discoverability. Synopses are displayed on the journal webpage and are freely accessible to all readers. They include a short stand first (maximum of 300 characters, including space) as well as 2-5 one-sentences bullet points that summarizes the paper. Please write the bullet points to summarize the key NEW findings. They should be designed to be complementary to the abstract - i.e. not repeat the same text. We encourage inclusion of key acronyms and quantitative information (maximum of 30 words / bullet point). Please use the passive voice. Please attach these in a separate file or send them by email, we will incorporate them accordingly.

Please also suggest a striking image or visual abstract to illustrate your article as a PNG file 550 px wide x 300 px high.

15) Include a Reagents and Tools Table as part of the Methods section, which can be downloaded from our author guidelines.

EMBO Molecular Medicine has a "scooping protection" policy, whereby similar findings that are published by others during review or revision are not a criterion for rejection. Should you decide to submit a revised version, I do ask that you get in touch after three months if you have not completed it, to update us on the status.

*Additional important information regarding Figures

Each figure should be given in a separate file and should have the following resolution:
Graphs 800-1,200 DPI
Photos 400-800 DPI
Colour (only CMYK) 300-400 DPI"

Figures are not edited by the production team. All lettering should be the same size and style; figure panels should be indicated by capital letters (A, B, C etc). Gridlines are not allowed except for log plots. Figures should be numbered in the order of their appearance in the text with Arabic numerals. Each Figure must have a separate legend and a caption is needed for each panel.

*Additional important information regarding figures and illustrations can be found at https://media.springernature.com/original/springer-cms/rest/v1/content/27825798/data/v1

***** Reviewer's comments *****

Referee #1 (Comments on Novelty/Model System for Author):

I do not have further comments, I think the manuscript is of high technical quality, and supports the conclusions of the authors

Referee #1 (Remarks for Author):

The authors addressed all concerns.

Referee #2 (Comments on Novelty/Model System for Author):

This is a concise and very interesting study that identifies AMPK activation as a potential therapeutic strategy for Mitochondrial DNA Depletion Syndrome 13 (MTDPS13), a rare, inborn error of metabolism caused by mutations in the FBXL4 gene in humans. Building on previous work that demonstrated that AMPK activation can inhibit receptor-mediated mitophagy through the sequestration of ULK1, the authors demonstrate that pharmacological activation of AMPK through multiple independent compounds can rescue the elevated mitophagy in FBXL4-deficient cells and patient fibroblasts, as well as in a chemically-induced in vivo model of FBXL4-deficiency. The study has now addressed all of my previous concerns, and in my opinion should be accepted for publication.

Referee #2 (Remarks for Author):

The authors have addressed all of my major concerns. Congratulations to the authors on a great study!

The authors addressed the remaining editorial issues.

3rd Jun 2026

Dear Prof. Ganley,

We are pleased to inform you that your manuscript is accepted for publication and is now being sent to our publisher to be included in the next available issue of EMBO Molecular Medicine.

Your manuscript will be processed for publication by EMBO Press. It will be copy edited and you will receive page proofs prior to publication. Please note that you will be contacted by Springer Nature Author Services to complete licensing and payment information.

You may qualify for financial assistance for your publication charges - either via a Springer Nature fully open access agreement or an EMBO initiative. Check your eligibility: https://link.springer.com/journal/44321/how-to-publish-with-us

Should you be planning a Press Release on your article, please notify the production team via this form in order to coordinate publication and release dates: https://forms.cloud.microsoft/e/eH4A8JVP6g

If you have any questions, please do not hesitate to contact the Editorial Office. Thank you for your contribution to EMBO Molecular Medicine.

Yours sincerely,
Zeljko Durdevic

Zeljko Durdevic
Senior Editor
EMBO Molecular Medicine

>>> Please note that it is EMBO Molecular Medicine policy for the transcript of the editorial process (containing referee reports and your response letter) to be published as an online supplement to each paper. If you do NOT want this, you will need to inform the Editorial Office via email immediately. More information is available here: https://link.springer.com/partners/embo-press/editorial-policies#Peer%20review

EMBO Press Author Checklist

Corresponding Author Name: Ian Ganley	
Journal Submitted to: EMBO Molecular Medicine	
Manuscript Number: EMM-2025-23166-V2	

USEFUL LINKS FOR COMPLETING THIS FORM
The EMBO Journal - Author Guidelines
EMBO Reports - Author Guidelines
Molecular Systems Biology - Author Guidelines
EMBO Molecular Medicine - Author Guidelines

Reporting Checklist for Life Science Articles (updated January
This checklist is adapted from Materials Design Analysis Reporting (MDAR) Checklist for Authors. MDAR establishes a minimum set of requirements in transparent reporting in the life sciences (see Statement of Task: 10.31222/osf.io/9sm4x). Please follow the journal's guidelines in preparing your
Please note that a copy of this checklist will be published alongside your article.

Abridged guidelines for figures
1. Data
The data shown in figures should satisfy the following conditions:
- the data were obtained and processed according to the field's best practice and are presented to reflect the results of the experiments in an accurate and unbiased manner.
- ideally, figure panels should include only measurements that are directly comparable to each other and obtained with the same assay.
- plots include clearly labeled error bars for independent experiments and sample sizes. Unless justified, error bars should not be shown for technical
- if n<5, the individual data points from each experiment should be plotted. Any statistical test employed should be justified.
- Source Data should be included to report the data underlying figures according to the guidelines set out in the authorship guidelines on Data

2. Captions
Each figure caption should contain the following information, for each panel where they are relevant:
- a specification of the experimental system investigated (eg cell line, species name).
- the assay(s) and method(s) used to carry out the reported observations and measurements.
- an explicit mention of the biological and chemical entity(ies) that are being measured.
- an explicit mention of the biological and chemical entity(ies) that are altered/varied/perturbed in a controlled manner.
- the exact sample size (n) for each experimental group/condition, given as a number, not a range;
- a description of the sample collection allowing the reader to understand whether the samples represent technical or biological replicates (including how many animals, litters, cultures, etc.).
- a statement of how many times the experiment shown was independently replicated in the laboratory.
- definitions of statistical methods and measures:
  - common tests, such as t-test (please specify whether paired vs. unpaired), simple χ2 tests, Wilcoxon and Mann-Whitney tests, can be unambiguously identified by name only, but more complex techniques should be described in the methods section;
  - are tests one-sided or two-sided?
  - are there adjustments for multiple comparisons?
  - exact statistical test results, e.g., P values = x but not P values < x;
  - definition of 'center values' as median or average;
  - definition of error bars as s.d. or s.e.m.

Please complete ALL of the questions below.
Select "Not Applicable" only when the requested information is not relevant for your study.

Materials

Newly Created Materials	Information included in the manuscript?	In which section is the information available? (Reagents and Tools Table, Materials and Methods, Figures, Data Availability Section)
New materials and reagents need to be available; do any restrictions apply?	Not Applicable	Materials&Methods

Antibodies	Information included in the manuscript?	In which section is the information available? (Reagents and Tools Table, Materials and Methods, Figures, Data Availability Section)
For **antibodies** provide the following information:<br>- Commercial antibodies: RRID (if possible) or supplier name, catalogue number and or/clone number<br>- Non-commercial: RRID or citation	Yes	Materials&Methods

DNA and RNA sequences	Information included in the manuscript?	In which section is the information available? (Reagents and Tools Table, Materials and Methods, Figures, Data Availability Section)
**Short novel DNA or RNA including primers, probes**: provide the sequences.	Yes	Materials&Methods

Cell materials	Information included in the manuscript?	In which section is the information available? (Reagents and Tools Table, Materials and Methods, Figures, Data Availability Section)
**Cell lines:** Provide species information, strain. Provide accession number in repository **OR** supplier name, catalog number, clone number, and/**OR** RRID.	Yes	Materials&Methods
**Primary cultures:** Provide species, strain, sex of origin, genetic modification status.	Yes	Materials&Methods
Report if the cell lines were recently **authenticated** (e.g., by STR profiling) and tested for mycoplasma contamination.	Yes	Materials&Methods

Experimental animals	Information included in the manuscript?	In which section is the information available? (Reagents and Tools Table, Materials and Methods, Figures, Data Availability Section)
**Laboratory animals or Model organisms:** Provide species, strain, sex, age, genetic modification status. Provide accession number in repository **OR** supplier name, catalog number, clone number, **OR** RRID.	Yes	Materials&Methods
**Animal observed in or captured from the field:** Provide species, sex, and age where possible.	Yes	Materials&Methods
Please detail **housing and husbandry conditions**.	Yes	Materials&Methods

Plants and microbes	Information included in the manuscript?	In which section is the information available? (Reagents and Tools Table, Materials and Methods, Figures, Data Availability Section)
**Plants:** provide species and strain, ecotype and cultivar where relevant, unique accession number if available, and source (including location for collected wild specimens).	Not Applicable	
**Microbes:** provide species and strain, unique accession number if available, and source.	Not Applicable	

Human research participants	Information included in the manuscript?	In which section is the information available? (Reagents and Tools Table, Materials and Methods, Figures, Data Availability Section)
If collected and within the bounds of privacy constraints report on age, sex and gender or ethnicity for all study participants.	Not Applicable	

Core facilities	Information included in the manuscript?	In which section is the information available? (Reagents and Tools Table, Materials and Methods, Figures, Data Availability Section)
If your work benefited from core facilities, was their service mentioned in the acknowledgments section?	Not Applicable	

Design

Study protocol	Information included in the manuscript?	In which section is the information available? (Reagents and Tools Table, Materials and Methods, Figures, Data Availability Section)
If study protocol has been **pre-registered, provide DOI in the manuscript**. For clinical trials, provide the trial registration number **OR** cite DOI.	Not Applicable	
Report the **clinical trial registration number** (at ClinicalTrials.gov or equivalent), where applicable.	Not Applicable	

Laboratory protocol	Information included in the manuscript?	In which section is the information available? (Reagents and Tools Table, Materials and Methods, Figures, Data Availability Section)
Provide DOI OR other citation details if **external detailed step-by-step protocols** are available.	Not Applicable	

Experimental study design and statistics	Information included in the manuscript?	In which section is the information available? (Reagents and Tools Table, Materials and Methods, Figures, Data Availability Section)
Include a statement about **sample size** estimate even if no statistical methods were used.	Yes	Materials&Methods
Were any steps taken to minimize the effects of subjective bias when allocating animals/samples to treatment (e.g. **randomization procedure**)? If yes, have they been described?	Yes	Materials&Methods
Include a statement about **blinding** even if no blinding was done.	Yes	Materials&Methods
Describe **inclusion/exclusion criteria** if samples or animals were excluded from the analysis. Were the criteria pre-established?<br><br>If sample or data points were omitted from analysis, report if this was due to attrition or intentional exclusion and provide justification.	Not Applicable	
For every figure, are **statistical tests** justified as appropriate? Do the data meet the assumptions of the tests (e.g., normal distribution)? Describe any methods used to assess it. Is there an estimate of variation within each group of data? Is the variance similar between the groups that are being statistically compared?	Yes	Figure Legends and Materials&Methods

Sample definition and in-laboratory replication	Information included in the manuscript?	In which section is the information available? (Reagents and Tools Table, Materials and Methods, Figures, Data Availability Section)
In the figure legends: state number of times the experiment was **replicated** in laboratory.	Yes	Figure Legends
In the figure legends: define whether data describe **technical or biological replicates**.	Yes	Figure Legends

Ethics

Ethics	Information included in the manuscript?	In which section is the information available? (Reagents and Tools Table, Materials and Methods, Figures, Data Availability Section)
Studies involving **human participants**: State details of **authority granting ethics approval** (IRB or equivalent committee(s), provide reference number for approval.	Not Applicable	
Studies involving **human participants**: Include a statement confirming that **informed consent** was obtained from all subjects and that the experiments conformed to the principles set out in the WMA Declaration of Helsinki and the Department of Health and Human Services Belmont Report.	Not Applicable	
Studies involving **human participants: For publication of patient photos**, include a statement confirming that consent to publish was obtained.	Not Applicable	
Studies involving experimental **animals**: State details of **authority granting ethics approval** (IRB or equivalent committee(s), provide reference number for approval. Include a statement of compliance with ethical regulations.	Yes	Materials&Methods
Studies involving **specimen and field samples:** State if relevant **permits** obtained, provide details of authority approving study; if none were required, explain why.	Not Applicable	

Dual Use Research of Concern (DURC)	Information included in the manuscript?	In which section is the information available? (Reagents and Tools Table, Materials and Methods, Figures, Data Availability Section)
Could your study fall under dual use research restrictions? Please check biosecurity documents and list of **select agents and toxins** (CDC): https://www.selectagents.gov/sat/list.htm	Not Applicable	
If you used a select agent, is the security level of the lab appropriate and reported in the manuscript?	Not Applicable	
If a study is subject to dual use research of concern regulations, is the name of the **authority granting approval and reference number** for the regulatory approval provided in the manuscript?	Not Applicable	

Reporting

The MDAR framework recommends adoption of discipline-specific guidelines, established and endorsed through community initiatives. Journals have their own policy about requiring specific guidelines and recommendations to complement MDAR.

Adherence to community standards	Information included in the manuscript?	In which section is the information available? (Reagents and Tools Table, Materials and Methods, Figures, Data Availability Section)
State if relevant guidelines or checklists (e.g., **ICMJE, MIBBI, ARRIVE, PRISMA**) have been followed or provided.	Not Applicable	
For **tumor marker prognostic studies,** we recommend that you follow the **REMARK** reporting guidelines (see link list at top right). See author guidelines, under 'Reporting Guidelines'. Please confirm you have followed these guidelines.	Not Applicable	
For **phase II and III randomized controlled trials**, please refer to the **CONSORT** flow diagram (see link list at top right) and submit the CONSORT checklist (see link list at top right) with your submission. See author guidelines, under 'Reporting Guidelines'. Please confirm you have submitted this list.	Not Applicable	

Data Availability

Data availability	Information included in the manuscript?	In which section is the information available? (Reagents and Tools Table, Materials and Methods, Figures, Data Availability Section)
Have **primary datasets** been deposited according to the journal's guidelines (see 'Data Deposition' section) and the respective accession numbers provided in the Data Availability Section?	Not Applicable	
Were **human clinical and genomic datasets** deposited in a public access-controlled repository in accordance to ethical obligations to the patients and to the applicable consent agreement?	Not Applicable	
Are **computational models** that are central and integral to a study available without restrictions in a machine-readable form? Were the relevant accession numbers or links provided?	Not Applicable	
If publicly available data were reused, provide the respective **data citations in the reference list**.	Not Applicable	
