# Supplementary material for: Activation of AMPK as a therapeutic strategy for FBXL4-related mitochondrial DNA depletion syndrome
Source: EMBO Mol Med. 2026 Jun 17;18(8):3109–36. doi: 10.1038/s44321-026-00471-z (PMC13470155; doi:10.1038/s44321-026-00471-z)
Supplement: Supplementary file 2 [file 44321_2026_471_MOESM8_ESM.pdf]

Expanded View Figures

Figure EV1. AMPK activators inhibit FBXL4-induced mitophagy.

(**A**) Flow cytometry analysis of WT, NIX knockout (NIX KO), BNIP3 knockout (BNIP3 KO) and NIX and BNIP3 double knockout (NIX BNIP3 DKO) treated with 5 µM of MLN4924 for 24 h. The percentage of ARPE-19 cells having a high mCherry/GFP ratio was quantified for each condition. Data represents mean ± SEM ($n = 4$). Significance calculated using ordinary two-way ANOVA and Sidak's multiple comparisons test. Representative blot of NIX and BNIP3 in knockout cells is shown below. (**B**) Representative immunoblot of indicated protein in FBXL4 knockout (FBXL4 KO) and WT rescue cells treated with/without MK-8722 (MK, 10 µM). (**C**) Quantification of the immunoblot data in panel (**B**) of protein levels normalized to loading controls. Data represents mean ± SEM ($n = 3$), significance calculated using two-way ANOVA and Uncorrected Fisher's LSD. (**D**) Representative immunoblot of indicated proteins from ARPE-19 wildtype (WT) and FBXL4 knockout (FBXL4 KO) treated with/without MK-8722 (MK, 10 µM), PXI-770 (PXL, 50 µM), and BI-9774 (BI, 10 µM) for 48 h. (**E**) Quantification of the immunoblot data in panel D with protein levels normalized to loading controls. Data represents mean ± SEM ($n = 3$), significance calculated using ordinary one-way ANOVA and Tukey's multiple comparisons test. (**F**) Representative wide-field images of FBXL4 KO ARPE-19 *mito*-QC cells, treated as in D. Red puncta represent mitolysosomes while mitochondrial network appears yellow. Scale bar: 20 µm. (**G**) Mitolysosomes quantitation per cell. Data represents mean ± SEM ($n = 3$), significance calculated using ordinary one-way ANOVA and Tukey's multiple comparison test. (**H**) Representative immunoblots of indicated proteins from FBXL4 knockout (FBXL4 KO) cells treated with MK-8722 (MK, 10 µM) and BAY-3827 (5 µM) for 48 h. (**I**) Quantification of the immunoblot data in panel (**H**) with protein levels normalized to loading controls. Data represents mean ± SEM ($n = 3$), significance calculated using ordinary one-way ANOVA and Tukey's multiple comparisons test. (**J**) Representative wide-field images of WT and FBXL4 KO ARPE-19 mito-QC cells, treated with MK-8722 (MK, 10 µM) and BAY-3827 (5 µM) for 48 h. Red puncta represent mitolysosomes while mitochondrial network appears yellow. Scale bar: 20 µm. (**K**) Quantification of the number of mitolysosomes per cell. Data represents mean ± SEM ($n = 3$), significance calculated using ordinary one-way ANOVA and Tukey's multiple comparison test. For all the experiments, statistical significance is depicted as *$p < 0.05$, **$p < 0.01$, ***$p < 0.001$, and ****$p < 0.0001$.

 © The Author(s)

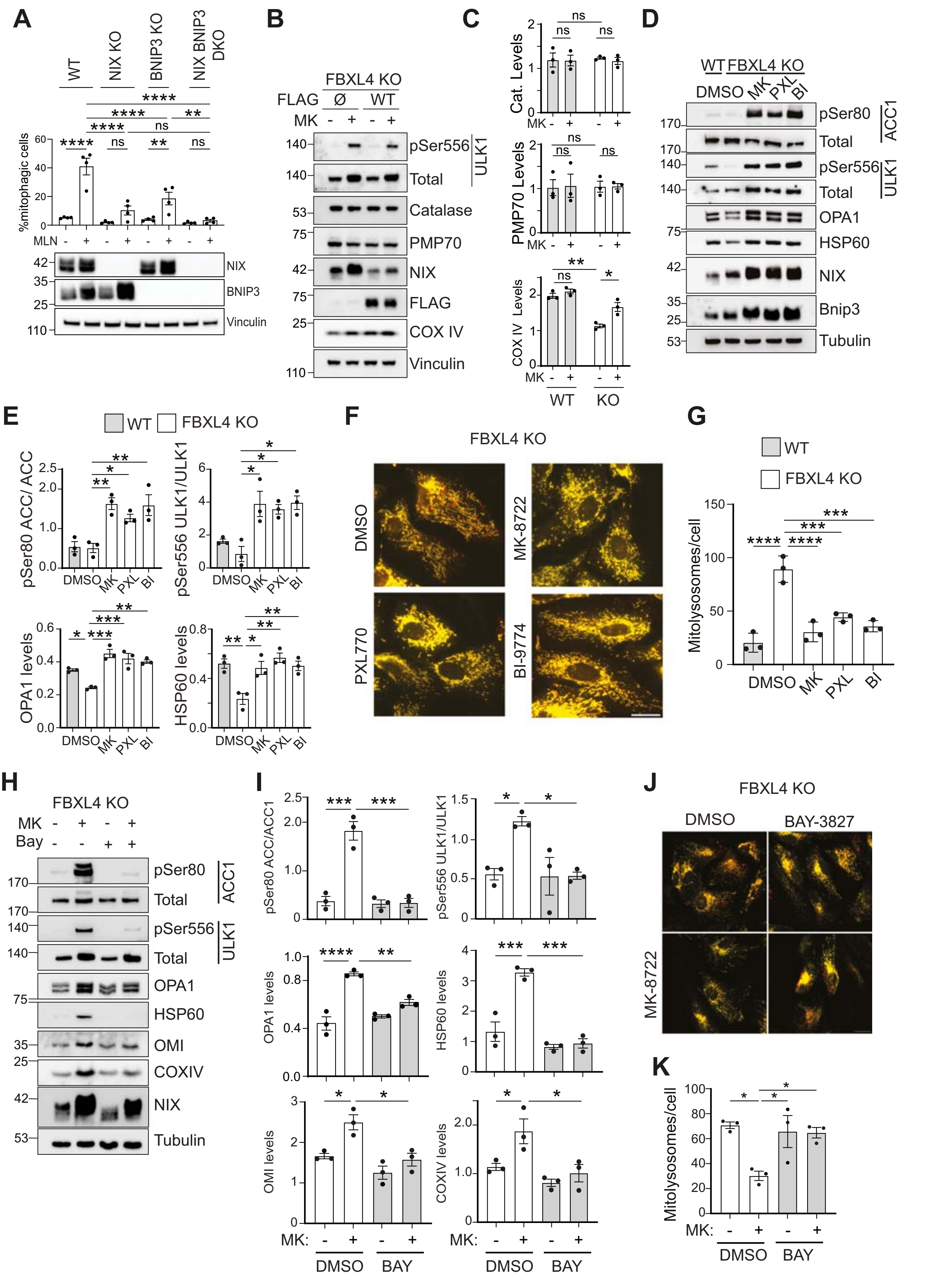
A
WT
NIX KO
BNIP3 KO
NIX BNIP3 DKO
%mitophagic cells
MLN
NIX
BNIP3
Vinculin
B
FBXL4 KO
FLAG
MK
pSer556
Total
ULK1
Catalase
PMP70
NIX
FLAG
COX IV
Vinculin
C
Cat. Levels
PMP70 Levels
COX IV Levels
WT
KO
D
WT
FBXL4 KO
DMSO
MK
PXL
BI
pSer80
Total
ACC1
pSer556
Total
ULK1
OPA1
HSP60
NIX
Bnip3
Tubulin
E
WT
FBXL4 KO
pSer80 ACC/ ACC
pSer556 ULK1/ULK1
OPA1 levels
HSP60 levels
F
FBXL4 KO
DMSO
MK-8722
PXL770
BI-9774
G
WT
FBXL4 KO
Mitolysosomes/cell
H
FBXL4 KO
MK
Bay
pSer80
Total
ACC1
pSer556
Total
ULK1
OPA1
HSP60
OMI
COXIV
NIX
Tubulin
I
pSer80 ACC/ACC1
pSer556 ULK1/ULK1
OPA1 levels
HSP60 levels
OMI levels
COXIV levels
MK:
DMSO
BAY
J
FBXL4 KO
DMSO
BAY-3827
MK-8722
K
Mitolysosomes/cell
MK:
DMSO
BAY

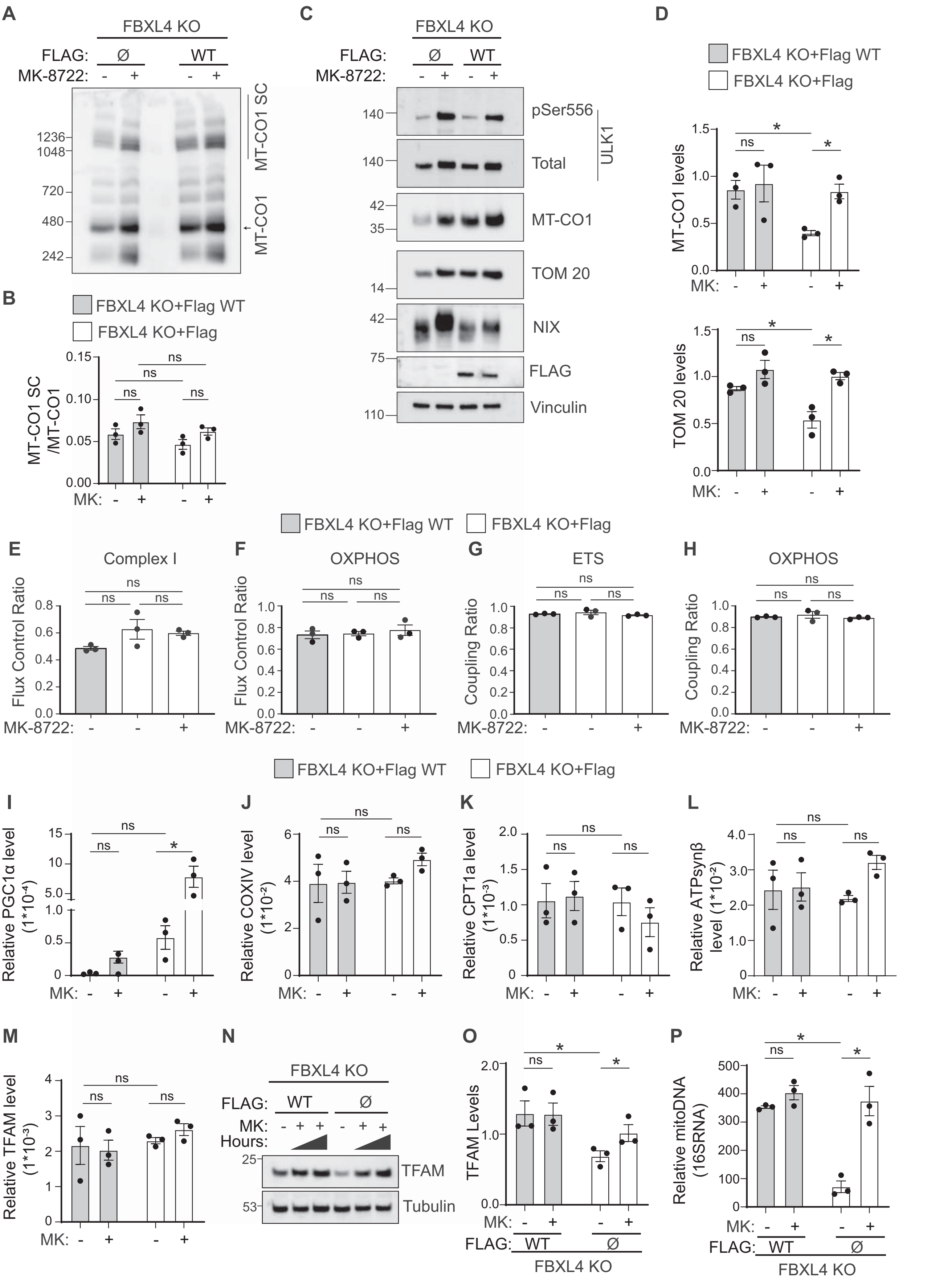
A
FBXL4 KO
FLAG: Ø WT
MK-8722: - + - +
1236
1048
720
480
242
MT-CO1 SC
MT-CO1
B
FBXL4 KO+Flag WT
FBXL4 KO+Flag
MT-CO1 SC /MT-CO1
ns
MK: - + - +
C
FBXL4 KO
FLAG: Ø WT
MK-8722: - + - +
pSer556
Total
ULK1
MT-CO1
TOM 20
NIX
FLAG
Vinculin
D
FBXL4 KO+Flag WT
FBXL4 KO+Flag
MT-CO1 levels
TOM 20 levels
MK: - + - +
E
Complex I
Flux Control Ratio
MK-8722: - - +
F
OXPHOS
Flux Control Ratio
G
ETS
Coupling Ratio
H
OXPHOS
Coupling Ratio
I
Relative PGC1α level (1*10⁻⁴)
J
Relative COXIV level (1*10⁻²)
K
Relative CPT1a level (1*10⁻³)
L
Relative ATPsynβ level (1*10⁻²)
M
Relative TFAM level (1*10⁻³)
N
FBXL4 KO
FLAG: WT Ø
MK: - + + - + +
Hours:
TFAM
Tubulin
O
TFAM Levels
MK: - + - +
FLAG: WT Ø
FBXL4 KO
P
Relative mitoDNA (16SRNA)
MK: - + - +
FLAG: WT Ø
FBXL4 KO

 © The Author(s)

Figure EV2. Effect of AMPK activation on mitochondrial respiration biogenesis.

(**A**) Crude mitochondrial fraction from FBXL4 KO and WT rescue cells with/without 10 µM MK-8722 for 48 h was resolved in a Blue Native PAGE gel and immunoblotted with MT-CO1 antibody. (**B**) Quantification of the ratio of mitochondrial complex/supercomplex (upper bands) to MT-CO1 complex (lower band with arrowhead). Data represents mean ± SEM ($n = 3$), significance calculated using two-way ANOVA and uncorrected Fisher's LSD. (**C**) Representative immunoblot of indicated protein from the whole cell lysate of FBXL4 KO and WT rescue cell with/without MK-8722 (10 µM) for 48 h. (**D**) Quantification of the immunoblot data in panel C with protein levels normalized to loading controls. Data represents mean ± SEM ($n = 3$), significance calculated using two-way ANOVA and Uncorrected Fisher's LSD. (**E**, **F**) Graph representing Flux control ratio (FCR) of Complex I (**E**) and OXPHOS (**F**) states measured from FBXL4 KO cells either expressing FLAG-empty vector or FLAG-FBXL4 WT treated with MK-8722 (10 µM) for 48 h by high-resolution respirometry. FCR denotes the ratio of oxygen flux of Complex I and OXPHOS normalized to respective ETS states. Data represents mean ± SEM ($n = 3$), significance calculated using ordinary one-way ANOVA and Tukey's multiple comparison test. (**G**, **H**) Graph representing coupling ratio (CR) of ETS (**G**) and OXPHOS (**H**) states measured from FBXL4 knockout cells either expressing FLAG-empty or FLAG-FBXL4 WT treated with MK-8722 (10 µM) for 48 h by high-resolution respirometry. CR denotes the ratio of net oxygen flux from ETS and OXPHOS normalized to respective ETS and OXPHOS states. Data represents mean ± SEM ($n = 3$), significance calculated using ordinary one-way ANOVA and Tukey's multiple comparison test. (**I–M**) Relative expression of indicated genes relative to housekeeping *ACTIN* in FBXL4 KO and WT rescue cells with/without MK-8722(10 µM) for 48 h. Data represents mean ± SEM ($n = 3$), significance calculated using two-way ANOVA and Uncorrected Fisher's LSD. (**N**) Representative immunoblot of TFAM and Tubulin of FBXL4 KO and WT rescue cell with/without MK-8722 (10 µM) for 48 and 96 h. (**O**) Quantification of the immunoblot data in panel (**N**) with protein levels normalized to loading controls. Data represents mean ± SEM ($n = 3$), significance calculated using two-way ANOVA and Uncorrected Fisher's LSD. (**P**) Relative expression of mtDNA relative to housekeeping *ACTIN* in FBXL4 KO and WT rescue cells with/without MK-8722(10 µM) for 48 h. Data represents mean ± SEM ($n = 3$), significance calculated using two-way ANOVA and uncorrected Fisher's LSD. For all the experiments, statistical significance is depicted as $*p < 0.05$, $**p < 0.01$, $***p < 0.001$, $****p < 0.0001$, and ns nonsignificant.

© The Author(s)

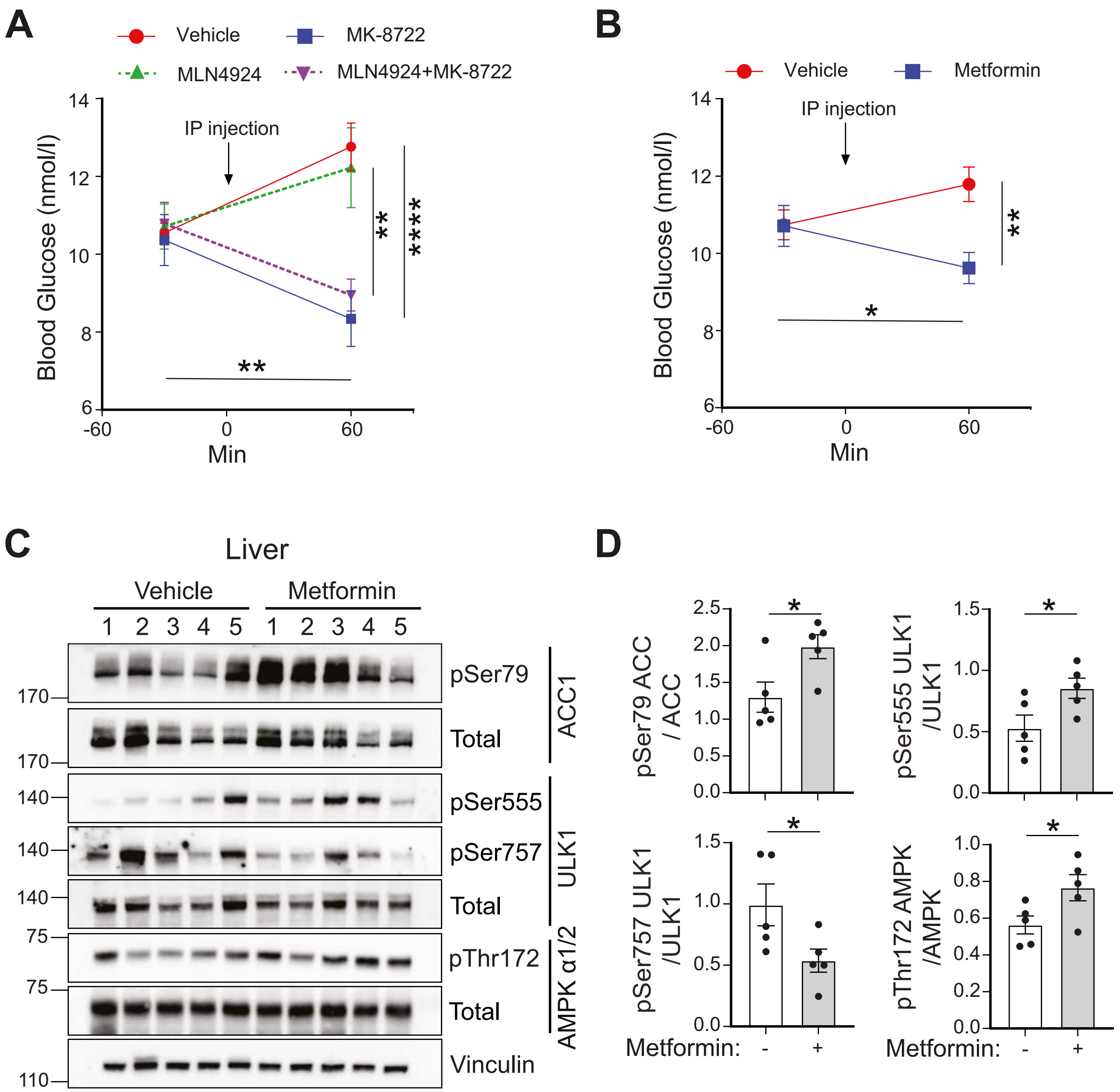

Figure EV3. Effect of MK-8722 and Metformin on blood glucose levels and AMPK signaling.

(**A**) Graph representing the blood glucose level of *mito*-QC mice monitored 30 min pre- and 60 min post day 1 interperitoneal injection of vehicle, MK-8722 (30 mg/kg), MLN4924 (50 mg/kg) by blood micro-sampling. Data represents mean ± SEM ($n = 8$ mice), significance calculated using two-way ANOVA and Tukey's multiple comparisons test. (**B**) Graph representing blood glucose level of *mito*-QC mice monitored 30 min pre- and 60 min post day 1 interperitoneal injection of vehicle or Metformin (150 mg/kg) by blood micro-sampling. Data represents mean ± SEM ($n = 10$ mice), significance calculated using two-way ANOVA and uncorrected Fisher's LSD test. (**C**) Representative immunoblots of indicated proteins from liver lysates from *mito*-QC mice injected with vehicle or metformin (150 mg/kg) for 5 days. (**D**) Quantification of the immunoblot data in panel (**C**). Data represents mean ± SEM ($n = 5$), significance calculated using an unpaired *t*-test. For all the experiments, statistical significance is depicted as *$p < 0.05$, **$p < 0.01$, ***$p < 0.001$, and ****$p < 0.0001$.

 © The Author(s)

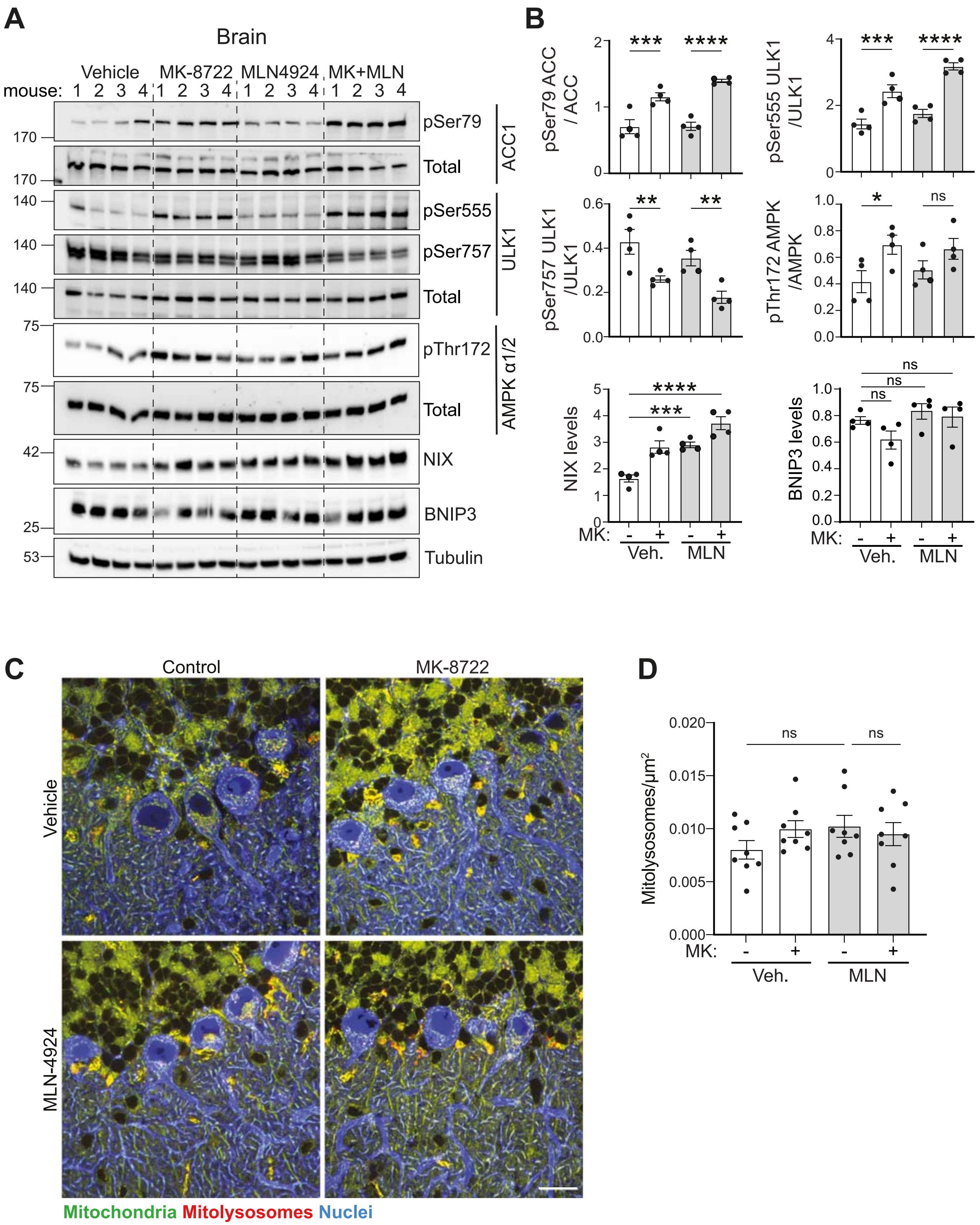
A
Brain
Vehicle
MK-8722
MLN4924
MK+MLN
mouse: 1 2 3 4 1 2 3 4 1 2 3 4 1 2 3 4
170
170
140
140
140
75
75
42
25
53
pSer79
Total
ACC1
pSer555
pSer757
Total
ULK1
pThr172
Total
AMPK α1/2
NIX
BNIP3
Tubulin
B
pSer79 ACC / ACC
pSer555 ULK1 /ULK1
pSer757 ULK1 /ULK1
pThr172 AMPK /AMPK
NIX levels
BNIP3 levels
***
****
**
*
ns
MK: - + - +
Veh.
MLN
C
Control
MK-8722
Vehicle
MLN-4924
Mitochondria Mitolysosomes Nuclei
D
Mitolysosomes/μm²
0.020
0.015
0.010
0.005
0.000
ns
MK: - + - +
Veh.
MLN

Figure EV4. Effect of MK-8722 on AMPK signaling and mitophagy in the brain.

(**A**) Representative immunoblots of indicated proteins from whole brain lysates from *mito*-QC mice dosed with vehicle, MK-8722 (30 mg/kg) and MLN4924 (MLN, 50 mg/kg) for 5 days. Each lane represents an individual mouse. (**B**) Quantification of the immunoblot data in panel (**A**) with protein levels normalized to loading controls. Data represents mean ± SEM ($n = 4$ mice), significance calculated using ordinary two-way ANOVA with Uncorrected Fisher's LSD test. (**C**) Representative confocal micrographs of Calbindin immunolabelled Purkinje neurons (Blue) in the Cerebellum from the brain sections of *mito*-QC mice dosed with vehicle, MK-8722 (30 mg/kg) and MLN4924 (MLN, 50 mg/kg) for 5 days. Red puncta indicate mitolysosomes, whereas the mitochondrial network is depicted in green. Scale bar: 20 µm. (**D**) Quantification of the number of mitolysosomes normalized to calbindin-labeled Purkinje neuron area. Data represents mean ± SEM ($n = 8$), significance calculated using ordinary one-way ANOVA and Sidak's multiple comparisons test. For all the experiments, statistical significance is depicted as *$p < 0.05$, **$p < 0.01$, ***$p < 0.001$, ****$p < 0.0001$, and ns nonsignificant.

 © The Author(s)
